# Remediation of organic pollutants by Fenton-like reactions with spinel catalysts: pathways, transformation products, and reactor perspectives

**DOI:** 10.1007/s11356-026-38096-x

**Published:** 2026-08-11

**Authors:** Viktor Husak, Liubov Soltys, Volodymyr Shvadchak, Olena Bobrova

**Affiliations:** 1 Department of Biochemistry and Biotechnology, Vasyl Stefanyk Carpathian National University, Ivano-Frankivsk, Ukraine; 2 Department of Chemistry, Vasyl Stefanyk Carpathian National University, Ivano-Frankivsk, Ukraine; 3 Division of Genetics and Plant Breeding, Czech Agrifood Research Center, Drnovska 507/73, Prague, 16100 Czech Republic; 4 Cryobiophysics Department, Institute for Problems of Cryobiology and Cryomedicine, NAS of Ukraine, Kharkiv, Ukraine

**Keywords:** Water treatment, Fenton-like catalysis, Spinel ferrite catalysts, Reactive oxygen species, Ecotoxicity, Organic pollutants

## Abstract

Spinel ferrite-catalyzed Fenton and Fenton-like advanced oxidation processes are emerging as a practical route for removing persistent organic pollutants from water, yet its selectivity, by-product formation, and scale-up behavior remain insufficiently resolved. This review consolidates recent advances in the activation of oxidants using hydrogen peroxide, peroxymonosulfate, and peroxydisulfate, and links operating conditions to the dominant oxidation pathways. It distinguishes regimes in which hydroxyl or sulfate radicals prevail from conditions that favor singlet oxygen generation or direct surface electron transfer, emphasizing the roles of pH, background anions, and matrix scavengers in controlling the kinetics and transformation profiles. Progress in catalyst development is centered on iron-rich spinels of the general composition MFe_2_O_4_, in which defect engineering, lattice symmetry modulation, and targeted doping tune Fe^2+^ to Fe^3+^ cycling and oxygen vacancy density, thereby regulating oxidant activation. Hybrid designs that couple spinels with carbon supports or semiconductor junctions enhance charge transport and enable visible-light-driven photo-Fenton operation. Durability is addressed through operando characterization of leaching, phase evolution, and surface restructuring, as well as magnetic recovery strategies that support reuse. Reactor perspectives highlight the shift from slurry testing to continuous configurations, including fixed beds, monoliths, and adsorption oxidation composites, supported by optimized oxidant dosing and hydrodynamics to mitigate mass-transfer limits and suppress halogenated by-products in chloride-rich waters. Key transformation patterns and ecotoxicity concerns are summarized, and priorities are proposed for standardized by-product reporting, durability metrics, and pilot-scale validation across variable influent chemistries.

**Graphical Abstract:**

**Figure:**
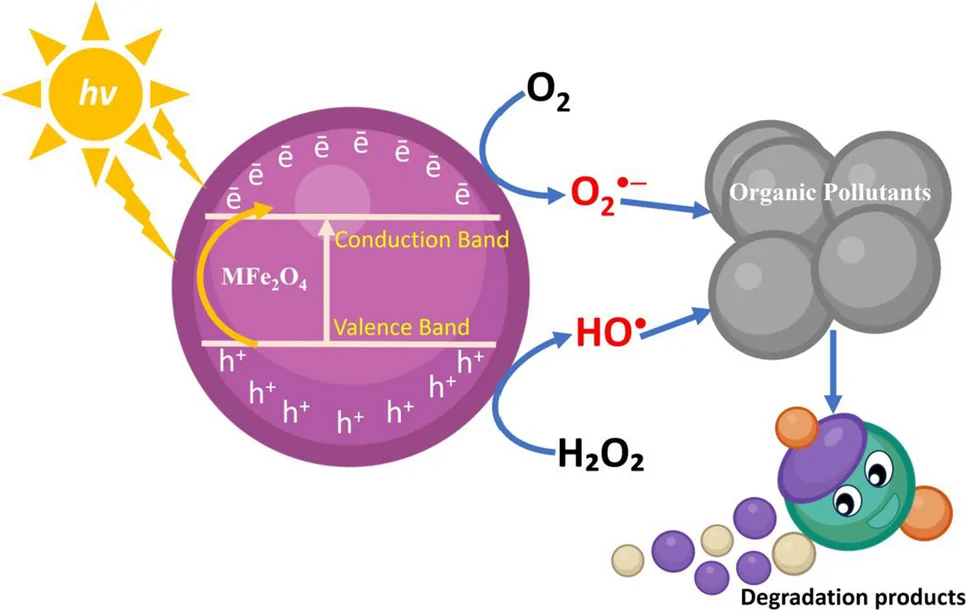

## Introduction

Fenton and Fenton-like advanced oxidation processes (AOPs) are widely used for the degradation of persistent organic pollutants because they generate highly reactive hydroxyl radicals, which can nonselectively oxidize complex aromatic and heteroatom-containing compounds that resist conventional biological treatment. Pharmaceuticals, dyes, pesticides, and endocrine-disrupting compounds are among the contaminants that often require such aggressive oxidants for effective removal (Thomas et al. 2021). Compared with ozonation or UV treatment alone, Fenton-based systems frequently achieve rapid degradation and mineralization under relatively mild operating conditions. Moreover, Fenton-like catalysts and photo-Fenton processes extend the operational pH range and enhance catalyst regeneration through improved Fe^2^⁺/Fe^3+^ cycling (Thomas et al. 2021; Husak et al. 2025). Consequently, these technologies have emerged as versatile and practical approaches for water and wastewater treatment.

Among heterogeneous Fenton catalysts, spinel ferrites (MFe₂O₄, where M is typically Cu, Co, Mn, Ni, or Zn) have attracted considerable attention because of their tunable electronic structure, chemical stability, magnetic recoverability/recyclability, and visible-light activity (Sharma et al. 2015; Gao et al. 2018; Liu et al. 2019; Hermosilla et al. 2020; Xiang et al. 2020). Their relatively narrow band gaps and favorable redox properties facilitate reactive oxygen species generation, while their magnetic nature enables efficient separation and reuse. Numerous studies have demonstrated that catalyst composition, morphology, porosity, and defect structure strongly influence catalytic performance. In particular, CuFe₂O₄, mesoporous ferrites, and other engineered spinels have shown promising activity toward the degradation of dyes, pesticides, and pharmaceuticals through both Fenton and photo-Fenton pathways (Sharma et al. 2015; Sharma and Singhal 2018; Gao et al. 2018). In addition, manganese ferrites have demonstrated favorable magnetic properties that support catalyst recovery and recyclability (Hermosilla et al. 2020).

The magnetic recoverability of spinel ferrites provides a practical advantage for water treatment applications by facilitating catalyst separation and reuse while reducing post-treatment processing requirements. This feature has been demonstrated for several ferrite-based Fenton and photo-Fenton systems, including CuFe₂O₄, CoFe₂O₄, ZnFe₂O₄, and MnFe₂O₄ catalysts used for the degradation of dyes, pesticides, and pharmaceuticals (Sharma et al. 2015; Sharma and Singhal 2018; Deng et al. 2018; Gao et al. 2018; Hermosilla et al. 2020). These characteristics make spinel ferrites attractive not only for batch operation but also for immobilized and continuous-flow configurations. Although continuous-flow studies using mixed-metal spinel ferrites remain comparatively limited, recent fixed-bed CWPO work using natural magnetite demonstrates the feasibility of long-term operation with magnetically recoverable iron-oxide catalysts, showing sustained pesticide removal and low iron leaching under prolonged operation (Lopez-Arago et al. 2024). Together, these studies support the transition from recoverable slurry ferrite catalysts toward more practical immobilized and flow-through Fenton-like systems.

Despite the growing body of literature, a comprehensive understanding of how spinel ferrite composition, structure, reactive oxygen species generation, pollutant transformation pathways, and reactor configuration collectively influence treatment performance remains limited. This review critically examines recent advances in spinel ferrite-catalyzed Fenton-like processes, with particular emphasis on catalyst design strategies, degradation mechanisms and transformation products, reactor engineering considerations, and key challenges associated with scale-up and practical implementation.

## Radical and nonradical reaction fundamentals

**Fig. 1:**
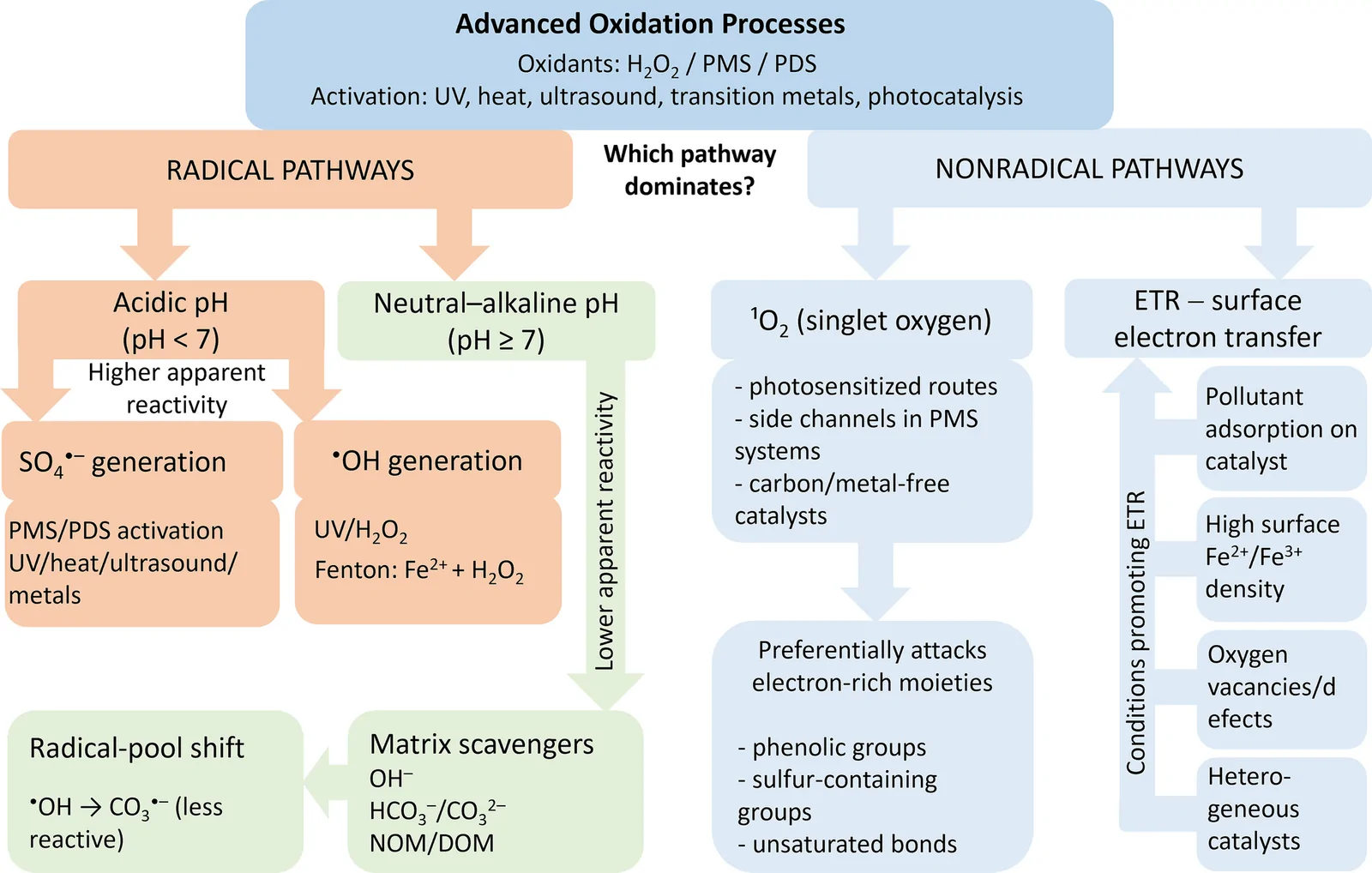
Conceptual framework distinguishing radical and nonradical oxidation routes in advanced oxidation processes for water treatment. The scheme shows how oxidant type and activation mode interact with solution pH and catalyst surface properties to determine whether hydroxyl radicals, sulfate radicals, singlet oxygen, or surface electron transfer pathways dominate. It also illustrates how radical scavenging by matrix constituents can suppress apparent reactivity and promote selective oxidation at electron-rich moieties. Abbreviations and symbols: AOPs, advanced oxidation processes; PMS, peroxymonosulfate; PDS, peroxydisulfate; UV, ultraviolet irradiation; SO_4_^•−^, sulfate radical; ^1^O_2_, singlet oxygen; ETR, surface electron transfer route; Fe^2+^, ferrous iron; Fe^3+^, ferric iron; OH^−^, hydroxide ion; HCO_3_^−^, bicarbonate; CO_3_^2−^, carbonate; NOM, natural organic matter; DOM, dissolved organic matter

Advanced oxidation processes for water and wastewater treatment rely on the in situ generation of reactive oxygen species to transform contaminants via rapid oxidation chemistry (Fig. 1). In Fenton and persulfate-based schemes, the dominant oxidizing pool typically consists of hydroxyl radicals and sulfate radicals, but many systems evolve toward nonradical routes, particularly singlet oxygen and surface-mediated electron transfer, when radical availability is suppressed by the water matrix or when catalysts favor interfacial pathways (Xia et al. 2020; Scaria and Nidheesh 2022; Danyliuk et al. 2024, 2025).

### Key reactive species and their mechanistic signatures

Hydroxyl radicals (•OH) are central to classical Fenton chemistry and UV-activated hydrogen peroxide systems. Their very high oxidation potential (*E*° ≈ 2.8 V vs. SHE) and diffusion-limited reactivity enable rapid attack on most organic molecules, explaining their effectiveness in pollutant degradation and mineralization (Ding et al. 2020; Scaria and Nidheesh 2022; Husak et al. 2025). However, their short lifetime and nonselective kinetics also make them highly vulnerable to competitive scavenging by background solutes, so treatment efficiency becomes strongly dependent on matrix composition and local mass transfer around reactive sites (Brienza and Katsoyiannis 2017; Scaria and Nidheesh 2022).

Sulfate radicals (SO₄•⁻) are produced from peroxymonosulfate or peroxydisulfate activation and exhibit a high oxidation potential (*E*° ≈ 2.44 V vs. SHE), comparable to that of hydroxyl radicals. In addition, SO₄•⁻ radicals generally possess longer lifetimes and greater selectivity than •OH radicals, making them less susceptible to scavenging in complex water matrices (Scaria and Nidheesh 2022). Their reaction patterns frequently include single electron transfer to aromatics and electrophilic addition mechanisms, which can introduce different selectivity relative to hydroxyl radical-dominated systems (Brienza and Katsoyiannis 2017; Scaria and Nidheesh 2022).

Nonradical oxidation becomes important when freely diffusing radicals are depleted or when the catalytic interface controls oxidation (Fig. 1). Singlet oxygen is a comparatively selective oxidant that reacts preferentially with electron-rich moieties and unsaturated bonds, and it is often linked to photosensitized processes or to side reactions during oxidant self-decomposition (Liu et al. 2021; Yan et al. 2023). Although its bimolecular rate constants are usually lower than those of radical reactions, its selectivity can reduce nonspecific oxidation and can shift transformation product spectra toward fewer highly oxidized side products (Xie et al. 2022; Yan et al. 2023). Surface electron transfer routes represent another nonradical family in heterogeneous systems where pollutants adsorb and electrons are transferred directly from the contaminant to the oxidant through catalyst surface states, which can sustain oxidation even when the bulk radical pool is heavily scavenged (Liu et al. 2021; Yan et al. 2023).

### Oxidant choice and primary radical generation regimes

Hydrogen peroxide-based processes, including UV/H_2_O_2_ and Fenton-type activation, predominantly generate hydroxyl radicals. These systems often reach maximum efficiency under acidic conditions where iron-mediated activation is favored and where hydroxyl radical scavenging by carbonate species is less pronounced (Lutze et al. 2015; Guan et al. 2020; Husak et al. 2025). Under optimized conditions, hydroxyl radical chemistry can achieve rapid degradation and, with adequate oxidant dosing, extensive mineralization, but the same nonselectivity that enables broad reactivity also increases oxidant loss to matrix scavengers and to parasitic pathways (Ding et al. 2020; Scaria and Nidheesh 2022).

Peroxymonosulfate and peroxydisulfate activation generate sulfate radicals and, depending on conditions, a mixed radical pool in which sulfate radicals and hydroxyl radicals coexist due to interconversion reactions. Persulfate systems are often described as operationally broader in pH than classical Fenton chemistry, yet this flexibility is conditional because pH simultaneously controls oxidant speciation, radical hydrolysis, and competitive scavenging (Lutze et al. 2015; Guan et al. 2020). Under near-neutral or slightly alkaline conditions, partial hydrolysis of sulfate radicals can contribute to a dual radical mechanism, which can be beneficial for degradation but complicates selectivity control and by-product prediction (Yan et al. 2023).

### pH effects and radical interconversion

pH is a master variable because it dictates oxidant speciation, metal site availability in Fenton-type pathways, and the formation and stability of scavenging species. In hydrogen peroxide systems, acidic pH promotes efficient hydroxyl radical production and limits ferric hydroxide precipitation, supporting sustained radical generation and higher oxidation rates (Guan et al. 2020). As pH increases toward neutral and alkaline values, bicarbonate and carbonate concentrations rise and become strong competitors for hydroxyl radicals, shifting the oxidant pool toward less reactive carbonate-derived radicals and lowering effective oxidative capacity for many targets (Lutze et al. 2015; Faheem et al. 2020).

In persulfate and peroxymonosulfate systems, higher pH can increase the apparent rate of oxidant activation for some configurations because PMS dissociation can support higher radical generation. At the same time, elevated pH promotes sulfate radical interconversion and hydrolysis, and it changes chloride speciation and side chemistry, which can shift systems toward unwanted oxidized chlorine products under certain conditions (Guan et al. 2020; Lutze et al. 2015). These coupled effects mean that pH optimization should be tied to both kinetic performance and by-product control rather than to conversion rate alone (Lutze et al. 2015; Guan et al. 2020).

### Bicarbonate and carbonate as scavengers and pathway modifiers

Bicarbonate plays a dual role in AOPs as a buffering agent and as a radical scavenger. In hydroxyl radical-dominated processes, bicarbonate consumes hydroxyl radicals to form carbonate radicals, which have lower reactivity toward many pollutants and thus reduce treatment efficiency at comparable oxidant doses (Lutze et al. 2015; Faheem et al. 2020). In PMS and PDS systems, bicarbonate also reacts with sulfate radicals, again shifting the reactive pool toward less aggressive species and decreasing the fraction of radicals available for target oxidation (Ahn et al. 2021). Beyond simple scavenging, bicarbonate can modify sulfate radical and hydroxyl radical interconversion pathways, thereby changing the relative contributions of each radical type and altering transformation product distributions (Lutze et al. 2015; Faheem et al. 2020).

Although inhibition is common, bicarbonate effects are not universally negative because carbonate radicals can participate in the selective oxidation of certain substrates, and buffering can stabilize the operating pH. Reported enhancements for specific phenolic targets highlight that matrix effects must be interpreted through the lens of substrate-dependent kinetics and not as a single monotonic trend (Yang et al. 2022; Tatarchuk et al. 2024, 2025).

### Chloride chemistry and the risk of chlorate formation

Chloride is particularly important because it can alter radical pools and promote reactive chlorine intermediates that change both kinetics and by-product outcomes. In hydrogen peroxide-based systems, chloride reacts with hydroxyl radicals to form chlorine radicals and related species; however, these intermediates often undergo rapid termination via reactions with residual hydrogen peroxide, thereby limiting net conversion to chlorate (Guan et al. 2020). This behavior is one reason why hydrogen peroxide-based AOPs can be advantageous when minimizing oxidized chlorine by-products is a priority (Guan et al. 2020).

In PMS and PDS systems, chloride effects are more conditional. Moderate chloride can enhance PMS activation and produce reactive chlorine species that sometimes accelerate oxidation of particular organics, but high chloride levels or conditions that accelerate radical interconversion can drive chlorine radical accumulation and subsequent conversion toward chlorate (Lutze et al. 2015; Ahn et al. 2021). The balance is further shaped by pH and by the presence of other matrix constituents, which reinforces the need for explicit chloride management and by-product tracking when persulfates are applied to saline or industrial waters (Li et al. 2017; Guan et al. 2020).

From an application standpoint, radical-dominated regimes favor rapid, broad-spectrum degradation but often generate complex intermediate mixtures and exhibit low oxidant utilization in scavenger-rich waters. Nonradical regimes, driven by singlet oxygen or surface electron transfer, can improve selectivity and maintain activity in challenging matrices, but they may slow mineralization if oxidation proceeds through narrower pathways that do not efficiently open aromatic rings (Liu et al. 2021; Yan et al. 2023). Therefore, selecting between H_2_O_2_ and PMS or PDS should be framed as a choice between different sensitivity profiles to pH and scavengers, different risks of oxidized chlorine by-products, and different tradeoffs between rapid conversion and controlled transformation chemistry (Lutze et al. 2015; Guan et al. 2020).

## Spinel ferrite catalyst design, architecture, and stability

Spinel ferrites with the general formula MFe_2_O_4_ provide a modular materials platform for heterogeneous Fenton and persulfate catalysis because their activity can be tuned through composition, cation distribution, defect chemistry, morphology, and interfacial engineering. In contrast to the “Radical and nonradical reaction fundamentals” section, which outlines how oxidant identity and water matrix govern radical and nonradical routes, this section focuses on how catalyst structure and processing determine surface site density, electron transport capability, magnetic recoverability, and resistance to deactivation and metal release during repeated operation (Badreldin et al. 2020; Tatarchuk 2024).

The divalent metal M controls the balance between structural stability, redox flexibility, and magnetic response. Substitution at tetrahedral and octahedral sites alters cation inversion and local coordination environments, which changes the availability of surface-exposed metal centers and their ability to participate in fast interfacial electron transfer to oxidants. Shifting from normal toward inverse spinel arrangements has been associated with higher densities of catalytically accessible surface sites and improved turnover in oxidation reactions, which motivates composition screening as an initial step in catalyst selection (Pastor et al. 2022).

Mixed-metal spinels further broaden the accessible design space. Cooperative redox couples, for example, Fe with Cu, Mn, or Co, can increase electron mobility and strengthen catalytic cycling, but they also introduce new stability constraints, including preferential dissolution of specific cations under aggressive oxidizing environments. As a result, composition optimization must be paired with stability testing rather than relying on initial activity alone (Gao et al. 2018; Tan et al. 2017; Deng et al. 2018; Li et al. 2019; Danyliuk et al. 2023).

**Fig. 2:**
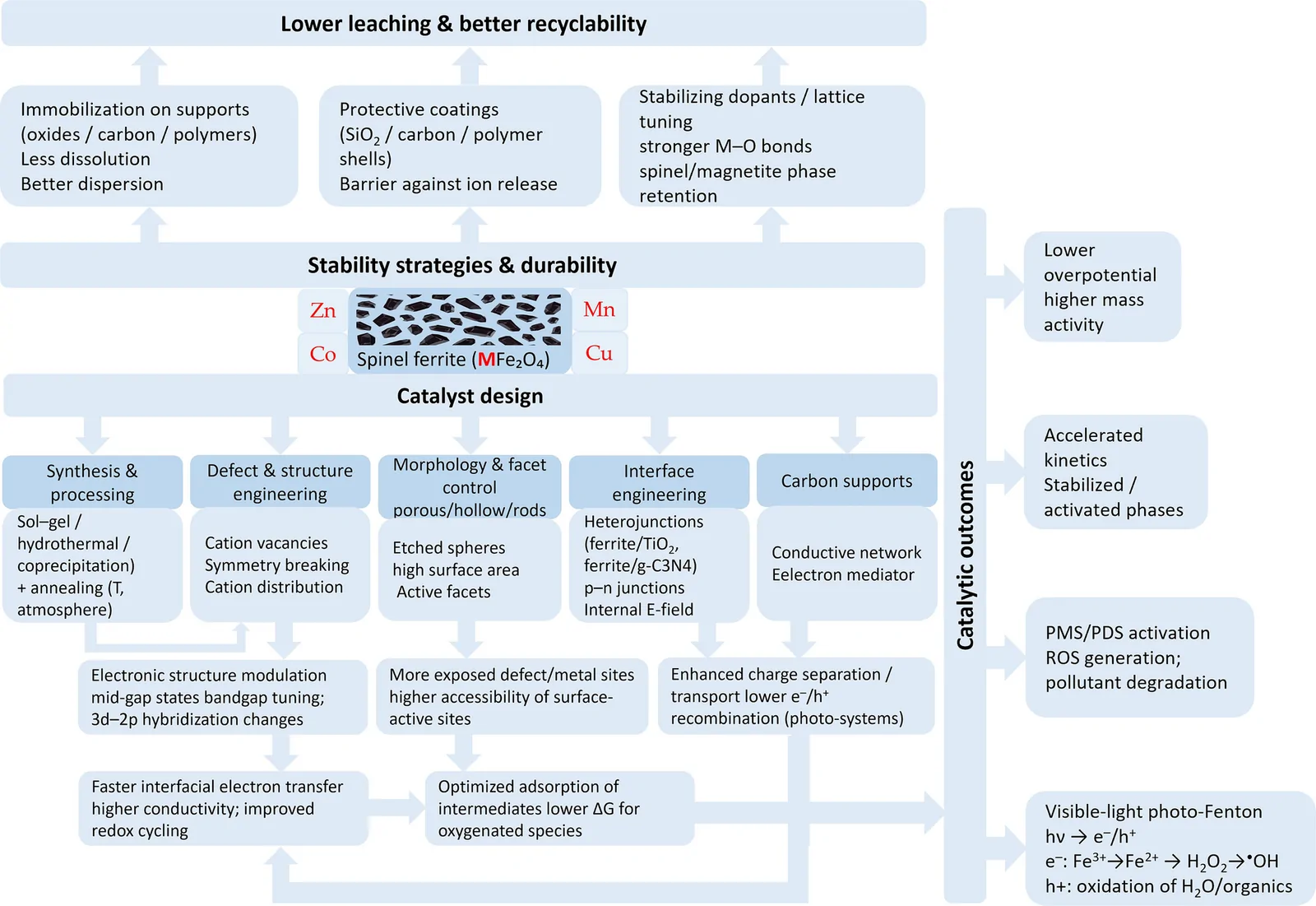
Integrated design and durability roadmap for spinel ferrite catalysts in Fenton and persulfate-based advanced oxidation. The scheme summarizes how composition, lattice structure, synthesis route, defect engineering, morphology, heterojunction formation, and conductive carbon coupling modulate electronic structure, interfacial electron transfer, charge separation, reactive-intermediate adsorption, and ROS generation. It also highlights stability strategies, including support immobilization, protective silica, carbon, or polymer shells, and stabilizing dopants, which reduce ion release, preserve magnetic phases, and improve recyclability. Abbreviations and symbols: MFe_2_O_4_, spinel ferrite with a divalent metal cation M; M–O, metal oxygen bond; SiO_2_, silicon dioxide; ROS, reactive oxygen species; TiO_2_, titanium dioxide; g-C_3_N_4_, graphitic carbon nitride; p–n junction, p-type to n-type semiconductor junction; E-field, electric field; Δ*G*, Gibbs free energy change; hν, photon energy under light irradiation; e^−^, electron; h^+^, hole; H_2_O_2_, hydrogen peroxide

Defect engineering has become a central strategy for intensifying spinel ferrite performance because oxygen vacancies, cation vacancies, and symmetry-breaking distortions can create coordinatively unsaturated sites and introduce localized electronic states that improve interfacial charge transfer (Fig. 2). Oxygen vacancies can lower adsorption free energies for key intermediates and increase lattice and surface conductivity by acting as electron donors, thereby raising the probability of productive oxidant activation events at the solid surface (Debnath et al. 2020; Wang et al. 2021a; Tatarchuk 2024).

Aliovalent and high-valence dopants are frequently used to promote vacancy formation and reshape the local electronic structure. Such dopant-driven defect generation can simultaneously increase active site density and enhance charge carrier mobility, which is particularly important for ferrites that otherwise suffer from limited conductivity and surface passivation under cycling conditions (Badreldin et al. 2020; Zhang et al. 2025).

Defect control is inherently synthesis dependent. Sol–gel routes, hydrothermal processing, and annealing under controlled atmospheres can introduce and stabilize targeted defect populations, while post-treatments can modulate defect density through sintering and reoxidation. Low-temperature routes using surfactants have been shown to produce nanoparticles with high densities of surface defects, which can subsequently be tuned through thermal processing to balance activity and durability (Dong et al. 2019b; Hermosilla et al. 2020; Ibrahim et al. 2016).

Catalyst morphology determines the number of engineered sites that are actually accessible during operation (Fig. 2). Hollow spheres, porous networks, and one-dimensional nanostructures increase the external surface area and shorten diffusion pathways, thereby improving the utilization of surface defects and reducing sensitivity to aggregation. Controlled etching has been reported to generate broken nanomicrospheres with high oxygen vacancy densities that show enhanced activation performance, illustrating how morphology can be used as a practical multiplier of defect engineering benefits (Salih and Mahmood 2023; Tatarchuk 2024).

Facet engineering introduces an additional control dimension because different crystallographic planes can differ in defect-formation propensity and cation-termination patterns. Preferential exposure of high activity facets, for example, those associated with higher defect concentrations, can improve intrinsic surface reactivity without changing bulk composition, but it requires synthesis routes that maintain facet stability during calcination and cycling (Tatarchuk 2024).

Single-phase ferrites often exhibit limited charge mobility and fast recombination under illumination. Heterojunction formation in semiconductors can create built-in electric fields that promote directional charge separation and prolong carrier lifetimes, thereby improving the effectiveness of visible-light-driven photo-Fenton operation (Fig. 2). Representative designs include ZnFe_2_O_4_ coupled with TiO_2_ or with graphitic carbon nitride, where band alignment and interfacial fields facilitate electron transfer across the junction and intensify photo-assisted catalytic cycling (Garcia-Muñoz et al. 2019; Wang et al. 2020; Li et al. 2025).

Carbon integration has emerged as one of the most effective approaches for improving spinel ferrite performance in Fenton-like and persulfate-based oxidation systems. Carbonaceous materials can enhance conductivity, facilitate interfacial electron transfer, suppress charge-carrier recombination, increase adsorption capacity, and reduce nanoparticle aggregation. In addition, carbon components often improve catalyst stability by limiting metal leaching and surface passivation during repeated redox cycling. Among carbon-based modifiers, reduced graphene oxide (rGO) has received considerable attention because its high electrical conductivity and large specific surface area promote rapid electron transport and efficient charge separation (Hassani et al. 2018; Sebah and Belmouden 2025). Spinel ferrite/rGO composites have demonstrated enhanced oxidant activation, accelerated reactive oxygen species generation, and improved photocatalytic performance compared with the corresponding pristine ferrites, as shown for CoFe₂O₄-rGO in sono-Fenton-like dye removal (Hassani et al. 2018). Similar benefits have been reported for graphene-supported ferrites, where strong interfacial contact between the conductive carbon network and ferrite nanoparticles facilitates electron mobility and improves catalyst dispersion. Carbon nanotubes (CNTs) provide an alternative conductive scaffold that can increase accessible surface area while creating efficient electron-transfer pathways between ferrite active sites and oxidants. CNT-supported magnetic Fenton and photo-Fenton catalysts have been reported to improve dye wastewater treatment, discoloration, TOC abatement, and catalyst stability, while NiFe₂O₄/MWCNT composites have also shown enhanced photocatalytic degradation of pharmaceutical contaminants compared with pristine ferrite catalysts (García et al. 2017; Varghese et al. 2023). More recently, carbon quantum dots (CQDs) have emerged as multifunctional modifiers capable of improving visible-light harvesting, facilitating charge transfer, and promoting reactive oxygen species generation. Owing to their abundant surface functional groups and up-conversion photoluminescence properties, CQDs can enhance ferrite-based photocatalytic activity while maintaining magnetic recoverability, as reported for CQDs/ZnFe₂O₄ and CoFe₂O₄–CQD composites (Huang et al. 2017b; Ahmadian-Fard-Fini et al. 2017). Biochar has attracted growing interest as a low-cost and sustainable support material. Its porous structure, surface functional groups, and electron-shuttling capability can enhance pollutant adsorption and oxidant activation while reducing catalyst agglomeration. Ferrite–biochar composites are particularly promising for practical water-treatment applications because they combine catalytic activity with low material cost and straightforward recovery; for example, biochar-supported cobalt ferrite has been used for persulfate activation and pollutant degradation (Gu et al. 2023; Zhi et al. 2022). Activated carbon remains one of the most widely used supports owing to its high surface area and adsorption capacity. Ferrite–activated carbon composites can concentrate pollutants near catalytic sites, improve oxidant utilization efficiency, and increase catalyst stability, as shown for activated carbon/CoFe₂O₄ in reactive dye removal (Heidari et al. 2019). Carbon-coated ferrites, including carbon-coated hollow CuFe₂O₄ spheres and Fe₃O₄@C materials, further demonstrate how conductive carbon shells can improve charge transport, suppress surface passivation, and reduce metal leaching while preserving magnetic separability (Zhang et al. 2014; Guo et al. 2017). Overall, carbon modification has evolved from simple protective coatings to sophisticated conductive and photoactive architectures that simultaneously improve adsorption, electron transport, oxidant activation, and catalyst durability. These multifunctional benefits make carbon–ferrite composites a key direction for the development of next-generation Fenton-like catalysts.

Durable deployment requires that high activity designs also resist dissolution, phase transformation, and irreversible aggregation. Leaching can occur when reactive surface sites corrode under acidic or strongly oxidizing conditions, and it can be intensified by nanoscale restructuring during repeated redox cycling. Practical mitigation strategies include immobilization on stable supports, surface modification with protective shells, and stabilizing dopants that strengthen metal oxygen bonding while preserving catalytic accessibility (Manos et al. 2020; Ganjali et al. 2022).

Magnetic recovery is a defining advantage of spinel ferrites and should be protected as a functional property during design. Maintaining magnetite-like phases and avoiding conversion to less magnetic oxides improves separability and supports reuse. Selective doping has been reported to stabilize magnetic phases and reduce phase transformation during synthesis and cycling, which links composition and stability design directly to operational cost and sustainability (Li et al. 2019; Maji and Dosanjh 2023).

To compare catalyst concepts across studies, performance reporting should distinguish intrinsic site reactivity from architecture-driven gains such as increased adsorption, improved dispersion, or enhanced light harvesting. At a minimum, the evaluation should include metal leaching, residual activity, magnetic recoverability after cycling, and evidence of phase and surface stability. Operando or cycling aligned characterization is particularly valuable for identifying whether activity loss arises from surface passivation, defect annihilation, particle growth, or composition drift (Pastor et al. 2022; Huang et al. 2022).

Overall, the most robust spinel ferrite designs combine controlled cation distribution and defect populations with morphologies that maximize accessible sites, interfacial architectures that enhance charge transport when photo-assisted, and explicit stability engineering that limits leaching while preserving magnetic recovery. This integrated materials approach enables catalysts that are not only active in model systems but also resilient under the cycling and separation demands required for practical water treatment.

Future progress will depend on standardized benchmarking that decouples intrinsic site activity from morphology and composite effects, and on reporting practices that integrate phase and surface characterization before and after cycling. Such harmonization will enable more reliable catalyst selection for pilot-scale evaluation and will accelerate the transition of spinel ferrite catalysts from laboratory demonstrations to robust water treatment technologies (Huang et al. 2022; Pastor et al. 2022).

## Reactor engineering and scale-up

**Fig. 3:**
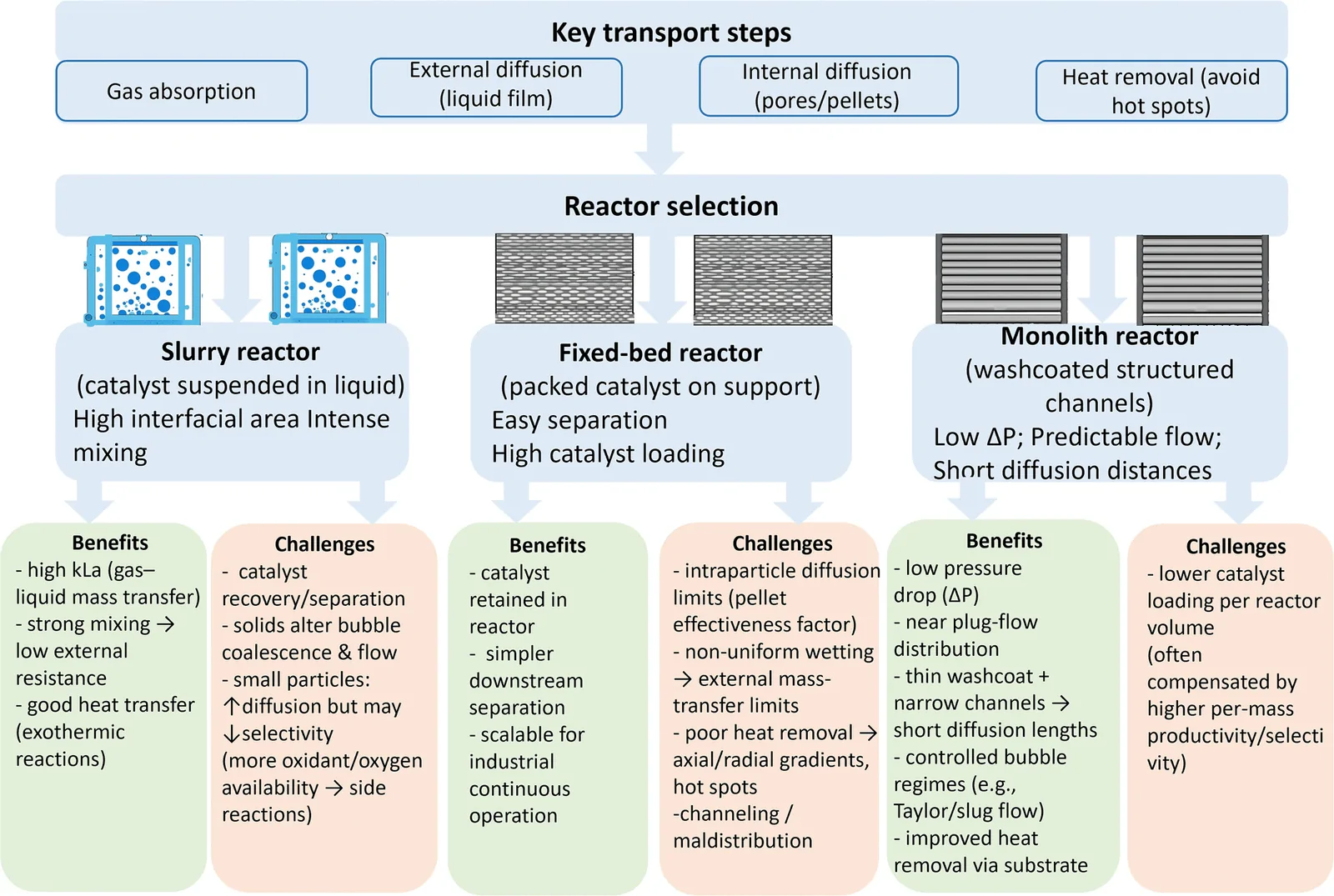
Engineering map for selecting and scaling liquid–solid catalytic reactors in advanced oxidation systems. The scheme compares slurry reactors, fixed-bed reactors, and washcoated monolith reactors in terms of interfacial area, catalyst loading, hydrodynamics, pressure drop, wetting and maldistribution, external mass transfer, internal diffusion, heat removal, and downstream catalyst separation. Abbreviations and symbols: kLa, volumetric gas–liquid mass-transfer coefficient; Δ*P*, pressure drop

Efficient catalytic processes in multiphase systems demand careful integration of reactor design, hydrodynamic control, and mass-transfer management to maximize conversion while preserving selectivity and catalyst lifetime. In liquid–solid reactors, which are used for advanced oxidation and water treatment applications, the engineering challenges revolve around ensuring proper mixing, uniform reagent distribution, and minimized diffusion limitations. Reactor type selection (slurry, fixed‐bed, or monolith) plays a decisive role in determining performance characteristics (Fig. 3), while dosing strategies for reactive chemicals such as H_2_O_2_ are critical in advanced oxidation processes like the photo‐Fenton reaction. The interplay among these factors defines the reactor’s catalytic efficiency, scale‐up feasibility, and operating stability (Fig. 3) (Joseph 2016).

Slurry reactors operate by suspending finely divided catalyst particles in a liquid medium, thereby maximizing the contact area between reactants and the catalyst. Such designs inherently promote high mass-transfer coefficients because vigorous mixing, often induced by mechanical agitation or gas sparging, ensures continuous renewal of the liquid film at the catalyst surface (Joseph 2016). In these reactors, catalyst particle sizing is critical; smaller particles improve external and internal diffusion rates but can lead to lower selectivity due to increased oxygen availability and potentially undesirable side reactions (Joseph 2016). Moreover, although the homogeneous distribution of catalysts within the slurry can lead to high reaction rates and excellent interfacial contact, catalyst recovery and separation remain challenging, impacting process economics and operational complexity (Audino et al. 2019; Králik et al. 2025). The turbulent hydrodynamics in slurry reactors can enhance heat and mass transfer, which is advantageous when oxidant activation and pollutant degradation are limited by interfacial transport. However, the very qualities that render slurry reactors effective for rapid conversions also complicate control over selectivity, particularly when competitive side reactions are present (Joseph 2016).

Fixed‐bed reactors employ catalysts that are immobilized on support materials inside a packed bed. In these systems, the catalyst particles remain stationary while the gaseous and liquid phases percolate through the interstitial spaces. The primary advantage of fixed‐bed reactors is the ease of catalyst handling and the inherent simplification of downstream separation, as the catalyst is not dispersed in the product stream (Lopes and Rodrigues 2016; Králik et al. 2025). Fixed‐bed setups tend to afford high catalyst loadings and can be scaled up economically, making them attractive for industrial applications. Nevertheless, the fixed arrangement of catalyst particles gives rise to significant internal mass-transfer resistances due to the diffusion limitations within the catalyst pellets (Degirmenci and Rebrov 2016; Králik et al. 2025).

Monolith reactors represent a class of structured reactors whereby catalysts are deposited or washcoated on substrates that contain an array of parallel, interconnected channels. The structured design of monoliths allows for a highly predictable flow distribution, typically approximating plug flow conditions, and yields markedly lower pressure drops compared to conventional fixed‐bed reactors (Joseph 2016; Szymańska et al. 2021). The extremely narrow channels, often in the range of 1–2 mm, in conjunction with thin catalytic washcoat layers (typically around 70 μm), significantly reduce both the external and internal diffusion distances, enabling markedly enhanced mass-transfer rates (Buwa et al. 2016; Joseph 2016). Engineered geometries enable the development of desirable flow regimes and mitigation of issues related to channeling. Despite the enhanced mass transfer and catalyst utilization, monolith reactors often have a lower catalyst loading per unit reactor volume compared to conventional fixed beds; yet, this drawback is frequently offset by the higher per‐catalyst mass productivity and improved selectivity (Parvulescu et al. 2021).

The reactor hydrodynamics and mass-transfer characteristics fundamentally determine the efficiency and selectivity of catalytic processes. In slurry reactors, vigorous mixing provided by high-energy agitators or optimized sparger designs enhances mass-transfer coefficients on the liquid side. The turbulent flow regimes inherent in slurry systems serve to minimize external resistances (Králik et al. 2025). Yet, the presence of suspended solids introduces additional complexities in the flow field, as the solid phase can alter the effective diffusivity within the liquid (Králik et al. 2025).

Fixed‐bed reactors, on the other hand, experience fundamentally different hydrodynamic behavior. The flow through the interstitial voids of a packed bed is often approximated as plug flow, although actual profiles may be far from ideal due to channeling and uneven liquid distribution (Lopes and Rodrigues 2016; Králik et al. 2025). In these systems, mass transfer is primarily governed by diffusive and convective phenomena occurring in the liquid phase. The limited convection can lead to significant concentration gradients, with external mass-transfer resistances becoming rate limiting in many cases (Králik et al. 2025). Furthermore, the effective diffusivity within the catalyst pellets is characterized by an effectiveness factor that captures the degree of intraparticle diffusion resistance; this factor is typically reduced as catalyst particle size increases, thus adversely affecting overall conversion rates (Cornejo and Hayes 2021; Králik et al. 2025).

Hydrogen peroxide is widely employed as an oxidant in AOPs, particularly in Fenton and photo‐Fenton systems used for water treatment. The dosing of H_2_O_2_ is a critical parameter, as it controls the generation of ^•^OH, which are responsible for the degradation of recalcitrant pollutants. However, the dosing strategy also has a profound impact on the overall efficiency of the process due to competing side reactions and replenishment requirements. Several dosing strategies have been evaluated experimentally. Batch dosing, whereby the entire H_2_O_2_ charge is introduced at once, typically leads to a rapid generation of hydroxyl radicals, resulting in a fast initial pollutant degradation rate. However, this strategy can also lead to rapid consumption and depletion of H_2_O_2_, as well as the onset of scavenging reactions where excess H_2_O_2_ reacts with ^•^OH radicals, thereby reducing overall process efficiency (Xiangwei et al. 2021). In one study, a batch dosing approach resulted in the fastest initial pollutant removal but led to an earlier exhaustion of H_2_O_2_, which ultimately did not maximize total mineralization efficiency (Yu et al. 2020; Xiangwei et al. 2021). Continuous dosing strategies have been explored as an alternative, wherein the total H_2_O_2_ dose is added gradually over the course of the reaction. While this method helps maintain H_2_O_2_ availability over longer reaction times and may reduce peak concentrations that trigger scavenging effects, it does not consistently outperform batch dosing in terms of TOC removal (Yu et al. 2020). The trade-off in continuous dosing is that while pollutant degradation may be sustained for longer periods, the overall reaction rate may be diminished if the dosing is too slow relative to the demand for hydroxyl radicals (Yu et al. 2020).

The choice between slurry, fixed‐bed, and monolith reactors, as well as the selection of an optimal H_2_O_2_ dosing regime, must be considered in an integrated manner that simultaneously addresses fluid mechanics, mass-transfer limitations, and the catalytic kinetics of the system. The successful implementation of such tailored designs can lead to catalytic processes with superior conversion rates, enhanced selectivity, and improved operational sustainability. Future research should continue to explore the synergies between reactor hydrodynamics, transport phenomena, and adaptive dosing strategies by incorporating advanced simulation tools and real-time sensor networks to further optimize reactor performance at both laboratory and industrial scales (Audino et al. 2019; Joseph 2016; Szymańska et al. 2021; Xiangwei et al. 2021; Králik et al. 2025).

## Transformation products and ecotoxicology

The transformation of complex organic pollutants during environmental processes and water treatment often leads to the formation of secondary products that display markedly different chemical and toxicological properties from their parent compounds (Suman et al. 2022). In recent decades, emerging concerns regarding the ecotoxicological effects of transformation products (TPs) have stimulated considerable research into both their formation mechanisms and the risk they pose to human health and aquatic ecosystems (Chen et al. 2015a). In particular, quinones generated from the oxidation of phenols and halogenated by-products produced in chloride-containing environments have been identified as critical targets due to their high toxicity and persistence (Dai et al. 2015; Gao et al. 2017).

Transformation products result from a variety of chemical, photolytic, biological, and catalytic processes that alter the parent structure and physicochemical properties of organic pollutants (Suman et al. 2022). For instance, phenolic compounds such as triclosan, tetrabromobisphenol A, and other halogenated phenols are subject to sequential reactions, including debromination, dechlorination, hydroxylation, and ring opening, that yield intermediate species, among which quinones are frequently observed (Dai et al. 2015). The transformation process is often highly dependent on the reaction conditions, including pH, temperature, and the presence of catalysts or co-reactants. In particular, the presence of chloride ions (Cl^–^) can influence the formation of halogenated by-products with distinct reactivity profiles (Yoom et al. 2018; Xiang et al. 2021). The complexity of transformation reactions poses significant challenges in predicting toxic outcomes and in implementing effective reduction strategies (Suman et al. 2022).

The oxidation of phenolic compounds typically proceeds via a series of electron transfer reactions that yield reactive intermediates such as semiquinone radicals and ultimately stable quinone derivatives (Gao et al. 2017). Quinones are characterized by an electrophilic carbonyl functionality that can react readily with nucleophilic biomolecules such as proteins and DNA, thereby potentially inducing deleterious biological effects (Prasse et al. 2018; Suman et al. 2022). The structure–activity relationship (SAR) of chlorinated phenols, for instance, shows that higher degrees of halogenation often correlate with increased toxicity and slower degradation kinetics, directly implicating quinone formation in the overall ecotoxicological risk (Dai et al. 2015; Gao et al. 2017). Moreover, the reactive oxygen species produced in conjunction with quinone formation further exacerbate cellular damage, contributing to the observed acute and chronic toxicity in aquatic environments (Gao et al. 2017; Prasse et al. 2018; Schmiemann et al. 2023).

Chloride ions, ubiquitous in many natural and treated water systems, play a pivotal role in influencing the chemistry of organic pollutant degradation (Dai et al. 2015; Yoom et al. 2018). During disinfection and oxidation reactions, especially in the presence of chlorine-based agents, organic substrates can react with Cl^•^ to form halogenated by-products such as halobenzoquinones and other chlorinated derivatives (Richardson and Postigo 2015; Schmiemann et al. 2023). Such halogenated by-products are often characterized by lower octanol–water partition coefficients, which suggests higher mobility in the aqueous phase and an increased likelihood of bioavailability (Chen et al. 2015a). Additionally, the persistence of these compounds in chloride-rich environments raises significant concerns regarding their contribution to long-term ecological and human health risks (Richardson and Postigo 2015; Zahn et al. 2024). For example, studies have documented that even under conditions where the parent compound has been substantially degraded, residual halogenated by-products such as chlorinated quinoid compounds might persist and induce toxic effects in sensitive aquatic organisms (Gao et al. 2017; Yoom et al. 2018). The complex interplay between halogen concentration, reaction kinetics, and water treatment conditions often leads to a paradoxical situation where measures intended to disinfect or degrade contaminants inadvertently promote the formation of equally hazardous by-products (Richardson and Postigo 2015; Schmiemann et al. 2023).

The ecotoxicological impact of transformation products is a function of their inherent chemical reactivity, bioavailability, and persistence in the environment (Suman et al. 2022). Quinones, due to their potent electrophilic nature, have been shown to cause oxidative stress and disrupt vital cellular processes in aquatic organisms by forming adducts with proteins and nucleic acids (Prasse et al. 2018; Suman et al. 2022). In some instances, the toxicity of a TP exceeds that of its parent compound, thereby precluding the assumption that transformation inherently leads to detoxification (Chen et al. 2015a). Halogenated by-products, particularly those produced in the presence of Cl^–^, can also display adverse effects such as metabolic inhibition, endocrine disruption, and mutagenicity, all of which contribute to adverse ecosystem outcomes (Richardson and Postigo 2015; Schmiemann et al. 2023). Empirical data based on luminescence inhibition assays using *Aliivibrio fischeri* and other nonmammalian model organisms have provided quantitative measures of toxicity, underscoring the potential ecological risks associated with TPs (Suman et al. 2022; Schmiemann et al. 2023). Moreover, the persistence of these transformation products is of particular concern, as their recalcitrant nature limits natural attenuation processes, leading to chronic exposure in aquatic environments over extended time scales (Chen et al. 2015a; Richardson and Postigo 2015; Prasse et al. 2018).

Overall, chloride-rich matrices require careful optimization of oxidant dose, pH, and reaction time because halogen-mediated pathways can increase the number, mobility, and toxicity of transformation products even when parent-pollutant removal is high (Yoom et al. 2018; Xiang et al. 2021). This is particularly evident in studies investigating the transformation of methylparaben, where increasing bromide concentrations (a chemical analog to chloride in reactivity studies) favor the formation of brominated rather than chlorinated methylparabens, with implications for subsequent environmental behavior (Richardson and Postigo 2015; Yoom et al. 2018). In addition, chloride-rich waters often promote a series of secondary reactions that may lead to the formation of multiple halogenated species through sequential substitution and oxidation reactions, thereby complicating the overall chemical profile and risk assessment (Richardson and Postigo 2015; Xiang et al. 2021). The complexity of these reactions necessitates the careful optimization of treatment parameters to minimize the unintended generation of toxic halogenated by-products during routine water treatment processes (Yoom et al. 2018; Xiang et al. 2021).

A critical component in managing the chemical risks posed by transformation products involves the implementation of advanced treatment and remediation strategies that can either prevent TP formation or enhance their subsequent removal from water systems (Villegas et al. 2016; Gao et al. 2019). One promising approach involves the sequential application of tri-metal reduction followed by laccase-catalytic oxidation, a process that has been shown not only to effectively dehalogenate halogenated phenolic compounds, but also to improve the overall biodegradability of the resultant products (Dai et al. 2015). By first reducing the halogenated moieties and then catalytically oxidizing the resulting intermediates, this combined treatment scheme significantly reduces both acute toxicity and the formation of harmful transformation products, thereby addressing the dual challenges posed by quinones and halogenated by-products (Dai et al. 2015). In parallel, chemical oxidation processes using mild oxidants such as permanganate and ferrate have been explored for their ability to degrade phenolic contaminants under controlled conditions, thereby minimizing the formation of toxic intermediates (Villegas et al. 2016; Zahn et al. 2024). These oxidants operate under relatively mild pH and temperature conditions and, when carefully dosed, can achieve substantial reductions in the concentration of both parent compounds and their TPs without the collateral formation of highly toxic chlorinated by-products (Richardson and Postigo 2015; Villegas et al. 2016). Furthermore, electrochemical oxidation techniques have emerged as viable alternatives, providing reagent-free degradation pathways that reduce reliance on chemical additives and thereby potentially lowering the risk of halogenated by-product formation (Villegas et al. 2016; Kotthoff et al. 2019).

**Fig. 4:**
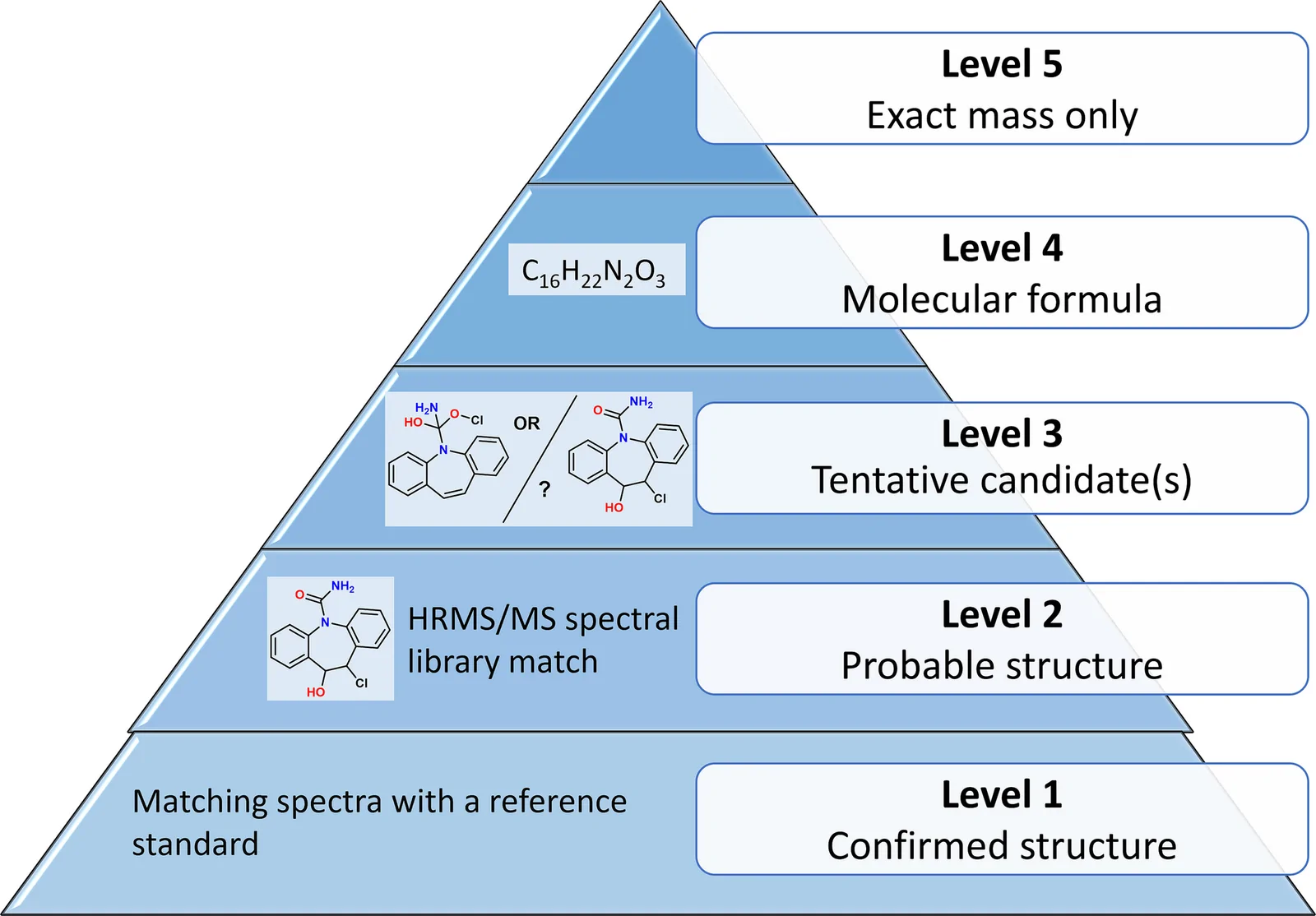
Five-tier Schymanski confidence-level framework for reporting chemical identification certainty in high-resolution mass spectrometry-based nontarget screening. The framework ranges from level 5, where only an exact mass is reported, to level 1, where the structure is confirmed using an authentic reference standard. Abbreviations: HRMS, high-resolution mass spectrometry; MS, mass spectrometry

Over the past decade, non‐target screening using high‐resolution mass spectrometry (HRMS) has revolutionized the identification of unknown compounds in complex environmental and biological matrices. The technique provides an unparalleled level of detail by capturing thousands of signals in a single analysis, making it possible to detect both known and emerging contaminants. However, one of the major challenges in non‐target HRMS is the standardized assessment and communication of identification confidence (Fig. 4). The assignment of confidence levels is critical to evaluating the reliability of identifications and to guiding subsequent confirmatory analyses (Charbonnet et al. 2022; Hollender et al. 2023).

Non‐target screening using HRMS has rapidly evolved due to improvements in instrumentation, data processing algorithms, and chemometric techniques. Its application spans environmental monitoring, metabolomics, and exposomics, where researchers aim to capture a comprehensive chemical profile of a sample without prior knowledge of what compounds are present. The development of robust confidence level frameworks for HRMS identification is essential to this process. One of the most widely recognized frameworks is the Schymanski scale, which categorizes identification confidence from level 1, representing structures confirmed by analytical standards, to level 5, where only the exact mass is known (Hollender et al. 2019, 2023). The non‐target HRMS workflow typically involves several key components: data acquisition, preprocessing of raw spectral data, peak detection and filtering, and compound identification through spectral matching and in silico prediction. The complexity of HRMS data necessitates advanced computational tools and rigorous quality control procedures to handle the vast amounts of information generated during an analysis (Vosough et al. 2024; Boatman et al. 2025). In this context, the development of harmonized protocols and quality assurance criteria is particularly important to ensure that reported confidence levels are both accurate and reproducible (Vitale et al. 2022; Vosough et al. 2024).

Nontarget HRMS identification and reporting of confidence levels are critical components of modern analytical chemistry, particularly in applications related to environmental monitoring and human exposure assessment. The adoption of structured frameworks, such as the Schymanski levels, has greatly facilitated the communication of identification certainty by providing clearly defined categories ranging from confirmed identifications (level 1) to tentative assignments based solely on exact mass (level 5) (Hollender et al. 2019, 2023). In this framework, level 1 corresponds to confirmed identification using an authentic reference standard, whereas level 2 indicates probable structures supported by spectral-library matches or diagnostic fragment evidence. Level 3 denotes tentative candidates, level 4 assigns only a molecular formula, and level 5 reports only an exact mass. This hierarchy is useful for reporting transformation products because it separates confirmed structures from tentative assignments and prevents overinterpretation of HRMS features (Hollender et al. 2019, 2023; Charbonnet et al. 2022; Hulleman et al. 2023).

Despite these advancements, challenges remain in standardizing these practices due to the inherent complexity of HRMS data, variability among instruments, and the limited availability of comprehensive reference standards.

## Spinel-catalyzed Fenton-like degradation of organic pollutants

### Dyes (azo and anthraquinone)

Synthetic dyes remain a priority class of wastewater contaminants because they combine high optical density with structural motifs that resist biological oxidation. Azo dyes constitute the largest commercial group and typically contain one or more azo linkages (–N=N–) connecting aromatic rings. Anthraquinone dyes represent another primary class and feature a fused quinone-like chromophore that stabilizes both the excited and ground states, slowing spontaneous fading and biodegradation. These structural differences translate into distinct oxidative transformation routes and different sensitivities to radical and nonradical pathways in advanced oxidation processes. Spinel ferrites are efficient heterogeneous Fenton and photo-Fenton catalysts for dye removal because they provide redox-active cations and tunable surface chemistry through defect engineering and composite formation (Cheng et al. 2023).

Spinel-catalyzed Fenton-like systems degrade dyes by activating oxidants, most often hydrogen peroxide, to generate reactive oxidizing species. In classical heterogeneous Fenton chemistry, surface Fe^2+^ sites react with H₂O₂ to form hydroxyl radicals. At the same time, Fe^3+^ is reduced back to Fe^2+^ through surface electron transfer, photoreduction, or coupled redox cycling with dopant cations such as Co and Mn. These coupled cycles can increase the steady-state availability of Fe^2+^ and accelerate oxidant turnover. At the same time, the heterogeneous environment modifies the reaction selectivity because adsorption, interfacial charge transfer, and local pH in the catalyst boundary layer can shift the balance between freely diffusing radicals and surface-confined oxidants. These effects are often amplified under irradiation, where ferrites act as visible-light absorbers and where composite semiconductors promote carrier separation and sustain Fe^3+^ reduction (Heidari et al. 2019; Luo et al. 2020).

**Fig. 5:**
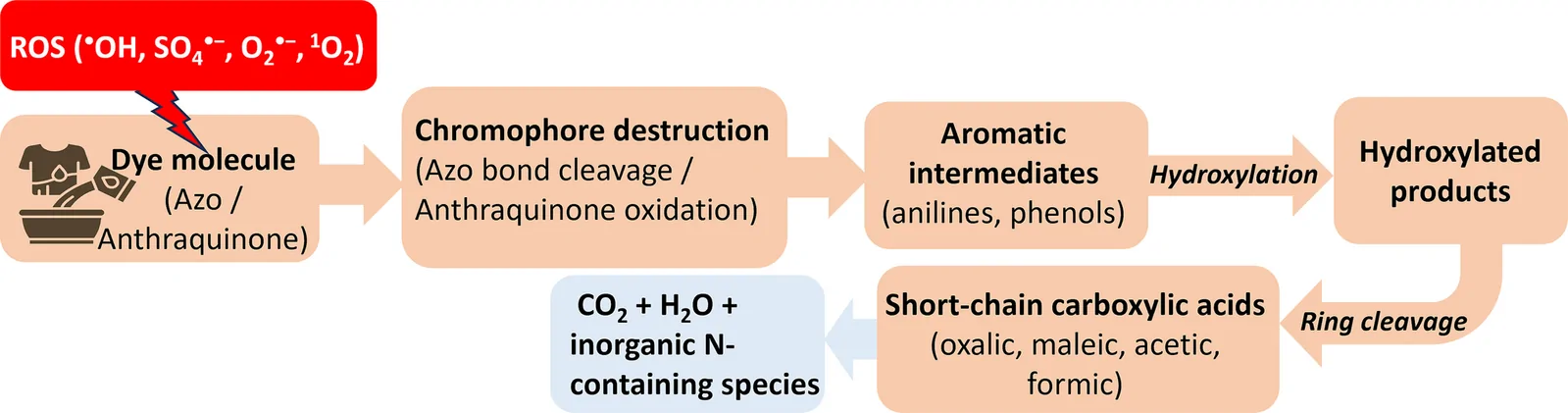
General degradation pathways of azo and anthraquinone dyes during spinel ferrite-mediated Fenton-like oxidation. Reactive oxygen species attack chromophoric structures, leading to azo-bond cleavage or quinone-ring hydroxylation, decolorization, aromatic intermediate formation, ring opening, oxidation to short-chain carboxylic acids, and eventual mineralization

Azo dyes typically undergo initial decolorization through cleavage of the azo bond, followed by oxidation of the resulting aromatic amines and progressive ring-opening to smaller oxygenated products (Fig. 5). In spinel-driven Fenton-like oxidation, the first decisive step is often electrophilic or electron-transfer attack at the azo linkage and adjacent activated aromatic positions. Hydroxyl radicals can add to aromatic rings or abstract hydrogen atoms, while one-electron oxidation can generate dye radical cations that rapidly rearrange and fragment. Once the –N=N– bridge is broken, aromatic amines form as primary intermediates. These amines are environmentally relevant because they may be more toxic than the parent dye, so effective treatment requires continued oxidation beyond color removal. After azo cleavage, hydroxylation and successive oxidation of the aromatic amines produce quinone-like intermediates, short-chain carboxylic acids, and eventually CO₂ under sufficiently strong oxidant flux. Studies on azo dye oxidation in Fenton-type systems consistently show the progression from chromophore destruction to aromatic ring scission and then acid accumulation. The rate-limiting stage often shifts from fast decolorization to slower mineralization because ring-opening requires multiple oxidative equivalents and competes with scavenging reactions in real water matrices. A representative kinetic and mechanistic framework for azo dye oxidation under Fenton conditions has been reported for Acid Orange II, including the importance of controlling oxidant dose to avoid radical scavenging by excess H₂O₂ and to sustain downstream oxidation of aromatic intermediates (Rodríguez et al. 2009).

CoFe₂O₄ spinels can enhance azo dye degradation because Co^2+^/Co^3+^ couples facilitate electron transfer and can promote faster Fe^3+^/Fe^2+^ regeneration at the surface. This improves oxidant activation and can increase the apparent reaction rate at moderate catalyst loadings. CoFe₂O₄ is also a standard building block for hybrid photo-Fenton systems, where irradiation supports the continuous reduction of surface-oxidized states and increases radical yield. For reactive dyes in complex matrices, activated carbon or porous carbon coupling is frequently used to concentrate dye molecules near the catalyst surface and to reduce recombination of photogenerated carriers in photo-Fenton configurations (Heidari et al. 2019). Magnetically recoverable CoFe₂O₄ nanoparticles have also been reported as recyclable Fenton-like catalysts, which supports their use in cyclic operations and continuous treatment concepts (Cao and Zuo 2020).

MnFe₂O₄ provides an additional Mn^2+^/Mn^3+^ redox couple that can cooperate with Fe cycling. In photo-Fenton operation, MnFe₂O₄-based composites can sustain oxidation over a wider pH window. They may shift the dominant oxidant from free radicals to nonradical species such as singlet oxygen, depending on the catalyst’s surface states and oxygen-vacancy content. For dye removal, magnetic MnFe₂O₄/g-C₃N₄ nanocomposites have demonstrated high visible-light photo-Fenton activity for methylene blue, and mechanistic experiments have identified a significant contribution from ^1^O₂ under near-neutral conditions (Angkaew et al. 2021). This behavior is relevant to azo dyes in buffered waters that contain bicarbonate, chloride, or natural organic matter, which can quench ^•^OH and reduce the efficiency of purely radical-driven pathways.

Besides H₂O₂-driven Fenton and photo-Fenton systems, persulfate-based spinel catalysis has also been applied to dye degradation (Manos et al. 2020; Sebah and Belmouden 2025). In these systems, PMS or PDS activation by surface Fe, Co, Mn, or Cu sites can generate SO₄•⁻ together with •OH and, in some cases, ^1^O₂ or surface electron-transfer pathways. Sulfate-radical-driven systems are particularly relevant for dye-contaminated waters because SO₄•⁻ has a high oxidation potential and can promote one-electron oxidation of aromatic chromophores, azo bonds, and auxiliary groups. CoFe₂O₄-based catalysts are especially useful in persulfate activation because Co^2^⁺/Co^3^⁺ cycling can accelerate PMS decomposition and improve interfacial electron transfer, while CuFe₂O₄ and other mixed ferrites provide additional redox pathways through coupled metal cycles. Therefore, PMS/PDS activation should be considered complementary to H₂O₂ activation, especially under near-neutral conditions or in matrices where hydroxyl radicals are strongly scavenged.

Anthraquinone dyes are generally more recalcitrant than many azo dyes because the anthracene-like fused ring system and the quinone carbonyls stabilize the chromophore. As a result, decolorization is less dominated by a single “weak link” such as –N=N–. Instead, oxidation proceeds through successive hydroxylation of the aromatic framework, substitution, and disruption of the quinone system (Fig. 5). A key motif is quinone cycling, where partially oxidized intermediates can undergo redox interconversion and generate reactive oxygen species in situ, especially under irradiation and in the presence of transition metals. This cycling can either accelerate degradation by propagating oxidizing equivalents or slow mineralization if stable semiquinone-like intermediates accumulate.

In Fenton and Fenton-like systems, anthraquinone dyes commonly undergo initial hydroxylation at activated ring positions, followed by cleavage reactions that open the fused ring structure, forming smaller aromatic fragments and organic acids. Because these steps are sequential and require repeated oxidant activation, catalyst design that increases adsorption and improves electron transfer is particularly important for anthraquinone dyes. A broad overview of the persistence of anthraquinone dyes and the challenges associated with their degradation has been discussed in the context of effluent treatment, which highlights why strong oxidative systems are often required for complete removal (Routoula and Patwardhan 2020). Reactive Blue 4 is a widely studied anthraquinone dye and serves as a benchmark substrate for heterogeneous Fenton catalysts. High decolorization has been achieved at acidic pH using immobilized iron species, with limited leaching and practical catalyst recovery. Such studies support the concept that surface-bound iron sites can maintain Fenton activity while mitigating sludge generation compared with homogeneous Fe^2^⁺ dosing (Hassan and Hameed 2011). In spinel ferrite systems, analogous behavior is expected when Fe redox sites are accessible and when mass-transfer limitations to the active surface are minimized.

CuFe₂O₄ spinels combine Fe cycling with Cu^+^/Cu^2+^ redox chemistry. Copper sites can participate in peroxide activation and can facilitate surface electron transfer, which is beneficial when irradiation is used and when the catalyst is designed to suppress charge recombination. A notable strategy is carbon coating or carbon integration, which improves conductivity, increases adsorption, and can promote interfacial charge transfer. Graphitic carbon-coated hollow CuFe₂O₄ spheres have shown strong visible-light photo-Fenton-like activity for methylene blue degradation, attributed to enhanced light harvesting and faster electron transport in the composite architecture (Guo et al. 2017). Related work also reports CuFe₂O₄–Fe₂O₃ composite catalysts for visible-light photo-Fenton degradation of methylene blue, indicating that mixed iron oxide interfaces can further tune reactivity and stability (Silva et al. 2020).

Carbon-coated magnetite (Fe₃O₄@C) is widely used as a reference magnetic Fenton-like catalyst because it combines the redox activity of Fe₃O₄ with a conductive and protective carbon shell. The carbon layer can reduce iron leaching, stabilize the surface against passivation, and enhance the adsorption of cationic dyes such as methylene blue. Fe₃O₄@C nanoparticles have been reported as high-performance Fenton-like catalysts for dye decolorization with H₂O₂, and quenching experiments support hydroxyl radicals as primary oxidants under acidic conditions (Zhang et al. 2014). These findings motivate analogous carbon-integration approaches for CoFe₂O₄, CuFe₂O₄, and MnFe₂O₄ to improve charge transport and stabilize active sites.

Graphitic carbon nitride is frequently coupled with spinel ferrites to form visible-light photoactive heterojunctions. In such hybrids, g-C₃N₄ can supply photogenerated electrons that reduce surface Fe^3+^ to Fe^2+^, thereby sustaining H₂O₂ activation and increasing oxidant yield. At the same time, intimate contact between g-C₃N₄ and the ferrite can create internal electric fields and improve carrier separation. For Fe-doped g-C₃N₄, a heterogeneous photo-Fenton-like system has shown efficient methylene blue degradation across a broad pH range, illustrating the utility of carbon nitride frameworks for dye wastewater treatment and for mitigating matrix effects by controlling the generation of reactive species (Luo et al. 2020). For MnFe₂O₄/g-C₃N₄, mechanistic analysis indicates that ^1^O₂ can play a significant role under near-neutral pH, which is particularly relevant for real textile effluents that contain bicarbonate and other radical scavengers (Angkaew et al. 2021). Beyond dye-specific systems, ZnFe₂O₄–C₃N₄ hybrids provide additional evidence that ferrite–carbon nitride coupling improves photocatalytic performance and catalyst stability, which supports the general design logic of spinel-based photo-Fenton composites (Yao et al. 2014).

For dye remediation, decolorization alone is not an adequate performance metric because aromatic amines, quinone-like intermediates, and small oxygenated by-products can persist. Spinel catalysts can improve mineralization when they sustain oxidant activation over time and when they promote adsorption-driven interfacial reactions that limit radical loss to the bulk matrix. However, practical performance remains sensitive to pH, H₂O₂ dosing, and the presence of scavengers. Under strongly acidic conditions, ^•^OH generation is typically maximized, but the risk of catalyst corrosion and metal leaching can increase for some compositions. Under near-neutral conditions, radical scavenging by bicarbonate and natural organic matter can reduce apparent rates, and nonradical pathways such as ^1^O₂ and surface electron transfer can become comparatively more important. This makes hybrid architectures that stabilize charge transfer and increase surface-confined oxidation valuable for real wastewaters, where selectivity and oxidant utilization efficiency often determine operating cost.

Electro-assisted configurations can also be paired with spinel catalysts to accelerate Fe^3+^ reduction and improve H₂O₂ utilization. For example, cobalt ferrite supported on conductive structures has been applied in electro-Fenton-like systems for dye removal, which demonstrates the broader integration potential of spinels with reactor and power inputs (Dung et al. 2022; Danyliuk et al. 2025). Across these approaches, a consistent design target is to maintain a high local flux of oxidizing equivalents at the dye-adsorbed interface while suppressing unproductive radical termination and minimizing metal release.

In summary, spinel ferrites and their carbon and g-C₃N₄ hybrids provide a versatile platform for dye abatement. Azo dyes often follow the sequence of azo bond cleavage, aromatic amine formation, ring-opening, and mineralization. Anthraquinone dyes proceed through hydroxylation and quinone cycling before chromophore fragmentation. CoFe₂O₄, CuFe₂O₄, and MnFe₂O₄ offer complementary redox features, and carbon coating plus carbon nitride coupling improve adsorption, charge transport, and activity under visible light. These factors collectively support the rational selection of spinel composition and catalyst architecture for efficient and selective Fenton and Fenton-like treatment of dye-containing wastewaters.

### Phenolics and bisphenols

Phenolic compounds constitute one of the most important classes of priority organic contaminants in industrial and municipal wastewaters. This group includes phenol, chlorophenols, nitrophenols, alkylphenols, and endocrine-disrupting bisphenols such as bisphenol A (BPA) and bisphenol S (BPS). Their widespread occurrence originates from petrochemical processing, resin and polymer manufacture, pharmaceuticals, pesticides, coal conversion, and paper production. The environmental concern associated with these compounds extends beyond their acute toxicity because aromatic hydroxyl substituents facilitate the formation of reactive quinones, semiquinones, and other transformation products that may exhibit mutagenic, endocrine-disrupting, or redox-active behavior. Consequently, the treatment of phenolic pollutants requires not only efficient parent compound removal but also extensive mineralization and careful assessment of intermediate formation (Prasse et al. 2018; Gao et al. 2017).

Among heterogeneous advanced oxidation technologies, spinel ferrites and magnetite-based catalysts have emerged as particularly attractive materials for Fenton and Fenton-like degradation of phenolics. Their performance originates from the coexistence of Fe^2^⁺ and Fe^3^⁺ species within the crystal lattice, which continuously activate hydrogen peroxide or persulfate oxidants through surface redox cycling. In Mn-containing ferrites, additional Mn^2^⁺/Mn^3^⁺ transitions further accelerate electron transfer processes and enhance oxidant activation, often expanding catalytic activity to near-neutral pH. The magnetic properties of ferrites also facilitate catalyst recovery and reuse, providing a significant operational advantage over homogeneous Fenton systems that generate large quantities of iron-containing sludge (Xu et al. 2016; Deng et al. 2018).

For phenol degradation, magnetite-based catalysts remain among the most extensively studied systems. In CWPO, surface Fe^2^⁺ sites activate H₂O₂ to generate hydroxyl radicals (•OH), while Fe^3^⁺ species are continuously reduced back to Fe^2^⁺ through lattice-mediated electron transfer. Recent reactor-oriented studies have demonstrated the feasibility of continuous fixed-bed CWPO using natural magnetite, highlighting its low cost, long-term stability, and suitability for scale-up under realistic hydraulic conditions (Lopez-Arago et al. 2024). Catalyst engineering strategies have further improved performance. For example, Al-doped magnetite encapsulated within carbonaceous frameworks exhibited enhanced phenol degradation under mild operating conditions, which was attributed to improved electrical conductivity, reduced particle aggregation, and suppression of catalyst deactivation by strongly adsorbed aromatic intermediates (Thirumoorthy et al. 2021).

**Fig. 6:**
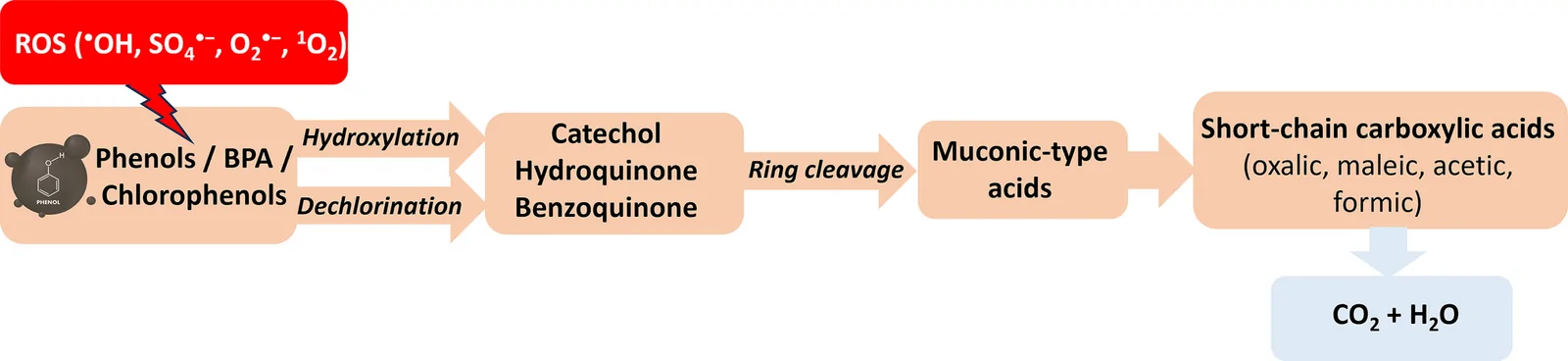
Representative degradation pathways of phenolic pollutants, including phenol, bisphenol A, and chlorophenols, in spinel ferrite-catalyzed Fenton-like systems. Reactive oxygen species (ROS) initiate hydroxylation and, where applicable, dechlorination or C–C bond cleavage, forming catechol, hydroquinone, benzoquinone, and related intermediates. Subsequent aromatic ring cleavage yields muconic-type acids and short-chain carboxylic acids, followed by mineralization to CO₂ and H₂O

The degradation of phenol generally follows a well-established radical oxidation pathway. Hydroxyl radicals initially attack electron-rich positions on the aromatic ring, particularly the para and ortho positions, generating hydroxycyclohexadienyl radicals that rapidly evolve into catechol and hydroquinone (Fig. 6). These intermediates undergo further oxidation to benzoquinone and semiquinone species, followed by ring-opening reactions yielding muconic-type acids and subsequently low-molecular-weight carboxylic acids such as maleic, fumaric, oxalic, formic, and acetic acids. Complete mineralization ultimately produces CO₂ and H₂O when sufficient oxidizing equivalents are available. Because quinones and semiquinones are often more reactive and sometimes more toxic than the parent phenol, monitoring intermediate evolution is essential for assessing treatment effectiveness rather than relying solely on pollutant disappearance (Gao et al. 2017; Prasse et al. 2018).

The efficiency of ferrite catalysts toward substituted phenols has also been widely demonstrated. For example, Fe₃O₄/FeAl₂O₄ composite coatings achieved complete degradation of phenol within 60 min under heterogeneous Fenton-like conditions at pH 4.0, while maintaining excellent structural stability over multiple reuse cycles. The degradation process followed pseudo-first-order kinetics and involved both surface-mediated and homogeneous radical pathways resulting from limited iron leaching (Wang et al. 2016).

Nitrophenols represent another environmentally relevant subgroup because of their persistence and toxicity. Sharma and Singhal (2018) reported that spinel ferrites efficiently catalyze the photo-Fenton degradation of p-nitrophenol under visible irradiation, with CuFe₂O₄ exhibiting the highest activity in H₂O₂-based systems and CoFe₂O₄ showing superior performance for PMS activation. Complete degradation of p-nitrophenol was achieved within minutes under optimized conditions, highlighting the strong influence of the ferrite composition on oxidant activation pathways and radical generation rates.

Chlorophenols present additional mechanistic complexity because chlorine substituents alter electron density, affect reaction kinetics, and promote the formation of chlorinated transformation products and other halogenated by-products. Ferrite-catalyzed oxidation commonly proceeds through concurrent hydroxylation and dechlorination reactions, generating lower chlorophenols, chlorocatechols, chlorohydroquinones, and chlorinated quinones before aromatic ring cleavage. Chloride released during dechlorination may influence radical chemistry and, under certain conditions, promote secondary halogenation reactions. Therefore, oxidant dosage, catalyst surface properties, and solution composition must be carefully controlled to minimize the accumulation of toxic chlorinated intermediates (Dai et al. 2015; Gao et al. 2017). Several ferrite systems have demonstrated remarkable activity toward chlorophenol degradation. NZVI-supported rectorite composites achieved complete removal of p-chlorophenol within 3.5 h through a synergistic mechanism involving adsorption, reductive dechlorination, and Fenton-like oxidation. The catalyst maintained nearly unchanged activity over four successive cycles, demonstrating excellent stability and regeneration potential (Bao et al. 2019). Furthermore, advanced Fe₃O₄-based hybrid catalysts such as Pd@Fe₃O₄@MOF yolk–shell catalysts exhibited exceptionally rapid chlorophenol degradation owing to accelerated Fe(III)/Fe(II) cycling and continuous hydroxyl radical generation, achieving degradation rate constants substantially higher than conventional iron-based catalysts (Niu et al. 2018).

Bisphenols constitute a distinct class of phenolic contaminants because their molecular structures contain two aromatic rings connected by a bridging moiety, which increases hydrophobicity and often enhances adsorption onto carbonaceous catalyst supports. The degradation of BPA and BPS frequently involves both radical-mediated and surface-confined oxidation pathways. In PMS-activated ferrite systems, sulfate radicals (SO₄•⁻), hydroxyl radicals, and surface-bound reactive species can simultaneously participate in degradation. The relative contribution of these pathways depends strongly on catalyst composition, oxidation state distribution, and solution chemistry (Xu et al. 2016; Deng et al. 2018).

Mechanistic investigations of BPA degradation consistently identify hydroxylation, quinone formation, aromatic ring cleavage, and progressive oxidation toward short-chain organic acids as dominant transformation routes. In PMS-activated spinel ferrite systems, MnFe₂O₄, CoFe₂O₄, and CuFe₂O₄ catalysts can promote BPA oxidation through the combined action of sulfate radicals, hydroxyl radicals, and surface-mediated electron-transfer pathways. These reactive routes contribute to aromatic-ring hydroxylation, cleavage of the bisphenol framework, formation of oxygenated intermediates, and subsequent mineralization (Huang et al. 2017a; Li et al. 2021a).

Overall, phenols, chlorophenols, nitrophenols, and bisphenols exhibit a broadly convergent degradation behavior in ferrite-catalyzed Fenton-like systems. Oxidative transformation generally begins with electrophilic radical attack on activated aromatic positions, followed by the formation of catechol-, hydroquinone-, and quinone-type intermediates, aromatic ring cleavage, and eventual mineralization. However, the environmental significance of these processes depends not only on conversion efficiency but also on the suppression of persistent quinoid and halogenated intermediates. Consequently, future studies should increasingly integrate catalyst durability assessments, mineralization measurements, toxicity evolution analyses, and transformation product identification alongside conventional degradation metrics. Such approaches will provide a more realistic evaluation of ferrite-based advanced oxidation technologies for the treatment of phenolic wastewater streams (Gao et al. 2017; Prasse et al. 2018; Zahn et al. 2024).

### Nitroaromatics and anilines

Nitro and amino substituted aromatics, including anilines and nitrophenols, are ubiquitous intermediates and products in dye manufacture, pesticides, pharmaceuticals, and explosives-related supply chains, and they are frequently detected in industrial effluents and contaminated groundwater. Their environmental relevance is linked to high acute toxicity, potential mutagenicity, and the ability of nitro groups to undergo reductive and oxidative interconversions that complicate treatment. Advanced oxidation using heterogeneous iron-containing catalysts and persulfate activation has therefore received sustained attention as a route to rapid abatement with limited secondary waste generation (Zhi et al. 2022).

Magnetic spinel ferrites, such as CoFe₂O₄ and NiFe₂O₄, are widely employed as robust activators of peroxymonosulfate and peroxydisulfate because their mixed-valence metal sites promote interfacial electron transfer and sustain redox cycling during oxidant activation. CoFe₂O₄-containing composites have shown high activity toward aniline removal in peroxymonosulfate-based systems, with mechanistic analyses frequently indicating contributions from sulfate radical and hydroxyl radical chemistry as well as surface-bound reactive intermediates, depending on pH, anions, and catalyst surface states (Zhi et al. 2022; Sheng et al. 2022). NiFe₂O₄-based materials similarly activate persulfates and benefit from magnetic recovery, while compositing with conductive carbons is commonly used to increase the density of accessible sites and to accelerate interfacial electron transport during oxidant turnover (Xie and Xu 2019). In practical applications, the selection between peroxymonosulfate and peroxydisulfate is often guided by matrix effects, since carbonate and natural organic matter can suppress radical availability, whereas surface-mediated pathways may partially compensate when adsorption is significant.

Electro-Fenton schemes provide a complementary route for nitro and amino aromatic removal because hydrogen peroxide can be generated in situ at the cathode and continuously converted to hydroxyl radicals in the presence of iron sites. Fe₃O₄ graphene hybrids and related carbon-supported magnetite electrodes are of interest because the conductive network enhances cathodic oxygen reduction and improves the dispersion of iron active sites, while the magnetic component contributes to structural stability and can facilitate electrode fabrication. Graphene-based electrode concepts are increasingly discussed as a strategy to intensify electro-Fenton performance and reduce energy demand through improved electron transfer and mass transport (Divyapriya and Nidheesh 2020). For aniline in particular, electro-Fenton studies using modified carbon felts and iron-containing cathodes report rapid disappearance of the parent compound and emphasize the importance of controlling current density and dissolved oxygen supply to balance oxidant generation against parasitic reactions (Ou et al. 2019).

**Fig. 7:**
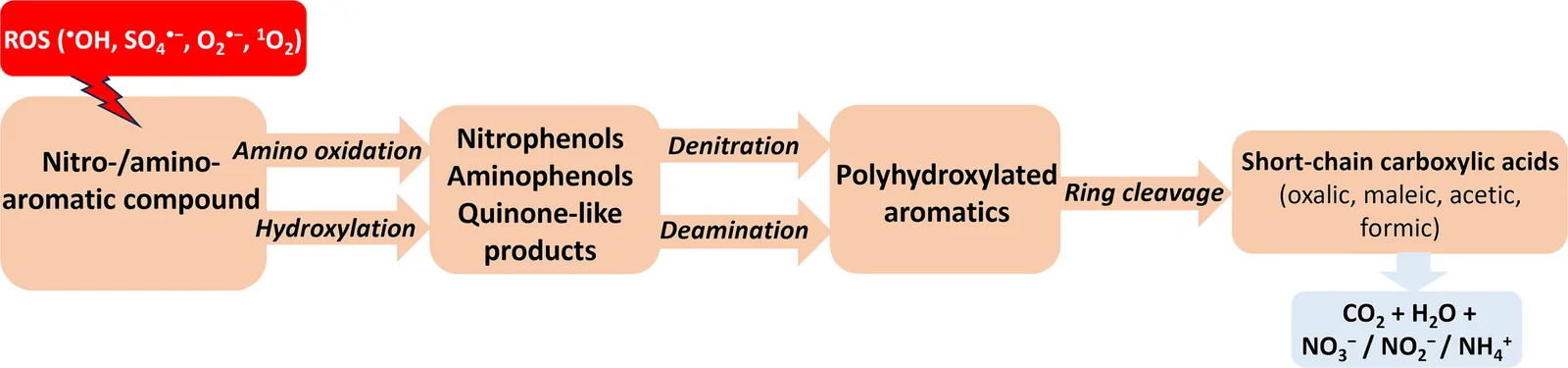
Representative degradation pathway of nitro- and amino-aromatic compounds in spinel ferrite-catalyzed Fenton-like systems. Reactive oxygen species (ROS) initiate hydroxylation and transformation of nitrogen-containing functional groups, generating nitrophenols, aminophenols, quinone-like products, and other hydroxylated intermediates. Subsequent denitration, deamination, and aromatic-ring cleavage produce short-chain carboxylic acids, which are further mineralized to CO₂, H₂O, and inorganic nitrogen species

Across these catalytic platforms, the transformation chemistry of nitro aromatics often follows a characteristic stepwise sequence: the nitro group is reduced to nitroso species, then to hydroxylamine intermediates, and finally to aniline. Subsequent oxidative hydroxylation of the aromatic ring and cleavage reactions yield short-chain acids and mineralization products when sufficient oxidative equivalents are available (Fig. 7). This nitro to aniline sequence is well established in aromatic nitro chemistry and provides a useful conceptual framework for interpreting mixed reduction and oxidation signals in advanced oxidation experiments, especially when both radical and electron transfer processes occur at catalyst surfaces (Corma et al. 2007). In persulfate systems, nitro group conversion can be coupled to aromatic hydroxylation because sulfate radicals can initiate electron transfer reactions while hydroxyl radicals promote electrophilic addition to the ring, leading to polyhydroxylated products that are more susceptible to ring opening.

Nitrophenols are discussed here mainly as nitroaromatic compounds, although their phenolic character also influences adsorption and ring reactivity. In Fenton-like systems, catalyst composition, oxidant dose, and pH control both the degradation rate and the transient accumulation of nitroso-, hydroxylamine-, and aniline-type intermediates before further hydroxylation and aromatic-ring cleavage occur (Van et al. 2023). Photo-Fenton conditions can accelerate these transformations by photoreducing Fe^3^⁺ to Fe^2^⁺ and enhancing hydroxyl radical generation, thereby improving mineralization and limiting the persistence of aromatic intermediates under optimized irradiation and iron availability (Frolova and Khmelenko 2020). Process-intensified approaches, including electromagnetically heated catalysts, have also been explored for refractory nitroaromatics because they can accelerate interfacial kinetics and alleviate mass-transfer limitations (Ribeiro et al. 2019).

Effective treatment of nitro and amino aromatics requires catalyst and process choices that minimize the accumulation of redox-active intermediates such as nitroso- and hydroxylamine derivatives and promote rapid transition to ring hydroxylation and cleavage. Magnetic ferrites activated persulfate systems and electro-Fenton configurations based on magnetite carbon hybrids provide complementary strengths, with the former offering oxidant flexibility and facile separation, and the latter enabling continuous oxidant generation and fine control through electrochemical parameters (Ou et al. 2019; Zhi et al. 2022; Sheng et al. 2022).

### Pesticides (organophosphates, triazines, neonicotinoids)

Pesticides represent a structurally diverse group of micropollutants whose oxidative removal is frequently limited by low concentrations, intense matrix competition, and the formation of persistent transformation products or more mobile intermediates (Lushchak et al. 2018). Spinel ferrites offer a practical platform for pesticide abatement because they combine multivalent redox couples with magnetic recoverability and relatively high resistance to acidic oxidative environments. In applied Fenton-like systems, CoFe₂O₄ and CuFe₂O₄ are most often positioned as heterogeneous activators of H₂O₂ under dark or irradiated conditions, while engineered composites such as CoFe₂O₄/SiO₂ are used to activate persulfate precursors in waters where radical scavenging and selectivity constraints become dominant (Xie and Xu 2019; Jiang et al. 2021). Continuous implementations using natural magnetite-packed beds further emphasize translation potential through sustained activity and simplified catalyst handling (Lopez-Arago et al. 2024).

**Fig. 8:**
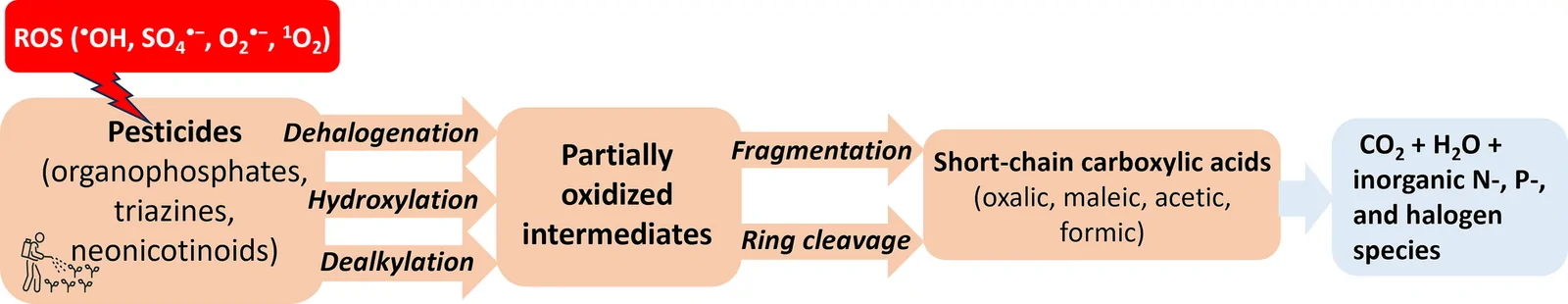
Representative degradation pathway of pesticides, including organophosphates, triazines, and neonicotinoids, in spinel ferrite-catalyzed Fenton-like systems. Reactive oxygen species (ROS) initiate hydroxylation, dealkylation, dehalogenation, and fragmentation reactions, generating partially oxidized intermediates. Subsequent aromatic ring/chain cleavage and fragmentation produce short-chain carboxylic acids, which are further oxidized to CO₂, H₂O, and inorganic nitrogen-, phosphorus-, and halogen-containing species

Although individual pesticide classes differ in their functional groups and primary attack sites, their oxidative degradation in spinel ferrite-catalyzed systems follows several recurring transformation patterns. Organophosphates commonly undergo P–O or P–S bond cleavage and subsequent oxidation of phenolic or heteroaromatic fragments; triazines are typically transformed through dealkylation, dechlorination, and hydroxylation; and neonicotinoids often undergo oxidation of nitroimino or cyanoimino moieties, heterocycle cleavage, and formation of chlorinated nicotinic acid derivatives. These class-specific reactions converge toward partially oxidized intermediates, followed by fragmentation, ring opening, short-chain carboxylic acid formation, and, under sufficient oxidant exposure, mineralization. A generalized pathway summarizing these common pesticide-transformation routes is shown in Fig. 8.

#### Organophosphates (OPs)

Organophosphate insecticides such as chlorpyrifos and diazinon contain electrophilic P centers and hydrolysable P–O or P–S bonds, and their oxidative transformation often begins with bond cleavage that yields more polar phenolic products and phosphate species. In chlorpyrifos oxidation, the formation of 3,5,6-trichloro-2-pyridinol (TCP) is widely recognized as a key primary product that can persist and require subsequent oxidative steps for detoxification and mineralization (Zhang et al. 2018). Practical Fenton-like treatments therefore target rapid parent removal while maintaining sufficient oxidant exposure to drive TCP conversion, which is typically slower than initial P–O scission.

A representative persulfate route is provided by mesoporous CoFe₂O₄/SiO₂ composites, in which the silica framework promotes dispersion and creates a confined microenvironment that enhances oxidant–substrate contact. Such systems have been demonstrated for chlorpyrifos degradation via activated persulfate and are often paired with physical intensification methods to mitigate external mass-transfer limitations (Xie and Xu 2019). Mechanistically, persulfate activation on Co sites supports sequential oxidation beyond the initial cleavage step, which is essential because OP transformation products can retain toxicity or exhibit higher mobility than the parent compound.

CuFe₂O₄-based materials are increasingly employed in photocatalytic or photo-assisted configurations to overcome limitations of dark H₂O₂ activation. A Z-scheme CuFe₂O₄/MIL-101(Fe) composite has been reported for chlorpyrifos degradation under simulated sunlight, with identified pathways that include initial bond cleavage and progressive oxidation of aromatic fragments, accompanied by toxicity evaluation of intermediates (Tian et al. 2023). While this approach is not limited to classical H₂O₂-driven Fenton chemistry, it is relevant to ferrite-enabled pesticide treatment because it leverages ferrite redox cycling, surface adsorption, and light-driven charge separation to broaden operating windows and reduce dependence on highly acidic conditions.

For diazinon and related OPs, product distributions reported in electro-Fenton studies provide a useful mechanistic benchmark for ferrite-catalyzed systems because the same functional group liabilities govern initial oxidative attacks. Diazinon degradation commonly proceeds through oxidation of thiono groups, followed by fragmentation, producing phenolic or heteroaromatic alcohols and phosphate-containing products, which then undergo further oxidation to carboxylates (Heidari et al. 2020). In heterogeneous ferrite systems, these same steps are expected to be modulated by adsorption affinity, diffusion into catalyst pores, and the local oxidant flux near the surface.

#### Triazines (atrazine, simazine, related herbicides)

Triazine herbicides are among the most studied pesticide families in oxidative treatment because they are persistent, widely detected, and generate characteristic dealkylated and hydroxylated intermediates. Atrazine transformation typically begins with dealkylation to deisopropylatrazine and deethylatrazine, followed by dechlorination and hydroxylation to hydroxyatrazine and further ring-substituted products. Under UV/H₂O₂, hydroxyatrazine is formed and subsequently converted to ammeline, with reported toxicity trends for the transformation products (Choi et al. 2013). These well-defined sequences are valuable for interpreting ferrite-catalyzed systems because they distinguish between partial detoxification and deeper ring destruction.

Spinel ferrite activation of sulfate oxidants is particularly effective for atrazine, and CuFe₂O₄ has been reported to activate peroxymonosulfate (PMS), leading to rapid atrazine degradation, with performance attributed to the catalytic interaction of the ferrite surface with PMS and to the magnetic separability of the catalyst (Guan et al. 2013). CoFe₂O₄-based PMS systems likewise exhibit strong atrazine reactivity and are frequently cited as attractive options when complex matrices reduce hydroxyl-radical efficiency (Li et al. 2018). In practice, this distinction matters because triazines are often encountered in natural waters with bicarbonate and dissolved organic matter, which can suppress radical pathways and shift the process toward less efficient oxidation routes.

For H₂O₂-driven treatment, CoFe₂O₄ can be integrated into photo-Fenton-like architectures that combine adsorption and catalytic oxidation to address both low influent concentrations and limited contact time. A notable example is CoFe₂O₄ deposited on activated carbon fibers, which promotes atrazine removal through combined uptake and photo-Fenton degradation, improving performance through higher local pollutant concentration at catalytic sites and enhanced recyclability due to magnetic behavior (Jiang et al. 2021). This hybrid logic is particularly relevant to translation because triazine removal in continuous flow often benefits from a capture-and-oxidize strategy rather than relying solely on homogeneous radical exposure.

#### Neonicotinoids (imidacloprid, thiamethoxam, acetamiprid)

Neonicotinoids contain electron-deficient heterocycles and nitroimino- or cyanoimino moieties that yield distinctive oxidative and reductive pathways. Transformation commonly involves N-oxide formation or nitro-group rearrangements, followed by heterocycle cleavage to generate chlorinated nicotinic acid derivatives, such as 6-chloronicotinic acid, for chloropyridinyl neonicotinoids. Detailed kinetic and mechanistic work on the direct reactions of PMS and PDS with neonicotinoids has mapped dominant pathways and highlighted the formation of stable carboxylated products that can persist under conditions that are not sufficiently strong or sustained for oxidative degradation (Yuan et al. 2024).

Spinel-assisted photo-Fenton approaches have been explored for acetamiprid and related targets where selective oxidation and by-product control are essential. A graphene oxide composite decorated with magnetite and cobalt ferrite has been used for UV-accelerated pesticide degradation, illustrating how ferrite-containing hybrids can couple adsorption, photochemistry, and catalytic oxidant activation to accelerate neonicotinoid transformation while retaining recoverability (Tabasum et al. 2020a). Complementary studies with ferrite catalysts in water treatment contexts also report acetamiprid removal trends that depend strongly on irradiation and oxidant availability, supporting the need for integrated process control rather than catalyst selection alone (Tabasum et al. 2020b).

**Fig. 9:**
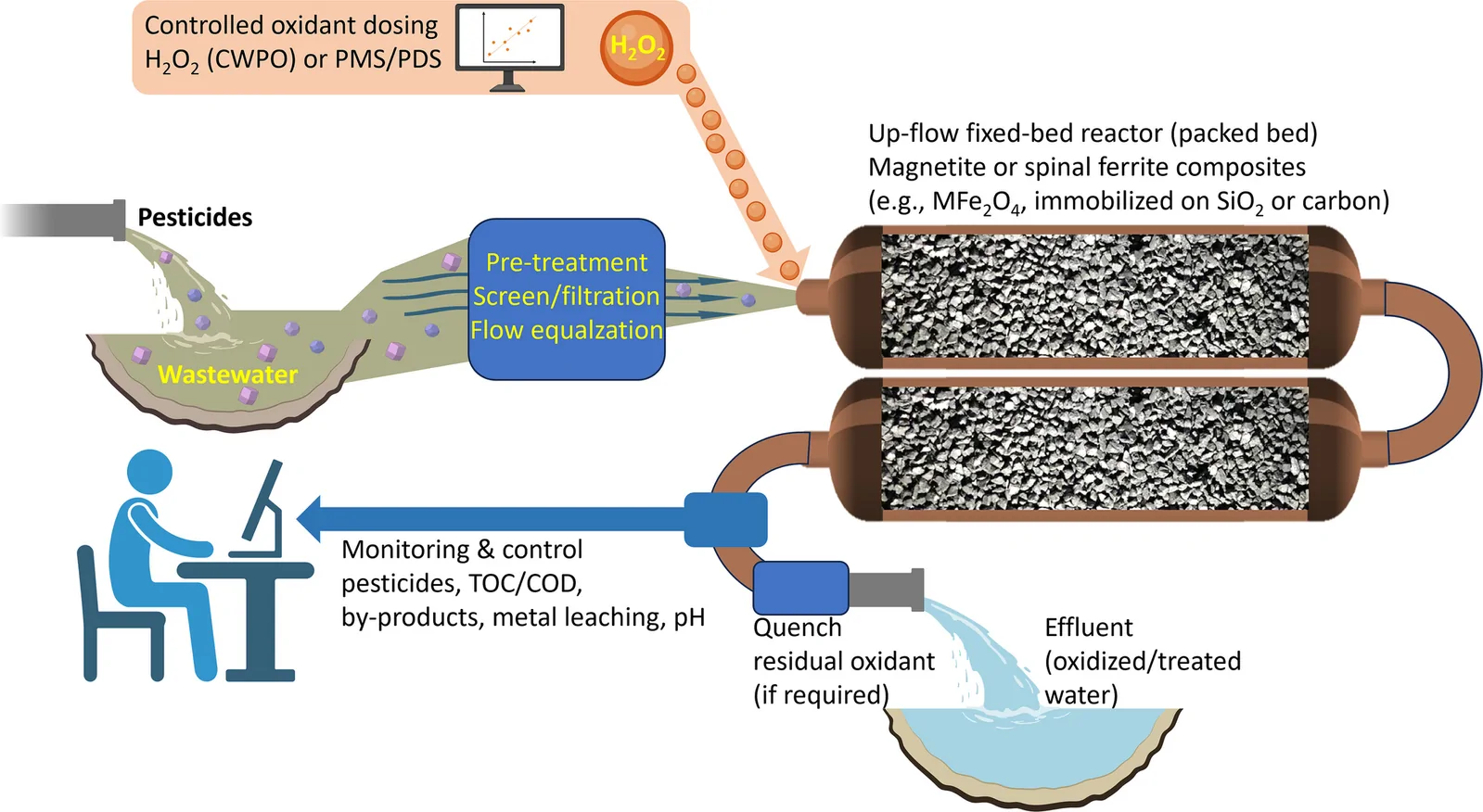
Proposed continuous-flow fixed-bed catalytic oxidation system for pesticide removal using controlled oxidant dosing and iron-oxide or spinel ferrite catalysts. After pre-treatment by screening, filtration, and flow equalization, water enters an up-flow fixed-bed reactor packed with magnetite or supported spinel ferrite catalysts, such as CoFe₂O₄ or CuFe₂O₄ on SiO₂ or carbon. H₂O₂, PMS, or PDS is dosed in a controlled manner to sustain catalytic oxidation while avoiding overdosing and catalyst passivation. Process performance is monitored through pesticide removal, TOC/COD, by-product formation, metal leaching, and pH, and the effluent may be quenched or neutralized to remove residual oxidant before discharge

For neonicotinoids, the persistence of oxidized intermediates can be a practical constraint, particularly when partial oxidation increases polarity and mobility. This motivates reactor strategies that maintain a controlled oxidant residual across the contact time and promote repeated interaction with catalytic surfaces. Persulfate activation can be advantageous because it allows sustained oxidant delivery, and mechanistic work indicates that both direct oxidant reactions and secondary reactive species can contribute depending on conditions (Yuan et al. 2024). In ferrite-based systems, these insights translate to operational choices such as stepwise dosing, fixed-bed residence time, and the use of structured supports that minimize diffusion limitations (Fig. 9).

#### Continuous operation and stability considerations

Translation to continuous treatment emphasizes catalyst lifetime, hydraulic stability, and a manageable pressure drop (Fig. 9). Natural magnetite packed in an up-flow fixed-bed reactor has been demonstrated as a long-lasting catalyst for catalytic wet peroxide oxidation of pesticide mixtures, with sustained operation and evaluation in realistic effluent matrices (Lopez-Arago et al. 2024; Tatarchuk et al. 2024). While that study emphasizes azole pesticides, it provides an instructive precedent for pesticide-class treatment in fixed-bed mode using iron-oxide minerals, and it highlights design priorities that are also applicable to OPs, triazines, and neonicotinoids. These priorities include minimizing catalyst attrition, maintaining bed wetting, and avoiding excessive instantaneous oxidant levels that accelerate nonproductive consumption and surface passivation.

For spinel ferrites, the stability of Co and Cu sites under oxidative cycling remains a key issue, especially in acidic solutions and in the presence of chelating organic intermediates. Practical mitigation approaches include immobilization on porous supports, protective or conductive coatings, and coupling to adsorption phases that reduce the required oxidant exposure for a given conversion target (Fig. 9). The demonstrated use of CoFe₂O₄/SiO₂ for chlorpyrifos and CoFe₂O₄ on carbon fibers for atrazine reflects this direction, because both architectures improve dispersion and reduce aggregation while enabling straightforward separation (Xie and Xu 2019; Jiang et al. 2021).

Spinel ferrite catalysts enable multiple viable routes for pesticide degradation, but the optimal pathway depends strongly on pesticide class. Organophosphates often undergo rapid P–O or P–S cleavage to phenolic and phosphate products, and ferrite-activated persulfate systems provide a practical route to push oxidation beyond persistent intermediates such as TCP (Zhang et al. 2018; Xie and Xu 2019). Triazines exhibit characteristic dealkylation and dechlorination sequences, and both CuFe₂O₄-activated PMS and CoFe₂O₄-based photo-Fenton hybrids have demonstrated strong atrazine performance, especially when adsorption and oxidation are integrated (Guan et al. 2013; Jiang et al. 2021). Neonicotinoids can yield stable carboxylated chloronicotinic derivatives and other persistent products, which underscores the need for sustained oxidant delivery and reactor strategies that maintain repeated catalytic contact (Yuan et al. 2024). Finally, continuous fixed-bed demonstrations with natural magnetite indicate that mineral-based catalytic peroxide oxidation can be stable and scalable, and similar reactor concepts are directly relevant for introducing spinel-ferrite pesticide treatment into practical flow systems (Lopez-Arago et al. 2024).

### Pharmaceuticals and endocrine-disrupting compounds

Pharmaceuticals and endocrine-disrupting compounds are frequently detected in surface waters and treated effluents because conventional biological treatment is often ineffective for structurally complex and biologically active molecules. Antibiotics, antiepileptics, anti-inflammatory drugs, and steroidal hormones are of particular concern due to persistence, mixture effects, and the potential to promote antibiotic resistance or endocrine-mediated impacts at low concentrations. Spinel ferrites and magnetite-based catalysts have therefore been increasingly applied in heterogeneous Fenton-like and persulfate-based systems, where catalytic redox cycling enables strong oxidant activation while facilitating catalyst recovery.

Photo-assisted Fenton-like oxidation using ferrite–semiconductor hybrids is particularly attractive for antibiotics such as tetracycline, as visible-light excitation can enhance iron-site regeneration and sustain reactive oxygen species production under milder chemical conditions. NiFe₂O₄/g-C₃N₄ composites have been reported to accelerate tetracycline transformation under irradiation through heterojunction-mediated charge separation coupled with Fenton-like pathways, leading to faster removal and improved stability relative to the individual components (Liu et al. 2022). Related g-C₃N₄/NiFe₂O₄ heterojunction designs, including S-scheme configurations, have also demonstrated efficient tetracycline hydrochloride degradation under visible light, supporting the broader conclusion that interfacial charge transfer control is a key lever for intensifying photo-Fenton performance in ferrite-based systems (Mishra et al. 2024). In these systems, illumination increases the steady-state formation of oxidizing species and accelerates surface metal-site cycling, thereby increasing the likelihood that partially oxidized intermediates undergo further transformation rather than desorb and persist.

Persulfate activation by mixed-metal ferrites offers a complementary route for pharmaceuticals and endocrine disruptors, particularly when operation near neutral pH is desired. Co- and Mn-containing ferrites are widely studied activators of peroxymonosulfate and peroxydisulfate because their redox couples can support rapid electron transfer to the oxidant and generate sulfate-radical-driven and hydroxyl-radical-driven oxidation, with the relative contribution depending on matrix composition and catalyst surface states. A representative example of light-assisted persulfate activation is MnFe₂O₄/g-C₃N₄, which has been applied as a visible-light PMS activator for hormone-contaminated water, illustrating how ferrite chemistry and semiconductor photophysics can be integrated to widen the practical operating window while maintaining magnetic recoverability (Poomipuen et al. 2023). Broader assessments of cobalt ferrite and related ferrite families likewise emphasize their usefulness for oxidizing refractory pharmaceuticals through persulfate activation, while highlighting the importance of controlling metal leaching and maintaining long-term surface reactivity (Raveendran et al. 2026).

Electro-Fenton platforms based on magnetite immobilization on conductive cathodes provide an additional intensification pathway, particularly for persistent neutral pharmaceuticals such as carbamazepine and for widely used anti-inflammatory drugs such as ibuprofen. In electro-Fenton, H₂O₂ is generated in situ by cathodic oxygen reduction and is converted to hydroxyl radicals at iron sites, enabling continuous and tunable oxidizing flux without bulk dosing of soluble iron. Magnetite nanoparticles fixed on carbon fiber textile cathodes have been demonstrated to support carbamazepine degradation and mineralization with low iron loading and improved sustainability compared with conventional homogeneous electro-Fenton concepts (Liu et al. 2018). In parallel, slurry-type magnetite Fenton-like systems have shown that carbamazepine and ibuprofen can be degraded at near-neutral pH, while also clarifying the strong influence of suspended solids and clay minerals on observed kinetics and oxidant utilization (Sun et al. 2013).

**Fig. 10:**
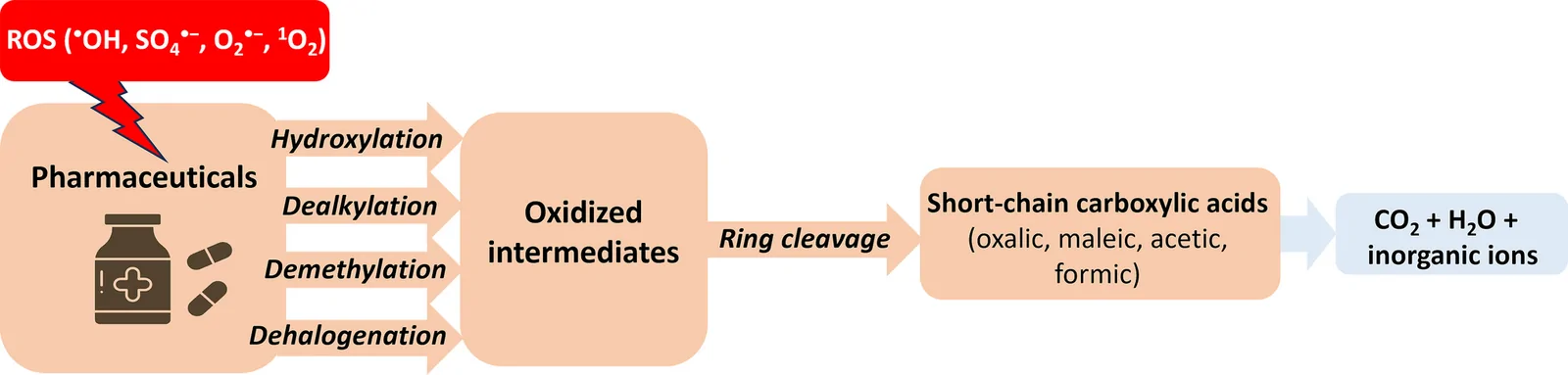
Representative degradation pathway of pharmaceuticals in spinel ferrite-catalyzed Fenton-like systems. Reactive oxygen species (ROS), including hydroxyl radicals (•OH), sulfate radicals (SO₄•⁻), and singlet oxygen (^1^O₂), initiate hydroxylation, dealkylation, demethylation, dehalogenation, and side-chain cleavage reactions, producing oxidized intermediates. Subsequent aromatic and/or heterocyclic ring cleavage generates short-chain carboxylic acids, which are further oxidized to CO₂, H₂O, and inorganic ions

Despite differences in oxidant and reactor configuration, the dominant transformation logic for pharmaceuticals in these ferrite-driven systems is broadly consistent. Initial steps commonly include hydroxylation of aromatic and olefinic moieties, dealkylation of tertiary amines or ether side groups, and scission of labile side chains, followed by formation of smaller oxygenated intermediates that evolve toward carboxylic acids and mineralization when sufficient oxidative equivalents are supplied (Fig. 10). For tetracyclines, hydroxylation and ring modifications typically occur alongside cleavage of functionalized side groups, producing progressively more oxidized fragments that can enter short-chain acid pools. For carbamazepine, oxidation often begins with electrophilic attack and epoxidation-like transformations on the aromatic framework and the alkene region, followed by ring opening and conversion to low-molecular-weight acids under sustained treatment. Hormonal endocrine disruptors and related steroidal compounds often exhibit a phenolic or activated aromatic ring that undergoes rapid oxidative transformation, producing quinone-type intermediates and subsequent cleavage products. Heterogeneous ferrite catalysts have been applied to steroidal targets via combined adsorption and oxidative transformation, highlighting that adsorption can increase interfacial exposure and promote deeper conversion for hydrophobic endocrine-active molecules (Hu et al. 2011). For all these classes, a recurring operational challenge is preventing the accumulation of partially oxidized intermediates that may retain bioactivity, which makes mineralization metrics and toxicity-informed assessment important complements to parent-compound removal.

The most effective ferrite-based strategy for pharmaceuticals and endocrine disruptors is to couple strong interfacial reactivity with process control that sustains oxidation beyond the first generation of hydroxylated and dealkylated intermediates. NiFe₂O₄/g-C₃N₄ photo-Fenton designs are particularly promising for antibiotics because visible-light assistance can improve oxidant efficiency and reduce kinetic stalling, while Co- and Mn-containing ferrites provide flexible persulfate activation routes that can operate under broader water-chemistry conditions. Magnetite-based electro-Fenton is most compelling when continuous operation and precise oxidant delivery are needed, provided that the reactor is run to maintain a sufficiently high oxidative flux to drive ring cleavage and suppress persistence of bioactive transformation products.

### Polycyclic aromatic hydrocarbons and other polycyclic species

Polycyclic aromatic hydrocarbons (PAHs) and other polycyclic species are among the most persistent organic contaminants because fused aromatic rings provide high resonance stabilization, low polarity, and limited aqueous solubility. These properties drive strong partitioning into particulate matter and carbonaceous phases, which impose mass-transfer constraints and can decouple intrinsic catalytic activity from the apparent removal rate in water and sediment systems. For spinel-catalyzed Fenton-like processes, PAHs therefore represent a class where integrated adsorption and oxidation are especially valuable, since preconcentration at the catalyst interface can increase local substrate availability while limiting nonproductive radical losses in the bulk solution (Usman et al. 2012; Faheem et al. 2020).

Spinel–carbon composites operationalize this capture-and-oxidize concept by combining hydrophobic uptake sites with redox-active spinel domains that activate H₂O₂ or sulfate oxidants. Carbon supports improve dispersion and electron transport, while also enriching PAHs near reactive surface sites, which can be decisive under diffusion-limited conditions. In persulfate systems, magnetic iron oxide and carbon hybrids have been used to oxidize PAHs in contaminated sediments at circumneutral pH, and product toxicity has been explicitly evaluated, underscoring that partial oxidation can yield transformation products that remain biologically active and therefore require sustained oxidative treatment beyond parent compound depletion (Dong et al. 2019a). Recent composite activators built from biochar and ferrite phases also show that adsorption contributes to rapid initial uptake, while persulfate activation sustains deeper conversion, with magnetic recovery enabling reuse in repeated cycles (Gu et al. 2023; Chen et al. 2024a).

Photo-assisted architectures extend this strategy by accelerating interfacial redox cycling and enhancing reactive oxygen species formation under visible irradiation. g-C₃N₄/spinel photo-Fenton hybrids are designed to improve charge separation and promote regeneration of reduced metal sites that drive continuous oxidant activation. In such systems, the semiconductor component supports the photoreduction of surface Fe(III) and related redox couples, thereby increasing the steady-state oxidizing flux and promoting oxidation beyond oxygenated intermediates. This logic is consistent with broader photo-Fenton developments for iron-containing composites, where improved carrier management translates into higher oxidant utilization and better stability across wider pH conditions than classical homogeneous Fenton operation (Clarizia et al. 2017; Pastor et al. 2022).

Visible-light-assisted oxidant activation can also be strengthened through heterostructure design, in which a visible-light-responsive component is coupled with a redox-active ferrite phase. Such coupling is useful because improved charge-carrier separation and interfacial electron transfer can accelerate surface redox cycling, increase oxidant utilization, and support catalyst recovery. For example, BiFeO₃/MnFe₂O₄ ferrite nanocomposites have been reported to exhibit enhanced magnetic and electrical properties, indicating that coupling perovskite-type light absorbers with spinel ferrites can improve functional properties relevant to heterogeneous photo-Fenton catalyst design (Remya et al. 2020). More broadly, composite and heterojunction approaches align with the general trend in heterogeneous advanced oxidation toward surface-confined reactivity, in which pollutant adsorption, oxidant activation, and electron transfer are co-localized to reduce scavenging losses and improve robustness in complex matrices (Thomas et al. 2021; Yan et al. 2023).

**Fig. 11:**
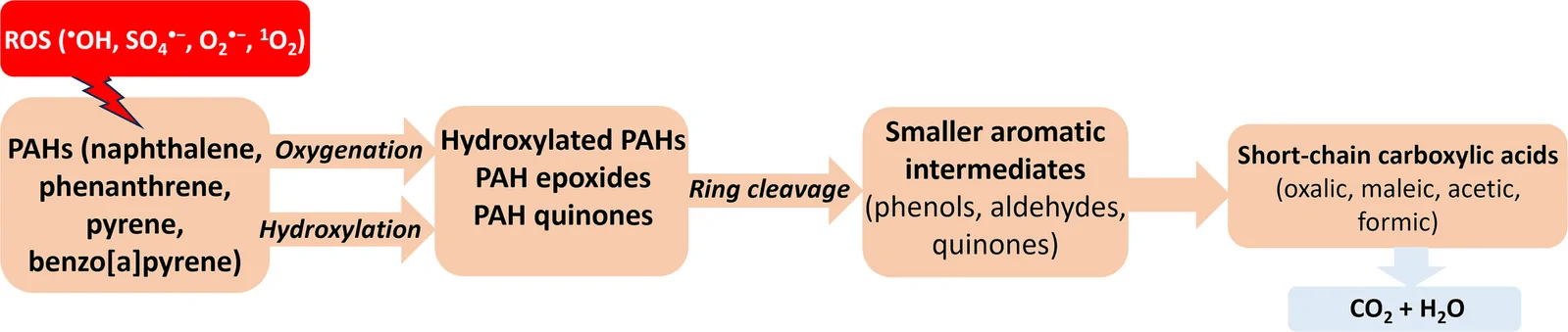
Representative degradation pathway of polycyclic aromatic hydrocarbons (PAHs) in spinel ferrite-catalyzed Fenton-like systems. Reactive oxygen species (ROS) initiate hydroxylation, oxygenation, or epoxidation of fused aromatic rings, producing hydroxylated PAHs, quinone-like compounds, and other oxygenated intermediates. Subsequent aromatic-ring cleavage generates smaller aromatic products and short-chain carboxylic acids, which are further oxidized to CO₂ and H₂O

PAH transformation in radical-based oxidation typically proceeds through initial hydroxylation or epoxidation of the aromatic rings, producing oxygenated intermediates that evolve to quinones and related oxy-PAHs, followed by progressive ring cleavage to smaller oxygenated fragments and, under sufficient oxidative equivalents, short-chain acids and mineralization (Fig. 11). Quinone formation is a recurring mechanistic node because these intermediates are redox-active and can persist when oxidant flux is insufficient or when mass-transfer limits repeated catalytic contact. In heterogeneous ferrite systems, the same sequence is expected to be modulated by adsorption affinity, diffusion into pores, and the local oxidant concentration in the catalyst boundary layer, which together determine whether treatment achieves detoxification or stalls at oxygenated polycyclic intermediates (Usman et al. 2012).

The main conclusion of this subsection is that successful PAH abatement with spinel-driven Fenton-like chemistry depends on integrating hydrophobic adsorption with controlled, surface-proximal oxidant activation, because this pairing directly addresses both low solubility and intrinsic aromatic stability. Spinel–carbon composites provide a practical route to increase local availability and sustain conversion, while photo-assisted and visible-light heterostructures offer additional leverage by enhancing redox cycling and oxidant utilization. For deployment-oriented designs, the decisive performance criterion is the ability to drive oxidation beyond quinone-type intermediates toward irreversible ring opening, while maintaining catalyst durability and minimizing the persistence of toxic oxygenated by-products.

### Surfactants and alkylphenol ethoxylates

Surfactants constitute a challenging class of wastewater contaminants because amphiphilicity promotes micellization, interfacial adsorption, and strong interactions with suspended solids and natural organic matter. These features can decrease the apparent efficiency of advanced oxidation by sequestering the target within aggregates and by increasing radical scavenging in complex matrices. Alkylphenol ethoxylates, particularly nonylphenol ethoxylates (NPEOs), present an additional concern because oxidative or biological shortening of the ethoxylate chain can generate nonylphenol and short-chain NP1–2EO species that are more hydrophobic and often more endocrine-active than the parent mixture. Consequently, treatment concepts increasingly aim to couple rapid primary conversion with reliable progression toward deep oxidation and mineralization rather than partial transformation (Song et al. 2023).

Catalytic wet peroxide oxidation with magnetite and cobalt ferrite spinels is attractive for surfactant-containing streams because heterogeneous catalysts can be separated and reused, while surface redox cycling sustains H₂O₂ activation and hydroxyl radical generation. Magnetite-based CWPO has been widely used as a deployment-oriented platform, and magnetite-based catalysts have been evaluated in different aqueous matrices, supporting their relevance for practical wastewater treatment (Huaccallo-Aguilar et al. 2021). Carbon-supported iron catalysts further show how adsorption at the catalyst interface can intensify reactivity by localizing organic molecules and intermediates near the radical-generation zone (Rey et al. 2016). CoFe₂O₄-based systems provide additional redox flexibility, and cobalt ferrite encapsulated in a graphitic shell has shown enhanced performance in catalytic wet peroxide oxidation, illustrating how carbon coupling can improve interfacial reactivity and catalyst handling (Ribeiro et al. 2019). For alkylphenol ethoxylates specifically, biochar–CoFe₂O₄/PMS has been reported for nonylphenol ethoxylate 10 degradation, transformation-product identification, and mechanistic analysis, supporting the use of spinel–carbon composites that combine adsorption with catalytic oxidant activation (Song et al. 2023). For practical reactor operation, foaming is a major constraint when oxidizing surfactants, since gas evolution and surface-active residuals can destabilize hydraulics and reduce effective contact. Therefore, fixed-bed or flow-through configurations based on spinel–carbon composites may be advantageous because hydrophobic uptake and distributed oxidation sites can reduce free surfactant concentrations in the bulk phase and smooth oxidant delivery under continuous operation.

Electro-Fenton offers a complementary intensification route that is particularly relevant for streams in which chemical dosing is undesirable or continuous operation is required. In electro-Fenton, H₂O₂ is generated in situ at the cathode and converted to hydroxyl radicals in the presence of iron, while applied current provides tunable control over oxidant flux. Spinel-coated cathodes and spinel–carbon electrodes are increasingly explored to combine oxygen reduction activity, iron availability, and mechanical stability in a single architecture, and they can reduce catalyst loss relative to slurry systems (Brillas et al. 2009). Electrochemical Fenton treatment of nonylphenol polyethoxylates has demonstrated effective removal and provides mechanistic insight into how electrochemical operation can support both chain shortening and oxidation of the aromatic moiety when sufficient oxidative equivalents are maintained (Martins et al. 2006). At the same time, micelles can slow oxidation by steric shielding and restricted mass transfer, which has been explicitly demonstrated for nonylphenol ethoxylates during electro-oxidation and is directly relevant to electro-Fenton and CWPO designs that depend on radical attack in the aqueous phase (Ji et al. 2022).

**Fig. 12:**
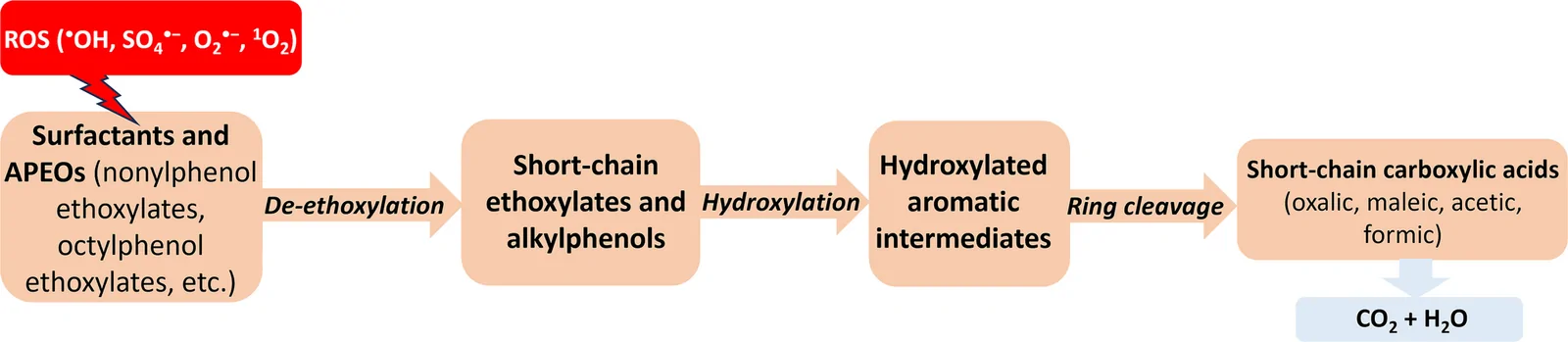
Representative degradation pathway of surfactants and alkylphenol ethoxylates (APEOs) in spinel ferrite-catalyzed Fenton-like systems. Reactive oxygen species (ROS) initiate oxidation of ethoxylate chains, alkyl chains, and aromatic moieties, producing shorter-chain ethoxylates, alkylphenols, and other oxidized intermediates. Subsequent aromatic-ring and/or aliphatic-chain cleavage generates short-chain carboxylic acids, which are further oxidized to CO₂ and H₂O

The dominant degradation routes for conventional surfactants typically involve progressive chain shortening initiated at the terminal carbon atoms of alkyl chains or near heteroatom linkages, yielding aldehydes and carboxylic acids that can be further oxidized toward carbon dioxide and water (Fig. 12). For NPEOs, a characteristic sequence begins with cleavage of ethoxy units and scission of ether bonds, producing a distribution of shorter ethoxylates and, under extensive de-ethoxylation, nonylphenol and NP1–2EO species. Subsequent hydroxylation of the aromatic ring and oxidation to quinone-type intermediates can occur, followed by ring opening and formation of low-molecular-weight acids. The kinetic bottleneck is often the transition from partial oxidation products, including short-chain ethoxylates and alkylphenolic intermediates, to irreversible fragmentation, which requires sustained radical availability and sufficient contact between hydrophobic intermediates and the catalyst surface. Laboratory studies of Fenton oxidation of NPEOs have documented both the appearance of shorter ethoxylates and the importance of controlling operating conditions to limit accumulation of persistent intermediates, supporting the need for process designs that are robust against micellization and matrix scavengers (Cui et al. 2014).

From an application perspective, the most effective strategy for surfactants and alkylphenol ethoxylates is to design systems that combine interfacial capture with controlled oxidant activation, because this directly addresses micelle shielding, foaming, and the risk of accumulating endocrine-active short-chain products. CWPO with magnetite or CoFe₂O₄ is best suited when simple, recoverable catalysts and straightforward reactor layouts are prioritized, while flow-bed spinel–carbon configurations are advantageous when foam control and stable hydraulics are critical. Electro-Fenton with immobilized spinel-containing cathodes is most valuable when continuous operation and tunable oxidant generation are needed, but it must be operated to maintain a high enough oxidative flux to push reactions beyond ethoxylate cleavage and alkylphenol formation toward ring opening and mineralization.

## Outlook, benchmarking, and research needs

The rapid growth of spinel ferrite research has produced a remarkably diverse landscape of catalyst compositions, architectures, oxidants, and target pollutants, making it difficult to navigate without an organizing framework. Table 1 offers a critical assessment of the principal spinel ferrite families, covering their dominant reactive oxygen species, key strengths, limitations, and remaining development needs.

**Table 1:** Critical comparison of spinel ferrite catalysts for Fenton-like oxidation of organic pollutants

Spinel ferrite	Main advantages	Main limitations	Dominant ROS/mechanism	Typical applications	Future research needs
CuFe₂O₄	High redox activity due to Cu⁺/Cu^2^⁺ and Fe^2^⁺/Fe^3^⁺ cycling; often high degradation rate	Potential Cu leaching; stability concerns under acidic or strongly oxidizing conditions	•OH-dominated in H₂O₂ systems; SO₄•⁻ possible in PMS/PDS systems	Dyes, pesticides, pharmaceuticals	Long-term stability, leaching control, and immobilized operation
CoFe₂O₄	Excellent PMS activation; efficient sulfate radical generation; broad pH applicability	Potential Co release; toxicity and regulatory concerns	SO₄•⁻, •OH, and possible nonradical pathways	PMS-based degradation of dyes and emerging contaminants	Development of low-leaching, cobalt-efficient, and immobilized systems
MnFe₂O₄	Magnetic recovery; lower toxicity than CoFe₂O₄ and CuFe₂O₄ in many systems; relatively stable	Lower catalytic activity than CuFe₂O₄ and CoFe₂O₄ in many systems	•OH and SO₄•⁻	Pharmaceuticals, phenolic compounds	Enhancement of electron transfer, active-site density, and catalyst durability
ZnFe₂O₄	Narrow band gap; visible-light response; suitable for photo-Fenton and photocatalytic systems	Low intrinsic redox activity; fast charge recombination; often requires heterojunction or carbon coupling	Photo-generated ROS and •OH	Photocatalytic degradation of dyes and antibiotics	Charge-separation engineering, heterojunction design, and defect control
NiFe₂O₄	Good chemical stability and magnetic properties	Moderate catalytic activity; slower kinetics in some systems	•OH and photo-generated ROS	Dye degradation and wastewater treatment	Surface modification, conductivity enhancement, and defect engineering
Mixed-metal ferrites (Cu–Mn, Co–Mn, etc.)	Synergistic redox effects; enhanced electron mobility and oxidant activation	More complex synthesis; possible multi-metal leaching	Mixed radical and nonradical pathways	Broad-spectrum pollutant removal	Rational design based on structure–activity–stability relationships
Carbon-modified ferrites (rGO, biochar, CQDs, CNTs)	Improved conductivity, adsorption, charge transfer, catalyst stability, and nanoparticle dispersion	Additional synthesis complexity and cost; long-term carbon stability remains insufficiently studied	Enhanced ROS generation and electron-transfer pathways	Emerging contaminants and real wastewater	Scale-up, long-term durability, regeneration, and leaching assessment

Table 2 complements this comparison with representative degradation data across organic pollutant classes, summarizing removal efficiencies, reaction kinetics, catalyst stability, and reusability where reported. Together, the two tables point to two recurring themes: catalytic activity depends strongly on both composition and catalyst architecture, and comparison across studies remains complicated by differences in oxidants, operating conditions, water matrices, and performance metrics. More standardized benchmarking is therefore essential for translating laboratory activity into practical catalyst selection.

**Table 2:** Representative performance of spinel ferrite catalysts for Fenton-like degradation and catalic reduction of organic pollutants. Removal efficiency, mineralization, rate constants, and reusability are summarized where reported in the original studies. Abbreviations: *PMS*, peroxymonosulfate; *PDS*, peroxydisulfate; *TOC*, total organic carbon; *k*, apparent reaction rate constant

Pollutant	Pollutant dose	Catalyst dose	Oxidant/process	pH	Temperature (°C)	Time (min)	Removal (%)	*k*/kobs (min⁻^1^)	Reuse cycles	Ref
Dyes										
Rhodamine B	2 × 10⁻^5^ mol L^−1^	CuFe₂O₄, 0.44 g L^−1^	83 mmol L^−1^ H₂O₂/UV photocatalysis	6.2	22	200	95 discoloration (10 min); 97 degradation (200 min)	nr	nr	Flores et al. 2013
	10 mg L^−1^	Cu₀.₅Mg₀.₅Fe₂O₄–TiO₂, 1.0 g L^−1^	Visible-light photocatalysis	nr	25	180	98.4	0.01396	nr	Tran et al. 2021
		0.4 g L^−1^ Ag/Fe₂O₃ MCs-2%	30% H₂O₂/heterogeneous photo-Fenton		nr	120	nr	nr	5	Chen et al. 2016b
	2 × 10⁻^5^ mol L^−1^	ZnFe_2_O_4_–TiO_2_, 1 g L^−1^	UV photocatalysis			150	99.7	0.00971		Zhang et al. 2010
Remazol Red RR	40 mg L^−1^	CoFe₂O₄, 1.0 g L^−1^	200 mM H₂O₂/dark Fenton-like catalysis	7.2	22	1440	96.5	Behnajady model; apparent k not reported	3	Cechinel et al. 2023
Congo red	100 mg L^−1^	NiFe₂O₄ nanoparticles, 1 g L^−1^	2 mM H₂O₂/visible-light photocatalysis	5.0	25	60	96.8	0.0853	nr	Hariani et al. 2021
Methylene blue	30 mg L^−1^	NiFe₂O₄ nanoparticles, 0.4 g/L	MEDL microwave-assisted photocatalysis	nr	nr	10	88.6	0.21059	4	Wang et al. 2021b
	10 mg L^−1^	Ag_3_PO_4_/NiFe_2_O_4_ Composites = APO/NFO_5_, 10 mg/run	Visible-light photocatalysis			10–30	95.0	0.532	3	Patil et al. 2015
	10^−5^ M	Ni₀.₆Co₀.₄Fe₂O₄, 10 mg/run				180	100	0.0169	nr	Kahhki et al. 2017
Malachite green	10^−5^ M	Zn_0.5_Ni_0.5_Fe_2_O_4_ modified by Eu(III) and Tb(III), 1 g L^−1^		5.8–6.5		150	64	0.00677		Tsvetkov et al. 2019
Acid Orange 7	10 mg L^−1^	CoFe₂O₄–rGO, 0.08 g L^−1^	3 mM H₂O₂/ultrasound sono-Fenton catalysis	3.0		120	90.5	Langmuir–Hinshelwood (L–H) kinetic model	5	Hassani et al. 2018
	20 mg L^−1^	CuFe₂O₄–SG, 0.6 g L^−1^	0.8 g L^−1^ persulfate/persulfate activation	6.5		30	96.8	nr	3	Xian et al. 2020
		CuFe₂O₄, 0.2 g L^−1^				90	87.6	0.0198	nr	
		CoFe₂O₄, 0.2 g L^−1^					~ 50	0.0061		
		MnFe₂O₄, 0.2 g L^−1^					~ 36–37	0.0059		
		ZnFe₂O₄, 0.2 g L^−1^					~ 20	0.0009		
Phenolics										
Phenol	1 mmol L^−1^	Fe-Lap-TA-350, 1 g L^−1^	50 mmol L^−1^ H₂O₂/heterogeneous photo-Fenton catalysis	3.0	30	5	Almost complete conversion, ~ 100	*k*₁ = 0.0062; *k*₂ = 0.0157	nr	Iurascu et al. 2009
	35 mg L^−1^	Fe₃O₄/FeAl₂O₄ composite coating, 6 cm^2^ in 50 mL solution	6.0 mmol L^−1^ H₂O₂/heterogeneous Fenton-like system	4.0		60	100	*k*₁ = 0.016; *k*₂ = 0.137	4	Wang et al. 2016
	50 mg L^−1^	Yolk/shell Pd@Fe₃O₄@MOFs, 0.5 g L^−1^	1.0 mM H₂O₂/heterogeneous Fenton-like, dark		nr	10	100	0.512	5	Niu et al. 2018
	200 mg L⁻^1^	100 mg Fe(Fe₀.₆₈Al₀.₃₂)₂O₄@MC in fixed-bed reactor	0.036 mol H₂O₂/CWPO, heterogeneous Fenton, fixed-bed continuous reactor	5.0	40	30	~ 80	0.14	nr	Thirumoorthy et al. 2021
2-Chlorophenol	50 mg L^−1^	Yolk/shell Pd@Fe₃O₄@MOFs, 0.5 g L^−1^	1.0 mM H₂O₂/heterogeneous Fenton-like, dark	4.0	nr	120	100	0.036	5	Niu et al. 2018
4-Chlorophenol						10		0.586		
2,4-Dichlorophenol								0.480		
2,4,6-Trichlorophenol						120	75	0.007		
Nitro and amino aromatics and anilines										
4-Nitroaniline	0.1 mmol L^−1^	MnFe₂O₄, 1 g L^−1^	0.5 M NaBH₄ reduction	9.6–9.95	25	4.5	100	0.813	nr	Ibrahim et al. 2016
2,4,6-Trinitrophenol								0.680		
4-Nitrophenol								0.637	5	
	20 mg L^−1^	S_0.2_-CF@C, 0.14 g L^−1^	0.4 g L^−1^ PMS activation	6.4		30	99	0.126		Zhu et al. 2022
p-Nitrophenol	0.21 mM	CuFe₂O₄, 0.50 g L^−1^	8.8 mM H₂O₂/photo-Fenton, visible light	2.5	nr	10	90	0.210	4	Sharma and Singhal 2018
		CuFe₂O₄, 0.50 g L^−1^	2.2 mM PMS/photo-Fenton, visible light	Natural		40	96	0.076		
Pesticides										
Atrazine	10 mg L^−1^	CoFe₂O₄, 0.4 g L^−1^	0.8 mM PMS	6.3	30	30	> 99	0.181	5	Li et al. 2018
	9.6 mg L^−1^	Fe^3^⁺ = 0.4 mM	Fenton-like; H₂O₂ = 75 mM	3.0	25	300	nr	0.033	nr	Chen et al. 2016a
Ametryn			Fenton-like; H₂O₂ = 70 mM			200		0.034		
Prometon			Fenton-like; H₂O₂ = 60 mM					0.045		
2,4-D	5 mg L^−1^	Fe⁰@Fe₃O₄, 0.5 g L^−1^	1 mM H₂O₂/heterogeneous Fenton-like	5.0	30	90	100	0.0458–0.365		Lv et al. 2020
	10 mg L^−1^	FeS, 0.5 g L^−1^	FeS/10 mM H₂O₂, heterogeneous Fenton-like	4.5		300	70.4	nr		Chen et al. 2015b
Dimethoate		Fe^3^⁺ = 1 mg L^−1^	40 mg L^−1^ H₂O₂/UV–Fe^3^⁺–H₂O₂, photo-Fenton	2.9–3.0	30–35	15	100	0.268		Evgenidou et al. 2007
Methyl parathion						50		0.086		
Glyphosate	0.1 mM	Fe(II)(aq), 0.1 mM Fe^2^⁺	Homogeneous electro-Fenton	3.0	25	40	81.65	0.063		Tran et al. 2019
	1.0 mg L^−1^	Fe^3^⁺/C₂O₄^2^⁻ = 20/300 μmol L^−1^	Ferrioxalate photodegradation	3.0	22	180	63.2	nr		Chen et al. 2007
	5.0 mg L^−1^			3.5			nr			
	0.59 mmol L^−1^	0.27 mmol L^−1^ Fe^2^⁺/Fe^3^⁺ + 1.13 mmol L^−1^ oxalate	10.3 mmol L^−1^ H₂O₂/photo-Fenton	2.8	40	120	75	nr		Souza et al. 2013
	10 mg L^−1^	0.2 g L^−1^ goethite + 0.5 mM oxalate	Goethite/oxalate/visible light Fenton-like photoreaction	3.5	25	180	39.7	nr		Zhang et al. 2019
	1 × 10⁻^4^ mol L^−1^	0.4 g L^−1^ Ag/Fe₂O₃ MCs-2%	30% H₂O₂/heterogeneous photo-Fenton	nr	nr	120	50	nr		Chen et al. 2016b
Clothianidin	10 mg L^−1^	MoSx/ceramic membrane, MoSx loading = 5.3 mg cm^−2^; Fe^2^⁺ = 0.04 mM	MoSx/CM/Fe^2^⁺/H₂O₂ Fenton-like flow-through process; H₂O₂ = 0.4 mM; ^1^O₂-dominated oxidation	3.0		8	nr		3	Yi et al. 2022
Imidacloprid		FePO₄/WB, 250 mg L^−1^	Heterogeneous Fenton-like; PMS = 0.5 mM; Fe(IV)-dominated oxidation	6.78	25	50	34.09	0.0453	8	Zhao et al. 2024
Thiacloprid							nr	0.079	nr	
Nitenpyram								0.060		
Dinotefuran								0.039		
Clothianidin								0.034		
Pharmaceuticals										
Sulfamethoxazole	10 mg L^−1^	CoFe₂O₄, 0.1 g L^−1^	0.4 g L^−1^ PMS	9.0	nr	10	91	nr	nr	Li et al. 2021b
	5.0 mg L^−1^	MgFe₂O₄/MIL-88A (MF140M60), 0.4 g L^−1^	2.94 mM H₂O₂/visible light photo-Fenton catalysis	5.8		30	99.8 within 20 min; 100 within 40 min	0.1533	5	Shi et al. 2022
Ofloxacin	20 μM	CoFe₂O₄, 0.10 g L^−1^	0.4 mM PAA, 0.6 mM H₂O₂	7.0	25	45	83.0	0.066	nr	Li et al. 2023
Ibuprofen	10.0 mg L^−1^	MgFe₂O₄ nanoparticles, 0.5 g L^−1^	20.0 mM H₂O₂ heterogeneous Fenton catalysis	8.0	20	40	98–100	0.091		Ivanets et al. 2020
Paracetamol	10 mg L^−1^	CoFe₂O₄, 0.2 g L^−1^	0.20 mM PMS	4.3	25	60	90.5	0.0534	3	Tan et al. 2017
	10 mg L^−1^	MnFe₂O₄, 0.2 g L^−1^					100	0.121		
Polycyclic aromatic hydrocarbons										
Fluoranthene	80 µg L^−1^	Fe^2^⁺ = 3.1 × 10⁻^4^ M	3.1 × 10⁻^3^ M H₂O₂/Fenton-like catalysis	3.0	nr	180	nr	*k*₁ = 0.303; *k*₂ = 0.006	nr	Flotron et al. 2005
Benzo[a]pyrene								*k*₁ = 0.539; *k*₂ = 0.058		
Phenanthrene	nr, sand slurry	Goethite/α-FeOOH = 33.5 g/kg	5 M H₂O₂/Fenton-like catalysis	Neutral			73	2.0 × 10⁻^4^–1.1 × 10⁻^3^		Kanel et al. 2004
Anthracene							60	1.5 × 10⁻^4^–9.0 × 10⁻^4^		
Pyrene							55	1.1 × 10⁻^4^–7.0 × 10⁻^4^		
Phenanthrene	94.2 mg kg^−1^	nZVI-CAC = 10 g kg^−1^; Fe/CAC = 1:1	100 mmol kg^−1^ persulfate activation	8.0		nr	60.3	0.00179		Chen et al. 2024b
Naphthalene	10 mg L^−1^	Fe₃O₄/GO/g-C₃N₄, 40 mg	Visible-light photocatalysis	5–9		140	87.56	0.0137	5	Ali et al. 2022
Surfactants and alkylphenol ethoxylates										
Nonylphenol ethoxylates	3.25 × 10⁻^5^ M	Fe^2^⁺ = 3.25 mM	9.74 mM H₂O₂/Fenton-like catalysis	3.0	25	6	84	0.8628	nr	Cui et al. 2014
Nonylphenol ethoxylate-9	300 mg L^−1^	Fe^2^⁺ = 0.048 mM	0.48 mM H₂O₂/UV-photo-Fenton-like catalysis	2.8		180	92.6	0.042		de la Fuente et al. 2010
Linear alkylbenzene sulfonate	200 mg L^−1^	Fe^2^⁺ = 120 mg L^−1^	800 mg L^−1^ H₂O₂/photo-Fenton-like catalysis	3.0	nr	80	86.66	nr		Miranzadeh et al. 2016
Sodium dodecyl sulfate	2 mg L^−1^	Fe^2^⁺ = 5.5 mg L^−1^	100 mg L^−1^ H₂O₂/solar photo-Fenton-like catalysis	Acidic		15	> 99			Esteban García et al. 2020

*nr* not reported

*PMS* peroxymonosulfate

Removal (%) may mean discoloration, parent-compound degradation, concentration removal, or TOC/mineralization, as reported in the cited study; the wording in each cell follows the reported metric where available

For graph-only values, the rate constants were taken from the plotted/fitted values in the cited paper; exact removal percentages were not added when only visually estimated from graphs

Recent progress makes one broader trend clear: spinel-catalyzed Fenton-like oxidation is moving beyond laboratory-scale slurry experiments toward engineered continuous processes. Two scale-up directions stand out. One is the emergence of pilot-oriented persulfate activation systems built on mixed-metal spinels that sustain high PMS activation rates while remaining compatible with scalable synthesis (Huang et al. 2022; Guo et al. 2022). The other is the development of low-cost mineral-based catalysts with natural magnetite in fixed-bed CWPO systems being the clearest example, which has shown long-term stability and minimal metal leaching under continuous-flow conditions (Lopez-Arago et al. 2024). These are not competing approaches. They represent complementary deployment strategies: natural magnetite prioritizes simplicity and economic viability, while engineered spinels offer higher activity, tunable selectivity, and compatibility with PMS- and PDS-based oxidation chemistries.

Despite this momentum, several challenges continue to slow large-scale implementation. One of the most pressing is mechanistic: under realistic water-treatment conditions, the relative importance of radical versus nonradical oxidation pathways is still poorly understood. Many spinel and spinel–carbon systems operate through a mixed pool of reactive species, and the apparent mechanism can shift substantially in the presence of bicarbonate, chloride, natural organic matter, and other matrix constituents that alter radical lifetimes. Resolving this requires more than quenching experiments alone. Future studies should bring together selective quenching, operando spectroscopies, isotopic methods, and electrochemical approaches to distinguish between hydroxyl radical, sulfate radical, singlet oxygen, and surface electron-transfer pathways, and to do so in representative water matrices rather than idealized laboratory solutions (Ren et al. 2021; Zhu et al. 2025).

Standardizing how TPs and toxicity are assessed is an equally important priority. A recurring finding in this area is that removing the parent pollutant does not necessarily mean detoxifying the water. Aromatic amines, quinones, oxygenated intermediates, and halogenated by-products can persist even when conversion efficiencies appear high. Future studies should therefore move beyond simple removal metrics and routinely combine targeted and nontarget high-resolution mass spectrometry, explicit confidence levels for TP identification, mineralization and oxidant-utilization data, and ecotoxicological screening to give a fuller picture of what treatment actually achieves (Menacherry et al. 2022).

Catalyst durability and materials sustainability deserve equal attention. Spinel ferrites offer real advantages in terms of magnetic recoverability and compositional flexibility, but long-term stability under realistic operating conditions has not been demonstrated convincingly in most studies. Future work should routinely track phase evolution, defect restructuring, metal release, and activity retention over extended cycling, and should use leaching-adjusted controls to separate genuine heterogeneous catalysis from the contributions of dissolved metal species (Tian et al. 2022). Material sustainability also needs to enter catalyst design thinking more explicitly, particularly for cobalt-containing ferrites, where environmental and supply-chain concerns are substantial and replacement strategies that preserve catalytic performance are needed.

Reactor and process engineering represent another frontier that has received insufficient attention. Catalyst rankings obtained in batch experiments do not reliably predict continuous-flow performance, because mass transfer, oxidant dosing, hydraulic residence time, and reactor hydrodynamics become the dominant variables during scale-up. Future benchmarking should therefore converge on a limited set of representative reactor configurations, including fixed-bed systems, monolithic reactors, membrane-assisted systems, and conductive flow-through electrodes for electro-Fenton applications, and evaluate them using harmonized metrics: space–time yield, oxidant utilization efficiency, energy consumption, pressure drop, catalyst lifetime, and sustained operating duration (Keen et al. 2018; Huang et al. 2022).

The evidence summarized in Tables 1 and 2 supports one clear conclusion: no single spinel ferrite catalyst is universally optimal. Catalyst selection will always need to be guided by the target pollutant class, oxidant chemistry, water matrix, reactor configuration, and operational constraints specific to the application. The most significant near-term advances are likely to come not from discovering a new champion catalyst, but from integrating catalyst design, mechanistic understanding, toxicity evaluation, and reactor engineering within unified benchmarking frameworks. Evaluating performance not just by pollutant conversion, but by detoxification efficiency, operational stability, resource consumption, and long-term environmental impact, is what will ultimately move spinel ferrite catalysts from laboratory demonstrations to technologies that remain effective under real-world operating conditions.

## Conclusion

Spinel ferrite-catalyzed Fenton and Fenton-like oxidation constitutes a versatile platform for degrading persistent organic pollutants, because it combines tunable redox chemistry with magnetic recoverability and compatibility with multiple oxidants, including hydrogen peroxide, peroxymonosulfate, and peroxydisulfate.

Reaction selectivity is governed by the balance between radical and nonradical pathways. Hydroxyl and sulfate radical regimes often dominate under conditions that support rapid Fe redox cycling, whereas near neutral pH and complex matrices containing bicarbonate, chloride, or natural organic matter can shift reactivity toward singlet oxygen formation or surface-mediated electron transfer. This shift directly changes transformation product profiles and can increase the risk of persistent or toxic intermediates if mineralization is not ensured.

Catalyst design advances are converging on iron-rich spinels of the general formula MFe_2_O_4_, in which defect engineering, lattice symmetry modulation, and dopant selection regulate Fe^2+^ to Fe^3+^ turnover and oxygen vacancy density, thereby controlling oxidant activation efficiency. Composite architectures with carbon supports or semiconductor junctions enhance adsorption, electron transport, and photo-assisted operation under visible light, thereby sustaining activity in realistic water chemistries.

Durability remains a deployment-limiting factor. Reliable application requires leaching resolved benchmarking, operando tracking of phase and surface evolution, and mitigation strategies such as immobilization, protective shells, and conductive coatings, while catalyst compositions should increasingly reflect abundance and regulatory acceptability, including the need to replace cobalt without sacrificing performance.

Reactor translation is increasingly shifting from slurry demonstrations to continuous configurations. Fixed beds, monoliths, and adsorption oxidation composites can simplify separation, improve hydraulic stability, and enable controlled oxidant dosing that reduces mass-transfer limitations and suppresses halogenated by-product formation in chloride-rich waters.

For safe and scalable implementation, the field should prioritize standardized transformation product identification with explicit confidence levels, routine toxicity tracking alongside parent removal and mineralization metrics, harmonized reactor figures of merit for batch-to-flow comparisons, and pilot-scale validation under variable influent chemistries.

## Data Availability

No new data were created or analyzed in this study. Data sharing is not applicable to this article.

## Abbreviations

AOPs: Advanced oxidation processes
ACs: Fe-loaded activated carbons
COD: Chemical oxygen demand
CWPO: Catalytic wet peroxide oxidation
DOM: Dissolved organic matter
ETR: Surface electron transfer route
Fe₃O₄@C: Carbon-coated magnetite
Δ*G*: Gibbs free energy change
g-C_3_N_4_: Graphitic carbon nitride
h^+^: Hole
kLa: Volumetric gas–liquid mass-transfer coefficient
MFe₂O₄: Spinel ferrite with a divalent metal cation M
M–O: Metal oxygen bond
MS: Mass spectrometry
HRMS: High‐resolution mass spectrometry
NOM: Natural organic matter
Δ*P*: Pressure drop
PAHs: Polycyclic aromatic hydrocarbons
PDS: Peroxydisulfate
PMS: Peroxymonosulfate
p–n: P-type to n-type semiconductor junction
ROS: Reactive oxygen species
SHE: Standard hydrogen electrode
TCP: 3,5,6-Trichloro-2-pyridinol
TOC: Total organic carbon
TPs: Transformation products
UV: Ultraviolet irradiation

## References

[CR1] Ahmadian-Fard-Fini S, Salavati-Niasari M, Safardoust-Hojaghan H (2017) Hydrothermal green synthesis and photocatalytic activity of magnetic CoFe_2_O_4_–carbon quantum dots nanocomposite by turmeric precursor. J Mater Sci Mater Electron 28:16205–16214. 10.1007/s10854-017-7522-1

[CR2] Ahn Y, Choi J, Kim M, Kim M, Lee D, Bang W, Yun E, Lee H, Lee J, Lee C, Maeng S, Hong S, Lee J (2021) Chloride-mediated enhancement in heat-induced activation of peroxymonosulfate: new reaction pathways for oxidizing radical production. Environ Sci Technol 55:5382–5392. 10.1021/acs.est.0c07964 33733765 10.1021/acs.est.0c07964

[CR3] Ali A, Raza T, Ahmed A, Ali MS, Liu C, Li D, Li C (2022) Facile synthesis of magnetically separable Fe_3_O_4_/GO/g-C_3_N_4_ composite for superior photocatalytic degradation of naphthalene. Synth Met 287:117072. 10.1016/j.synthmet.2022.117072

[CR4] Angkaew A, Chokejaroenrat C, Sakulthaew C, Mao J, Watcharatharapong T, Watcharenwong A, Imman S, Suriyachai N, Kreetachat T (2021) Two facile synthesis routes for magnetic recoverable MnFe_2_O_4_/g-C_3_N_4_ nanocomposites to enhance visible light photo-Fenton activity for methylene blue degradation. J Environ Chem Eng 9(4):105621. 10.1016/j.jece.2021.105621

[CR5] Audino F, Campanyà G, Pérez-Moya M, Espuña A, Graells M (2019) Systematic optimization approach for the efficient management of the photo-Fenton treatment process. Sci Total Environ 646:902–913. 10.1016/j.scitotenv.2018.07.057 30067960 10.1016/j.scitotenv.2018.07.057

[CR6] Badreldin A, Abusrafa AE, Abdel‐Wahab A (2020) Oxygen‐deficient cobalt‐based oxides for electrocatalytic water splitting. Chemsuschem 14(1):10–32. 10.1002/cssc.202002002 33053253 10.1002/cssc.202002002 PMC7839495

[CR7] Bao T, Jin J, Damtie MM, Wu K, Yu ZM, Wang L, Chen J, Zhang Y, Frost RL (2019) Green synthesis and application of nanoscale zero-valent iron/rectorite composite material for P-chlorophenol degradation via heterogeneous Fenton reaction. J Saudi Chem Soc 23(7):864–878. 10.1016/j.jscs.2019.02.001

[CR8] Boatman A, Chappel J, Kirkwood-Donelson K, Fleming J, Reif D, Schymanski E, Rager J, Baker E (2025) Updated guidance for communicating PFAS identification confidence with ion mobility spectrometry. Environ Sci Technol 59(33):17711–17721. 10.1021/acs.est.5c01354 40808630 10.1021/acs.est.5c01354 PMC12424050

[CR9] Brienza M, Katsoyiannis I (2017) Sulfate radical technologies as tertiary treatment for the removal of emerging contaminants from wastewater. Sustainability 9(9):1604. 10.3390/su9091604

[CR10] Brillas E, Sirés I, Oturan M (2009) Electro-Fenton process and related electrochemical technologies based on Fenton’s reaction chemistry. Chem Rev 109:6570–6631. 10.1021/cr900136g 19839579 10.1021/cr900136g

[CR11] Buwa VV, Roy S, Ranade VV (2016) Three-phase slurry reactors. Multiphase Catalytic Reactors 132–155:132. 10.1002/9781119248491.ch6

[CR12] Cao Z, Zuo C (2020) Direct synthesis of magnetic CoFe_2_O_4_ nanoparticles as recyclable photo-Fenton catalysts for removing organic dyes. ACS Omega 5:22614–22620. 10.1021/acsomega.0c03404 32923821 10.1021/acsomega.0c03404 PMC7482304

[CR13] Cechinel MAP, Nicolini JL, Tápia PM, Miranda EAC, Eller S, de Oliveira TF, Raupp-Pereira F, Montedo ORK, Wermuth TB, Arcaro S (2023) Cobalt ferrite (CoFe_2_O_4_) spinel as a new efficient magnetic heterogeneous Fenton-like catalyst for wastewater treatment. Sustainability 15(20):15183. 10.3390/su152015183

[CR14] Charbonnet J, McDonough C, Xiao F, Schwichtenberg T, Cao D, Kaserzon S, Thomas K, Dewapriya P, Place B, Schymanski E, Field J, Helbling D, Higgins C (2022) Communicating confidence of per- and polyfluoroalkyl substance identification via high-resolution mass spectrometry. Environ Sci Technol Lett 9(6):473–481. 10.1021/acs.estlett.2c00206 35719859 10.1021/acs.estlett.2c00206 PMC9202347

[CR15] Chen D, Hale RC, Letcher RJ (2015a) Photochemical and microbial transformation of emerging flame retardants: cause for concern? Environ Toxicol Chem 34(4):687–699. 10.1002/etc.2858 25809099 10.1002/etc.2858

[CR16] Chen H, Zhang Z, Yang Z, Yang Q, Li B, Bai Z (2015b) Heterogeneous Fenton-like catalytic degradation of 2,4-dichlorophenoxyacetic acid in water with FeS. Chem Eng J 273:481–489. 10.1016/j.cej.2015.03.079

[CR17] Chen X, Chen F, Liu F, Yan X, Hu W, Zhang G, Tian L, Xia Q, Chen X (2016a) Ag nanoparticles/hematite mesocrystals superstructure composite: a facile synthesis and enhanced heterogeneous photo-Fenton activity. Catal Sci Technol 6(12):4184–4191. 10.1039/c6cy00080k

[CR18] Chen T, Liu Z, Yao J, Hao H, Chen F (2016b) Fenton‐like degradation comparison of * s * ‐triazine herbicides in aqueous medium. Clean-Soil Air Water 44(10):1315–1322. 10.1002/clen.201500304

[CR19] Chen W, Guo X, Wu M, Liu Z, Yang C, Xie H, Chen J (2024a) Novel magnetic nitrogen-doped biochar (NBC) as an activator for peroxymonosulfate to degrade naphthalene: emphasizing the synergistic effect between NBC and MnFe _2_O_4_. Sep Purif Technol 335:126263. 10.1016/j.seppur.2024.126263

[CR20] Chen C, Yuan Z, Sun S, Xie J, Zhang K, Zhai Y, Zuo R, Bi E, Tao Y, Song Q (2024b) Remediation of polycyclic aromatic hydrocarbon-contaminated soil by using activated persulfate with carbonylated activated carbon supported nanoscale zero-valent iron. Catalysts 14(5):311. 10.3390/catal14050311

[CR21] Chen Y, Wu F, Lin Y, Deng N, Bazhin N, Glebov E (2007) Photodegradation of glyphosate in the ferrioxalate system. J Hazard Mater 148(1–2):360–365. 10.1016/j.jhazmat.2007.02.044 17374441 10.1016/j.jhazmat.2007.02.044

[CR22] Cheng Y, Zhang S, Wang Z, Wang B, You J, Guo R, Zhang H (2023) Review on spinel ferrites-based materials (MFe_2_O_4_) as photo-Fenton catalysts for degradation of organic pollutants. Sep Purif Technol 318:123971. 10.1016/j.seppur.2023.123971

[CR23] Choi H, Kim D, Lee T (2013) Photochemical degradation of atrazine in UV and UV/H_2_O_2_-process: pathways and toxic effects of products. J Environ Sci Health B 48:927–934. 10.1080/03601234.2013.816587 23998304 10.1080/03601234.2013.816587

[CR24] Clarizia L, Russo D, Di Somma I, Marotta R, Andreozzi R (2017) Homogeneous photo-Fenton processes at near neutral pH: a review. Appl Catal B Environ 209:358–371. 10.1016/j.apcatb.2017.03.011

[CR25] Corma A, Concepción P, Serna P (2007) A different reaction pathway for the reduction of aromatic nitro compounds on gold catalysts. Angew Chem Int Ed Engl 46(38):7266–7269. 10.1002/anie.200700823 17579907 10.1002/anie.200700823

[CR26] Cornejo I, Hayes R (2021) A review of the critical aspects in the multi-scale modelling of structured catalytic reactors. Catalysts 11:89. 10.3390/catal11010089

[CR27] Cui K, Yi H, Zhou ZJ, Zhuo QF, Bing YX, Guo QW, Xu ZC (2014) Fenton oxidation kinetics and intermediates of nonylphenol ethoxylates. Environ Eng Sci 31(5):217–224. 10.1089/ees.2013.0308 24868141 10.1089/ees.2013.0308 PMC4027986

[CR28] Dai Y, Song Y, Wang S, Yuan Y (2015) Treatment of halogenated phenolic compounds by sequential tri-metal reduction and laccase-catalytic oxidation. Water Res 71:64–73. 10.1016/j.watres.2014.12.047 25596562 10.1016/j.watres.2014.12.047

[CR29] Danyliuk N, Husak V, Boichuk V, Ziółkowska D, Danyliuk I, Shyichuk A (2025) Kinetics of H_2_O_2_ decomposition and bacteria inactivation in a continuous-flow reactor with a fixed bed of cobalt ferrite catalyst. Appl Sci 15(15):8195. 10.3390/app15158195

[CR30] Danyliuk N, Lapchuk I, Husak V (2024) Toxicity of water treated with Fenton-like ferrite catalyst. Phys Chem Solid State 25:170–177. 10.15330/pcss.25.1.170-177

[CR31] Danyliuk N, Lapchuk I, Kotsyubynsky V, Boychuk V, Husak V (2023) Effect of Mn^2+^ substitution on catalytic properties of Fe_3_-xMnxO_4_ nanoparticles synthesized via co-precipitation method. Phys Chem Solid State 24:748–760. 10.15330/pcss.24.4.748-760

[CR32] de la Fuente L, Acosta T, Babay P, Curutchet G, Candal R, Litter MI (2010) Degradation of nonylphenol ethoxylate-9 (NPE-9) by photochemical advanced oxidation technologies. Ind Eng Chem Res 49(15):6909–6915. 10.1021/ie901785j

[CR33] Debnath B, Parvin S, Dixit H, Bhattacharyya S (2020) Oxygen‐defect‐rich cobalt ferrite nanoparticles for practical water electrolysis with high activity and durability. Chemsuschem 13(15):3875–3886. 10.1002/cssc.202000932 32469148 10.1002/cssc.202000932

[CR34] Degirmenci V, Rebrov E (2016) Design of catalytic micro trickle bed reactors. Physical Sciences Reviews 1:20150018. 10.1515/psr-2015-0018

[CR35] Deng J, Xu M, Qiu C, Chen Y, Ma X, Gao N, Li X (2018) Magnetic MnFe_2_O_4_ activated peroxymonosulfate processes for degradation of bisphenol A: performance, mechanism and application feasibility. Appl Surf Sci 459:138–147. 10.1016/j.apsusc.2018.07.198

[CR36] Ding X, Gutierrez L, Croue JP, Li M, Wang L, Wang Y (2020) Hydroxyl and sulfate radical-based oxidation of RhB dye in UV/H_2_O_2_ and UV/persulfate systems: kinetics, mechanisms, and comparison. Chemosphere 253:126655. 10.1016/j.chemosphere.2020.126655 32302899 10.1016/j.chemosphere.2020.126655

[CR37] Divyapriya G, Nidheesh P (2020) Importance of graphene in the electro-Fenton process. ACS Omega 5:4725–4732. 10.1021/acsomega.9b04201 32201757 10.1021/acsomega.9b04201 PMC7081297

[CR38] Dong CD, Tsai ML, Wang TH, Chang JH, Chen CW, Hung CM (2019a) Removal of polycyclic aromatic hydrocarbon (PAH)-contaminated sediments by persulfate oxidation and determination of degradation product cytotoxicity based on HepG2 and ZF4 cell lines. Environ Sci Pollut Res 27(28):34596–34605. 10.1007/s11356-019-04421-w 10.1007/s11356-019-04421-w 30746626

[CR39] Dong C, Qu Z, Qin Y, Fu Q, Sun H, Duan X (2019b) Revealing the highly catalytic performance of spinel CoMn2O4 for toluene oxidation: involvement and replenishment of oxygen species using * in situ * designed-TP techniques. ACS Catal 9:6698–6710. 10.1021/acscatal.9b01324

[CR40] Dung NT, Duong LT, Hoa NT, Thao VD, Ngan LV, Huy NN (2022) A comprehensive study on the heterogeneous electro-Fenton degradation of tartrazine in water using CoFe_2_O_4_/carbon felt cathode. Chemosphere 287:132141. 10.1016/j.chemosphere.2021.132141 34521013 10.1016/j.chemosphere.2021.132141

[CR41] Esteban García AB, Szymański K, Mozia S, Sánchez Pérez JA (2020) Treatment of laundry wastewater by solar photo-Fenton process at pilot plant scale. Environ Sci Pollut Res 28(7):8576–8584. 10.1007/s11356-020-11151-x 10.1007/s11356-020-11151-x PMC7854387 33064284

[CR42] Evgenidou E, Konstantinou I, Fytianos K, Poulios I (2007) Oxidation of two organophosphorous insecticides by the photo-assisted Fenton reaction. Water Res 41:2015–2027. 10.1016/j.watres.2007.01.027 17353026 10.1016/j.watres.2007.01.027

[CR43] Faheem, Du J, Kim SH, Hassan MA, Irshad S, Bao J (2020) Application of biochar in advanced oxidation processes: supportive, adsorptive, and catalytic role. Environ Sci Pollut Res 27(30):37286–37312. 10.1007/s11356-020-07612-y 10.1007/s11356-020-07612-y 31933079

[CR44] Flores A, Nesprias K, Vitale P, Tasca J, Lavat A, Eyler N, Cañizo A (2013) Heterogeneous photocatalytic discoloration/degradation of rhodamine B with H_2_O_2_ and spinel copper ferrite magnetic nanoparticles. Aust J Chem 67:609–614. 10.1071/ch13435

[CR45] Flotron V, Delteil C, Padellec Y, Camel V (2005) Removal of sorbed polycyclic aromatic hydrocarbons from soil, sludge and sediment samples using the Fenton’s reagent process. Chemosphere 59(10):1427–1437. 10.1016/j.chemosphere.2004.12.065 15876386 10.1016/j.chemosphere.2004.12.065

[CR46] Frolova LA, Khmelenko OV (2020) The study of Co–Ni-Mn ferrites for the catalytic decomposition of 4-nitrophenol. Catal Lett 151(5):1522–1533. 10.1007/s10562-020-03419-1

[CR47] Ganjali F, Kashtiaray A, Zarei-Shokat S, Taheri-Ledari R, Maleki A (2022) Functionalized hybrid magnetic catalytic systems on micro- and nanoscale utilized in organic synthesis and degradation of dyes. Nanoscale Adv 4(5):1263–1307. 10.1039/d1na00818h 36133673 10.1039/d1na00818h PMC9418160

[CR48] Gao H, Mao L, Li F, Xie L, Huang C, Shao J, Shao B, Kalyanaraman B, Zhu B (2017) Mechanism of intrinsic chemiluminescence production from the degradation of persistent chlorinated phenols by the Fenton system: a structure–activity relationship study and the critical role of quinoid and semiquinone radical intermediates. Environ Sci Technol 51(5):2934–2943. 10.1021/acs.est.6b04664 28128926 10.1021/acs.est.6b04664 PMC5806603

[CR49] Gao J, Proulx F, Rodriguez MJ (2019) Halogenated acetaldehydes in water: a review of their occurrence, formation, precursors and control strategies. Crit Rev Environ Sci Technol 49(15):1331–1385. 10.1080/10643389.2019.1571353

[CR50] Gao J, Wu S, Han Y, Tan F, Shi Y, Liu M, Li X (2018) 3D mesoporous CuFe_2_O_4_ as a catalyst for photo-Fenton removal of sulfonamide antibiotics at near neutral pH. J Colloid Interface Sci 524:409–416. 10.1016/j.jcis.2018.03.112 29677609 10.1016/j.jcis.2018.03.112

[CR51] García JC, Pedroza AM, Daza CE (2017) Magnetic Fenton and photo-Fenton-like catalysts supported on carbon nanotubes for wastewater treatment. Water Air Soil Pollut 228:246. 10.1007/s11270-017-3420-7

[CR52] Garcia-Muñoz P, Fresno F, de la Peña O’Shea VA, Keller N (2019) Ferrite materials for photoassisted environmental and solar fuels applications. Topics in Current Chemistry Collections 107–162:107. 10.1007/978-3-030-49492-6_4 10.1007/s41061-019-0270-3 31840192

[CR53] Gu S, Cui J, Liu F, Chen J (2023) Biochar loaded with cobalt ferrate activated persulfate to degrade naphthalene. RSC Adv 13:5283–5292. 10.1039/d2ra08120b 36777931 10.1039/d2ra08120b PMC9912118

[CR54] Guan Y, Ma J, Ren Y, Liu Y, Xiao J, Lin L, Zhang C (2013) Efficient degradation of atrazine by magnetic porous copper ferrite catalyzed peroxymonosulfate oxidation via the formation of hydroxyl and sulfate radicals. Water Res 47:5431–5438. 10.1016/j.watres.2013.06.023 23916710 10.1016/j.watres.2013.06.023

[CR55] Guan YH, Chen J, Chen LJ, Jiang XX, Fu Q (2020) Comparison of UV/H_2_O_2_, UV/PMS, and UV/PDS in destruction of different reactivity compounds and formation of bromate and chlorate. Front Chem 8:581198. 10.3389/fchem.2020.581198 33102448 10.3389/fchem.2020.581198 PMC7545204

[CR56] Guo X, Wang K, Li D, Qin J (2017) Heterogeneous photo-Fenton processes using graphite carbon coating hollow CuFe_2_O_4_ spheres for the degradation of methylene blue. Appl Surf Sci 420:792–801. 10.1016/j.apsusc.2017.05.178

[CR57] Guo Z, Si Y, Xia W, Wang F, Liu H, Yang C, Zhang W, Li W (2022) Electron delocalization triggers nonradical Fenton-like catalysis over spinel oxides. Proc Natl Acad Sci U S A 119:e2201607119. 10.1073/pnas.2201607119 35878043 10.1073/pnas.2201607119 PMC9351537

[CR58] Hariani PL, Said M, Rachmat A, Riyanti F, Pratiwi HC, Rizki WT (2021) Preparation of NiFe_2_O_4_ nanoparticles by solution combustion method as photocatalyst of Congo red. Bull Chem React Eng Catal 16(3):481–490. 10.9767/bcrec.16.3.10848.481-490

[CR59] Hassan H, Hameed B (2011) Fe–clay as effective heterogeneous Fenton catalyst for the decolorization of Reactive Blue 4. Chem Eng J 171(3):912–918. 10.1016/j.cej.2011.04.040

[CR60] Hassani A, Çelikdağ G, Eghbali P, Sevim M, Karaca S (2018) Metin heterogeneous sono-Fenton-like process using magnetic cobalt ferrite-reduced graphene oxide (CoFe_2_O_4_-rGO) nanocomposite for the removal of organic dyes from aqueous solution. Ultrason Sonochem 40:841–852. 10.1016/j.ultsonch.2017.08.026 28946495 10.1016/j.ultsonch.2017.08.026

[CR61] Heidari M, Varma R, Ahmadian M, Pourkhosravani M, Asadzadeh S, Karimi P, Khatami M (2019) Photo-Fenton like catalyst system: activated carbon/CoFe_2_O_4_ nanocomposite for reactive dye removal from textile wastewater. Appl Sci (Basel) 9(5):963. 10.3390/app9050963

[CR62] Heidari M, Vosoughi M, Sadeghi H, Dargahi A, Mokhtari S (2020) Degradation of diazinon from aqueous solutions by electro-Fenton process: effect of operating parameters, intermediate identification, degradation pathway, and optimization using response surface methodology (RSM). Sep Sci Technol 56:2287–2299. 10.1080/01496395.2020.1821060

[CR63] Hermosilla D, Han C, Nadagouda M, Machala L, Gascó A, Campo P, Dionysiou D (2020) Environmentally friendly synthesized and magnetically recoverable designed ferrite photo-catalysts for wastewater treatment applications. J Hazard Mater 381:121200. 10.1016/j.jhazmat.2019.121200 31563035 10.1016/j.jhazmat.2019.121200

[CR64] Hollender J, van Bavel B, Dulio V, Farmen E, Furtmann K, Koschorreck J, Kunkel U, Krauss M, Munthe J, Schlabach M, Slobodnik J, Stroomberg G, Ternes T, Thomaidis N, Togola A, Tornero V (2019) High resolution mass spectrometry-based non-target screening can support regulatory environmental monitoring and chemicals management. Environ Sci Eur 31(1):42. 10.1186/s12302-019-0225-x

[CR65] Hollender J, Schymanski E, Ahrens L, Alygizakis N, Béen F, Bijlsma L, Brunner A, Celma A, Fildier A, Fu Q, Gago-Ferrero P, Gil-Solsona R, Haglund P, Hansen M, Kaserzon S, Kruve A, Lamoree M, Margoum C, Meijer J, Merel S, Rauert C, Rostkowski P, Samanipour S, Schulze B, Schulze T, Singh R, Slobodnik J, Steininger-Mairinger S, Thomaidis N, Togola A, Vorkamp K, Vulliet E, Zhu L, Krauss M (2023) NORMAN guidance on suspect and non-target screening in environmental monitoring. Environ Sci Eur 35(1):75. 10.1186/s12302-023-00779-4

[CR66] Hu X, Liu B, Deng Y, Chen H, Luo S, Sun C, Yang P, Yang S (2011) Adsorption and heterogeneous Fenton degradation of 17α-methyltestosterone on nano Fe_3_O_4_/MWCNTs in aqueous solution. Appl Catal B Environ 107(3–4):274–283. 10.1016/j.apcatb.2011.07.025

[CR67] Huaccallo-Aguilar Y, Álvarez-Torrellas S, Martínez-Nieves J, Delgado-Adámez J, Gil M, Ovejero G, García J (2021) Magnetite-based catalyst in the catalytic wet peroxide oxidation for different aqueous matrices spiked with naproxen–diclofenac mixture. Catalysts 11(4):514. 10.3390/catal11040514

[CR68] Huang G, Wang C, Yang C, Guo P, Yu H (2017a) Degradation of bisphenol A by peroxymonosulfate catalytically activated with Mn_1.8_Fe_1.2_O_4_ nanospheres: synergism between Mn and Fe. Environ Sci Technol 51:12611–12618. 10.1021/acs.est.7b03007 28985472 10.1021/acs.est.7b03007

[CR69] Huang Y, Liang Y, Rao Y, Zhu D, Cao JJ, Shen Z, Ho W, Lee SC (2017b) Environment-friendly carbon quantum dots/ZnFe_2_O_4_ photocatalysts: characterization, biocompatibility, and mechanisms for NO removal. Environ Sci Technol 51(5):2924–2933. 10.1021/acs.est.6b04460 28145696 10.1021/acs.est.6b04460

[CR70] Huang M, Li Y, Zhang C, Cui C, Huang Q, Li M, Qiang Z, Zhou T, Wu X, Yu H (2022) Facilely tuning the intrinsic catalytic sites of the spinel oxide for peroxymonosulfate activation: from fundamental investigation to pilot-scale demonstration. Proc Natl Acad Sci U S A 119(30):e2202682119. 10.1073/pnas.2202682119 35858430 10.1073/pnas.2202682119 PMC9335229

[CR71] Hulleman T, Turkina V, O’Brien JW, Chojnacka A, Thomas KV, Samanipour S (2023) Critical assessment of the chemical space covered by LC–HRMS non-targeted analysis. Environ Sci Technol 57(38):14101–14112. 10.1021/acs.est.3c03606 37704971 10.1021/acs.est.3c03606 PMC10537454

[CR72] Husak V, Shvadchak V, Soltys L (2025) Catalyst-assisted H_2_O_2_ systems for bacterial disinfection of wastewater: from UV/visible synergy to electro-/photo-Fenton pathways. Phys Chem Solid State 26(4):738–751. 10.15330/pcss.26.4.738-751

[CR73] Ibrahim I, Ali IO, Salama TM, Bahgat A, Mohamed MM (2016) Synthesis of magnetically recyclable spinel ferrite (MFe2O4, M = Zn Co, Mn) nanocrystals engineered by sol gel-hydrothermal technology: high catalytic performances for nitroarenes reduction. Appl Catal B Environ 181:389–402. 10.1016/j.apcatb.2015.08.005

[CR74] Iurascu B, Siminiceanu I, Vione D, Vicente M, Gil A (2009) Phenol degradation in water through a heterogeneous photo-Fenton process catalyzed by Fe-treated laponite. Water Res 43:1313–1322. 10.1016/j.watres.2008.12.032 19138784 10.1016/j.watres.2008.12.032

[CR75] Ivanets A, Prozorovich V, Roshchina M, Grigoraviciute-Puroniene I, Zarkov A, Kareiva A, Wang Z, Srivastava V, Sillanpää M (2020) Heterogeneous Fenton oxidation using magnesium ferrite nanoparticles for ibuprofen removal from wastewater: optimization and kinetics studies. J Nanomater 2020:1–9. 10.1155/2020/8159628

[CR76] Ji Y, Niu J, Fang Y, Tan Nou A, Warsinger D (2022) Micelles inhibit electro-oxidation degradation of nonylphenol ethoxylates. Chem Eng J 430:133167. 10.1016/j.cej.2021.133167

[CR77] Jiang Z, Ma Y, Ke Q, Chu L, Guo C, Guo Y (2021) Hydrothermal deposition of CoFe_2_O_4_ nanoparticles on activated carbon fibers promotes atrazine removal via physical adsorption and photo-Fenton degradation. J Environ Chem Eng 9:105940. 10.1016/j.jece.2021.105940

[CR78] Joseph W (2016) Three-phase catalytic reactors for hydrogenation and oxidation reactions. Physical Sciences Reviews 1(1):20150019. 10.1515/psr-2015-0019

[CR79] Kahhki RM, Khorrampoor A, Rabbani M, Ahsani F (2017) Visible light photocatalytic degradation of textile waste water by Co doped NiFe_2_O_4_ nanocomposite. J Mater Sci Mater Electron 28:4095–4101. 10.1007/s10854-016-6028-6

[CR80] Kanel SR, Neppolian B, Jung H, Choi H (2004) Comparative removal of polycyclic aromatic hydrocarbons using iron oxide and hydrogen peroxide in soil slurries. Environ Eng Sci 21(6):741–751. 10.1089/ees.2004.21.741

[CR81] Keen O, Bolton J, Litter M, Bircher K, Oppenländer T (2018) Standard reporting of Electrical Energy per Order (EEO) for UV/H_2O2_ reactors (IUPAC Technical Report). Pure Appl Chem 90:1487–1499. 10.1515/pac-2017-0603

[CR82] Kotthoff L, Keller J, Lörchner D, Mekonnen TF, Koch M (2019) Transformation products of organic contaminants and residues—overview of current simulation methods. Molecules 24(4):753. 10.3390/molecules24040753 30791496 10.3390/molecules24040753 PMC6413221

[CR83] Králik M, Koóš P, Markovič M, Lopatka P (2025) Research and developments of heterogeneous catalytic technologies. Molecules 30(15):3279. 10.3390/molecules30153279 40807451 10.3390/molecules30153279 PMC12348656

[CR84] Li J, Xu M, Yao G, Lai B (2018) Enhancement of the degradation of atrazine through CoFe_2_O_4_ activated peroxymonosulfate (PMS) process: kinetic, degradation intermediates, and toxicity evaluation. Chem Eng J 348:1012–1024. 10.1016/j.cej.2018.05.032

[CR85] Li L, Jianhua W, Fang H (2025) Fabrication of ZnFe_2_O_4_@g-C_3_N_4_ for enhanced photo-Fenton effect and visible light-driven organic dye degradation. Sci Rep 15(1):21707. 10.1038/s41598-025-05096-9 40595994 10.1038/s41598-025-05096-9 PMC12217835

[CR86] Li R, Lu X, Gao J, Chen Y, Pan S (2023) Activation of peracetic acid by CoFe_2_O_4_ for efficient degradation of ofloxacin: reactive species and mechanism. Molecules 28:7906. 10.3390/molecules28237906 38067634 10.3390/molecules28237906 PMC10708156

[CR87] Li W, Orozco R, Camargos N, Liu H (2017) Mechanisms on the impacts of alkalinity, pH, and chloride on persulfate-based groundwater remediation. Environ Sci Technol 51:3948–3959. 10.1021/acs.est.6b04849 28263583 10.1021/acs.est.6b04849

[CR88] Li Y, Baghi R, Filip J, Islam S, Hope-weeks L, Yan W (2019) Activation of peroxydisulfate by ferrite materials for phenol degradation. ACS Sustain Chem Eng 7(9):8099–8108. 10.1021/acssuschemeng.8b05257

[CR89] Li Y, Chen Z, Qi J, Kang J, Shen J, Yan P, Wang W, Bi L, Zhang X, Zhu X (2021a) Degradation of bisphenol S by peroxymonosulfate activation through monodispersed CoFe_2_O_4_ nanoparticles anchored on natural palygorskite. Sep Purif Technol 277:119492. 10.1016/j.seppur.2021.119492

[CR90] Li Y, Zhu W, Guo Q, Wang X, Zhang L, Gao X, Luo Y (2021b) Highly efficient degradation of sulfamethoxazole (SMX) by activating peroxymonosulfate (PMS) with CoFe_2_O_4_ in a wide pH range. Sep Purif Technol 276:119403. 10.1016/j.seppur.2021.119403

[CR91] Liu K, Yu J, Dong H, Wu J, Hoffmann M (2018) Degradation and mineralization of carbamazepine using an electro-Fenton reaction catalyzed by magnetite nanoparticles fixed on an electrocatalytic carbon fiber textile cathode. Environ Sci Technol 52:12667–12674. 10.1021/acs.est.8b03916 30346735 10.1021/acs.est.8b03916 PMC6222555

[CR92] Liu S, Zada A, Yu X, Liu F, Jin G (2022) NiFe_2_O_4_/g-C_3_N_4_ heterostructure with an enhanced ability for photocatalytic degradation of tetracycline hydrochloride and antibacterial performance. Chemosphere 307:135717. 10.1016/j.chemosphere.2022.135717 35863405 10.1016/j.chemosphere.2022.135717

[CR93] Liu Y, Guo Z, Li F, Xiao Y, Zhang Y, Bu T, Jia P, Zhe T, Wang L (2019) Multifunctional magnetic copper ferrite nanoparticles as Fenton-like reaction and near-infrared photothermal agents for synergetic antibacterial therapy. ACS Appl Mater Interfaces 11:31649–31660. 10.1021/acsami.9b10096 31407880 10.1021/acsami.9b10096

[CR94] Liu Y, Liu L, Wang Y (2021) A critical review on removal of gaseous pollutants using sulfate radical-based advanced oxidation technologies. Environ Sci Technol 55(14):9691–9710. 10.1021/acs.est.1c01531 34191483 10.1021/acs.est.1c01531

[CR95] Lopes JP, Rodrigues AE (2016) Fixed-bed gas–solid catalytic reactors. Multiphase Catalytic Reactors 53–79:53. 10.1002/9781119248491.ch3

[CR96] Lopez-Arago N, Munoz M, de Pedro ZM, Casas JA (2024) Natural magnetite as an effective and long-lasting catalyst for CWPO of azole pesticides in a continuous up-flow fixed-bed reactor. Environ Sci Pollut Res 31(20):29148–29161. 10.1007/s11356-024-33065-8 10.1007/s11356-024-33065-8 PMC11058975 38568307

[CR97] Luo W, Huang W, Feng X, Huang Y, Song X, Lin H, Wang S, Mailhot G (2020) The utilization of Fe-doped g-C_3_N_4_ in a heterogeneous photo-Fenton-like catalytic system: the effect of different parameters and a system mechanism investigation. RSC Adv 10(37):21876–21886. 10.1039/d0ra00993h 35516634 10.1039/d0ra00993h PMC9054560

[CR98] Lushchak VI, Matviishyn TM, Husak VV, Storey JM, Storey KB (2018) Pesticide toxicity: a mechanistic approach. EXCLI J 17:1101–1136. 10.17179/excli2018-1710 30564086 10.17179/excli2018-1710 PMC6295629

[CR99] Lutze HV, Kerlin N, Schmidt TC (2015) Sulfate radical-based water treatment in presence of chloride: formation of chlorate, inter-conversion of sulfate radicals into hydroxyl radicals and influence of bicarbonate. Water Res 72:349–360. 10.1016/j.watres.2014.10.006 25455043 10.1016/j.watres.2014.10.006

[CR100] Lv X, Ma Y, Li Y, Yang Q (2020) Heterogeneous Fenton-like catalytic degradation of 2,4-dichlorophenoxyacetic acid by nano-scale zero-valent iron assembled on magnetite nanoparticles. Water 12(10):2909. 10.3390/w12102909

[CR101] Maji N, Dosanjh HS (2023) Ferrite nanoparticles as catalysts in organic reactions: a mini review. Magnetochemistry 9(6):156. 10.3390/magnetochemistry9060156

[CR102] Manos D, Miserli K, Konstantinou I (2020) Perovskite and spinel catalysts for sulfate radical-based advanced oxidation of organic pollutants in water and wastewater systems. Catalysts 10(11):1299. 10.3390/catal10111299

[CR103] Martins A, Wilde M, Vasconcelos T, Henriques D (2006) Nonylphenol polyethoxylate degradation by means of electrocoagulation and electrochemical Fenton. Sep Purif Technol 50:249–255. 10.1016/j.seppur.2005.11.032

[CR104] Menacherry S, Aravind U, Aravindakumar C (2022) Critical review on the role of mass spectrometry in the AOP based degradation of contaminants of emerging concern (CECs) in water. J Environ Chem Eng 10:108155. 10.1016/j.jece.2022.108155

[CR105] Miranzadeh MB, Zarjam R, Dehghani R, Haghighi M, Badi HZ, Marzaleh MA, Tehrani AM (2016) Comparison of Fenton and photo-Fenton processes for removal of linear alkyle benzene sulfonate (LAS) from aqueous solutions. Pol J Environ Stud 25(4):1639–1648. 10.15244/pjoes/61824

[CR106] Mishra S, Acharya L, Sharmila S, Sanjay K, Acharya R (2024) Designing g-C_3_N_4_/NiFe_2_O_4_ S-scheme heterojunctions for efficient photocatalytic degradation of Rhodamine B and tetracycline hydrochloride. Appl Surf Sci Adv 24:100647. 10.1016/j.apsadv.2024.100647

[CR107] Niu H, Zheng Y, Wang S, Zhao L, Yang S, Cai Y (2018) Continuous generation of hydroxyl radicals for highly efficient elimination of chlorophenols and phenols catalyzed by heterogeneous Fenton-like catalysts yolk/shell Pd@Fe_3_O_4_@metal organic frameworks. J Hazard Mater 346:174–183. 10.1016/j.jhazmat.2017.12.027 29274511 10.1016/j.jhazmat.2017.12.027

[CR108] Ou B, Wang J, Wu Y, Zhao S, Wang Z (2019) A highly efficient cathode based on modified graphite felt for aniline degradation by electro-Fenton. Chemosphere 235:49–57. 10.1016/j.chemosphere.2019.06.144 31255765 10.1016/j.chemosphere.2019.06.144

[CR109] Parvulescu V, Epron F, Garcia H, Granger P (2021) Recent progress and prospects in catalytic water treatment. Chem Rev 122:2981–3121. 10.1021/acs.chemrev.1c00527 34874709 10.1021/acs.chemrev.1c00527

[CR110] Pastor E, Sachs M, Selim S, Durrant JR, Bakulin AA, Walsh A (2022) Electronic defects in metal oxide photocatalysts. Nat Rev Mater 7(7):503–521. 10.1038/s41578-022-00433-0

[CR111] Patil S, Tamboli M, Deonikar V, Umarji G, Ambekar J, Kulkarni M, Kolekar S, Kale B, Patil D (2015) Magnetically separable Ag_3_PO_4_/NiFe_2_O_4_ composites with enhanced photocatalytic activity. Dalton Trans 44:20426–20434. 10.1039/c5dt03173g 26508302 10.1039/c5dt03173g

[CR112] Poomipuen K, Sakulthaew C, Chokejaroenrat C, Angkaew A, Techauay K, Poompoung T, Teingtham K, Phansak P, Lueangjaroenkit P, Snow D (2023) Dual activation of peroxymonosulfate using MnFe_2_O_4_/g-C_3_N_4_ and visible light for the efficient degradation of steroid hormones: performance, mechanisms, and environmental impacts. ACS Omega 8:36136–36151. 10.1021/acsomega.3c04333 37810650 10.1021/acsomega.3c04333 PMC10552087

[CR113] Prasse C, Ford B, Nomura DK, Sedlak DL (2018) Unexpected transformation of dissolved phenols to toxic dicarbonyls by hydroxyl radicals and UV light. Proc Natl Acad Sci U S A 115(10):2311–2316. 10.1073/pnas.1715821115 29463747 10.1073/pnas.1715821115 PMC5877969

[CR114] Raveendran A, Semasko E, Jagminas A, Sukoviene A, Ramanavicius S (2026) Current trends in cobalt and zinc ferrite based magnetic photocatalysts for wastewater treatment. Inorg Chem Commun 183:115876. 10.1016/j.inoche.2025.115876

[CR115] Remya KP, Rajalakshmi R, Ponpandian N (2020) Development of BiFeO₃/MnFe₂O₄ ferrite nanocomposites with enhanced magnetic and electrical properties. Nanoscale Adv 2(7):2968–2976. 10.1039/d0na00255k 36132389 10.1039/d0na00255k PMC9418487

[CR116] Ren W, Cheng C, Shao P, Luo X, Zhang H, Wang S, Duan X (2021) Origins of electron-transfer regime in persulfate-based nonradical oxidation processes. Environ Sci Technol 56:78–97. 10.1021/acs.est.1c05374 34932343 10.1021/acs.est.1c05374

[CR117] Rey A, Hungria A, Duran-Valle C, Faraldos M, Bahamonde A, Casas J, Rodriguez J (2016) On the optimization of activated carbon-supported iron catalysts in catalytic wet peroxide oxidation process. Appl Catal B Environ 181:249–259. 10.1016/j.apcatb.2015.07.051

[CR118] Ribeiro R, Gallo J, Bañobre-López M, Silva A, Faria J, Gomes H (2019) Enhanced performance of cobalt ferrite encapsulated in graphitic shell by means of AC magnetically activated catalytic wet peroxide oxidation of 4-nitrophenol. Chem Eng J 376:120012. 10.1016/j.cej.2018.09.173

[CR119] Richardson SD, Postigo C (2015) Formation of DBPs: state of the science. ACS Symposium Series 189–214:189. 10.1021/bk-2015-1190.ch011

[CR120] Rodríguez A, Ovejero G, Sotelo JL, Mestanza M, García J (2009) Heterogeneous Fenton catalyst supports screening for mono azo dye degradation in contaminated wastewaters. Ind Eng Chem Res 49(2):498–505. 10.1021/ie901212m

[CR121] Routoula E, Patwardhan SV (2020) Degradation of anthraquinone dyes from effluents: a review focusing on enzymatic dye degradation with industrial potential. Environ Sci Technol 54(2):647–664. 10.1021/acs.est.9b03737 31913605 10.1021/acs.est.9b03737

[CR122] Salih S, Mahmood W (2023) Review on magnetic spinel ferrite (MFe2O4) nanoparticles: from synthesis to application. Heliyon 9:e16601. 10.1016/j.heliyon.2023.e16601 37274649 10.1016/j.heliyon.2023.e16601 PMC10238938

[CR123] Scaria J, Nidheesh PV (2022) Comparison of hydroxyl-radical-based advanced oxidation processes with sulfate radical-based advanced oxidation processes. Curr Opin Chem Eng 36:100830. 10.1016/j.coche.2022.100830

[CR124] Schmiemann D, Hohenschon L, Bartels I, Hermsen A, Bachmann F, Cordes A, Jäger M, Gutmann J, Hoffmann-Jacobsen K (2023) Enzymatic post-treatment of ozonation: laccase-mediated removal of the by-products of acetaminophen ozonation. Environ Sci Pollut Res 30(18):53128–53139. 10.1007/s11356-023-25913-w 10.1007/s11356-023-25913-w PMC10119220 36853537

[CR125] Sebah I, Belmouden M (2025) Copper ferrite–graphene oxide catalyst for enhanced peroxymonosulfate activation and pollutant degradation. Nanoscale Adv 7:5646–5657. 10.1039/D5NA00409H 40801044 10.1039/d5na00409h PMC12336850

[CR126] Sharma R, Bansal S, Singhal S (2015) Tailoring the photo-Fenton activity of spinel ferrites (MFe_2_O_4_) by incorporating different cations (M = Cu, Zn, Ni and Co) in the structure. RSC Adv 5(8):6006–6018. 10.1039/c4ra13692f

[CR127] Sharma R, Singhal S (2018) Spinel ferrite mediated photo-Fenton degradation of phenolic analogues: a detailed study employing two distinct inorganic oxidants. Clean: Soil, Air, Water 46:1700605. 10.1002/clen.201700605

[CR128] Sheng J, Xu J, Qin B, Jiang H (2022) Three-dimensional flower-like magnetic CoFe-LDHs/CoFe_2_O_4_ composites activating peroxymonosulfate for high efficient degradation of aniline. J Environ Manage 310:114693. 10.1016/j.jenvman.2022.114693 35189554 10.1016/j.jenvman.2022.114693

[CR129] Shi K, Qiu F, Wang P, Li H, Wang C (2022) Magnetic MgFe_2_O_4_/MIL-88A catalyst for photo-Fenton sulfamethoxazole decomposition under visible light. Sep Purif Technol 301:121965. 10.1016/j.seppur.2022.121965

[CR130] Silva E, Brasileiro I, Madeira V, de Farias B, Ramalho M, Rodríguez-Aguado E, Rodríguez-Castellón E (2020) Reusable CuFe_2_O_4_–Fe_2_O_3_ catalyst synthesis and application for the heterogeneous photo-Fenton degradation of methylene blue in visible light. J Environ Chem Eng 8(5):104132. 10.1016/j.jece.2020.104132

[CR131] Song J, Wang Y, Lv Z, Li Y, Cao X, Cheng W (2023) Degradation of nonylphenol ethoxylate 10 in biochar-CoFe_2_O_4_/peroxymonosulfate system: transformation products identification, catalysis mechanism and influencing factors. J Environ Chem Eng 11:109241. 10.1016/j.jece.2022.109241

[CR132] Souza DRD, Trovó AG, Antoniosi Filho NR, Silva MAA, Machado AEH (2013) Degradation of the commercial herbicide glyphosate by photo-Fenton process: evaluation of kinetic parameters and toxicity. J Braz Chem Soc 24(9):1451–1460. 10.5935/0103-5053.20130185

[CR133] Suman TY, Kim SY, Yeom DH, Jeon J (2022) Transformation products of emerging pollutants explored using non-target screening: perspective in the transformation pathway and toxicity mechanism—a review. Toxics 10(2):54. 10.3390/toxics10020054 35202240 10.3390/toxics10020054 PMC8874687

[CR134] Sun S, Zeng X, Lemley A (2013) Nano-magnetite catalyzed heterogeneous Fenton-like degradation of emerging contaminants carbamazepine and ibuprofen in aqueous suspensions and montmorillonite clay slurries at neutral pH. J Mol Catal A Chem 371:94–103. 10.1016/j.molcata.2013.01.027

[CR135] Szymańska K, Ciemięga A, Maresz K, Pudło W, Malinowski J, Mrowiec-Białoń J, Jarzębski A (2021) Catalytic functionalized structured monolithic micro-/mesoreactors: engineering, properties, and performance in flow synthesis: an overview and guidelines. Front Chem Eng 3:789102. 10.3389/fceng.2021.789102

[CR136] Tabasum A, Alghuthaymi M, Qazi U, Shahid I, Abbas Q, Javaid R, Nadeem N, Zahid M (2020a) UV-accelerated photocatalytic degradation of pesticide over magnetite and cobalt ferrite decorated graphene oxide composite. Plants (Basel) 10(1):6. 10.3390/plants10010006 33374688 10.3390/plants10010006 PMC7822422

[CR137] Tabasum A, Bhatti I, Nadeem N, Zahid M, Rehan Z, Hussain T, Jilani A (2020b) Degradation of acetamiprid using graphene-oxide-based metal (Mn and Ni) ferrites as Fenton-like photocatalysts. Water Sci Technol 81:178–189. 10.2166/wst.2020.098 32293601 10.2166/wst.2020.098

[CR138] Tan C, Gao N, Fu D, Deng J, Deng L (2017) Efficient degradation of paracetamol with nanoscaled magnetic CoFe_2_O_4_ and MnFe_2_O_4_ as a heterogeneous catalyst of peroxymonosulfate. Sep Purif Technol 175:47–57. 10.1016/j.seppur.2016.11.016

[CR139] Tatarchuk T (2024) Studying the defects in spinel compounds: discovery, formation mechanisms, classification, and influence on catalytic properties. Nanomaterials (Basel) 14(20):1640. 10.3390/nano14201640 39452977 10.3390/nano14201640 PMC11510202

[CR140] Tatarchuk T, Bilovol V, Shyichuk A, Danyliuk I, Sokołowski K, Gajewska M (2025) Mesoporous Co-Mn ferrites as highly radical-forming catalysts for wet peroxide oxidation of 4-nitrophenol. Appl Surf Sci 690:162610. 10.1016/j.apsusc.2025.162610

[CR141] Tatarchuk T, Shyichuk A, Danyliuk N, Lapchuk I, Husak V, Macyk W (2024) Fenton-like water disinfection using fixed-bed reactor filled with a CoFe_2_O_4_ catalyst: mechanisms, the impact of anions, electromagnetic heating, and toxicity evaluation. Sep Purif Technol 348:127748. 10.1016/j.seppur.2024.127748

[CR142] Thirumoorthy K, Gokulakrishnan B, Satishkumar G, Landau M, Man M, Oliviero E (2021) Al-doped magnetite encapsulated in mesoporous carbon: a long-lasting Fenton catalyst for CWPO of phenol in a fixed-bed reactor under mild conditions. Catal Sci Technol 11:7368–7379. 10.1039/d1cy01218e

[CR143] Thomas N, Dionysiou DD, Pillai SC (2021) Heterogeneous Fenton catalysts: a review of recent advances. J Hazard Mater 404:124082. 10.1016/j.jhazmat.2020.124082 33069994 10.1016/j.jhazmat.2020.124082 PMC7530584

[CR144] Tian K, Shi F, Cao M, Zheng Q, Zhang G (2022) A review of persulfate activation by magnetic catalysts to degrade organic contaminants: mechanisms and applications. Catalysts 12:1058. 10.3390/catal12091058

[CR145] Tian Y, Liu Q, Lin S, Liang X, Khan S, Yang X, Wang X (2023) Magnetic Z-scheme CuFe_2_O_4_/MIL-101(Fe) toward chlorpyrifos degradation: photocatalytic mechanism, degradation pathways, and intermediates toxicity evaluation. J Environ Chem Eng 11:110054. 10.1016/j.jece.2023.110054

[CR146] Tran C, La D, Thi Hoai P, Ninh H, Thi Hong P, Vu T, Nadda A, Nguyen X, Nguyen D, Ngo H (2021) New TiO_2_-doped Cu–Mg spinel-ferrite-based photocatalyst for degrading highly toxic rhodamine B dye in wastewater. J Hazard Mater 420:126636. 10.1016/j.jhazmat.2021.126636 34280722 10.1016/j.jhazmat.2021.126636

[CR147] Tran MH, Nguyen HC, Le TS, Dang VAD, Cao TH, Le CK, Dang TD (2019) Degradation of glyphosate herbicide by an electro-Fenton process using carbon felt cathode. Environ Technol 42(8):1155–1164. 10.1080/09593330.2019.1660411 31469339 10.1080/09593330.2019.1660411

[CR148] Tsvetkov M, Ivanova I, Valcheva E, Zaharieva J, Milanova M (2019) Photocatalytic activity of NiFe_2_O_4_ and Zn_0.5_Ni_0.5_Fe_2_O_4_ modified by Eu(III) and Tb(III) for decomposition of malachite green. Open Chem 17:1124–1132. 10.1515/chem-2019-0116

[CR149] Usman M, Faure P, Ruby C, Hanna K (2012) Remediation of PAH-contaminated soils by magnetite catalyzed Fenton-like oxidation. Appl Catal B Environ 117–118:10–17. 10.1016/j.apcatb.2012.01.007

[CR150] Van N, Phan A, Cuong V, Van N, Thanh H, Khai N, Tri N, Nguyen T, Bui X, Huynh K (2023) Enhanced heterogeneous Fenton degradation of p-nitrophenol by FeO nanoparticles decorated cellulose aerogel from banana stem. Environ Technol Innov 30:103041. 10.1016/j.eti.2023.103041

[CR151] Varghese D, Ruban MJR, Jennifer PJS, AnnieCanisius D, Chakko S, Muthupandi S, Madhavana J, Raj MVA (2023) Comprehensive analysis of NiFe₂O₄/MWCNTs nanocomposite to degrade a healthcare waste – tetracycline. RSC Adv 13:28339–28361. 10.1039/d3ra05398a 37767116 10.1039/d3ra05398a PMC10520693

[CR152] Villegas LGC, Mashhadi N, Chen M, Mukherjee D, Taylor KE, Biswas N (2016) A short review of techniques for phenol removal from wastewater. Curr Pollut Rep 2(3):157–167. 10.1007/s40726-016-0035-3

[CR153] Vitale C, Lommen A, Huber C, Wagner K, Garlito Molina B, Nijssen R, Price E, Blokland M, van Tricht F, Mol H, Krauss M, Debrauwer L, Pardo O, Leon N, Klanova J, Antignac J (2022) Harmonized quality assurance/quality control provisions for nontargeted measurement of urinary pesticide biomarkers in the HBM4EU multisite SPECIMEn study. Anal Chem 94(22):7833–7843. 10.1021/acs.analchem.2c00061 35616234 10.1021/acs.analchem.2c00061

[CR154] Vosough M, Schmidt TC, Renner G (2024) Non-target screening in water analysis: recent trends of data evaluation, quality assurance, and their future perspectives. Anal Bioanal Chem 416(9):2125–2136. 10.1007/s00216-024-05153-8 38300263 10.1007/s00216-024-05153-8 PMC10951028

[CR155] Wang J, Yao Z, Yang M, Wang Y, Xia Q, Jiang Z (2016) A Fe_3_O_4_/FeAl_2_O_4_ composite coating via plasma electrolytic oxidation on Q_235_ carbon steel for Fenton-like degradation of phenol. Environ Sci Pollut Res 23:14927–14936. 10.1007/s11356-016-6613-5 10.1007/s11356-016-6613-5 27074928

[CR156] Wang X, Zhang X, Zhang Y, Wang Y, Sun S, Wu W, Wu Z (2020) Nanostructured semiconductor supported iron catalysts for heterogeneous photo-Fenton oxidation: a review. J Mater Chem A 8(31):15513–15546. 10.1039/d0ta04541a

[CR157] Wang K, Han C, Shao Z, Qiu J, Wang S, Liu S (2021a) Perovskite oxide catalysts for advanced oxidation reactions. Adv Funct Mater 31(30):2102089. 10.1002/adfm.202102089

[CR158] Wang Y, Wang H, Yang Y, Xin B (2021b) Magnetic NiFe_2_O_4_ 3D nanosphere photocatalyst: glycerol-assisted microwave solvothermal synthesis and photocatalytic activity under microwave electrodeless discharge lamp. Ceram Int 47:14594–14602. 10.1016/j.ceramint.2021.02.041

[CR159] Xia X, Zhu F, Li J, Yang H, Wei L, Li Q, Jiang J, Zhang G, Zhao Q (2020) A review study on sulfate-radical-based advanced oxidation processes for domestic/industrial wastewater treatment: degradation, efficiency, and mechanism. Front Chem 8:592056. 10.3389/fchem.2020.592056 33330379 10.3389/fchem.2020.592056 PMC7729018

[CR160] Xian G, Kong S, Li Q, Zhang G, Zhou N, Du H, Niu L (2020) Synthesis of spinel ferrite MFe_2_O_4_ (M = Co, Cu, Mn, and Zn) for persulfate activation to remove aqueous organics: effects of M-site metal and synthetic method. Front Chem 8:177. 10.3389/fchem.2020.00177 32266209 10.3389/fchem.2020.00177 PMC7105867

[CR161] Xiang W, Chang J, Qu R, Albasher G, Wang Z, Zhou D, Sun C (2021) Transformation of bromophenols by aqueous chlorination and exploration of main reaction mechanisms. Chemosphere 265:129112. 10.1016/j.chemosphere.2020.129112 33288278 10.1016/j.chemosphere.2020.129112

[CR162] Xiang Y, Huang Y, Xiao B, Wu X, Zhang G (2020) Magnetic yolk-shell structure of ZnFe_2_O_4_ nanoparticles for enhanced visible light photo-Fenton degradation towards antibiotics and mechanism study. Appl Surf Sci 513:145820. 10.1016/j.apsusc.2020.145820

[CR163] Xiangwei Y, Graells M, Miralles-Cuevas S, Cabrera-Reina A, Pérez-Moya M (2021) An improved hybrid strategy for online dosage of hydrogen peroxide in photo-Fenton processes. J Environ Chem Eng 9(3):105235. 10.1016/j.jece.2021.105235 10.3390/ijerph182413313 PMC8701871 34948924

[CR164] Xie H, Xu W (2019) Enhanced activation of persulfate by meso-CoFe_2_O_4_/SiO_2_ with ultrasonic treatment for degradation of chlorpyrifos. ACS Omega 4:17177–17185. 10.1021/acsomega.9b01626 31656891 10.1021/acsomega.9b01626 PMC6811850

[CR165] Xie Z, He C, Zhou H, Li L, Liu Y, Du Y, Liu W, Mu Y, Lai B (2022) Effects of molecular structure on organic contaminants’ degradation efficiency and dominant ROS in the advanced oxidation process with multiple ROS. Environ Sci Technol 56(12):8784–8795. 10.1021/acs.est.2c00464 35584301 10.1021/acs.est.2c00464

[CR166] Xu Y, Ai J, Zhang H (2016) The mechanism of degradation of bisphenol A using the magnetically separable CuFe2O4/peroxymonosulfate heterogeneous oxidation process. J Hazard Mater 309:87–96. 10.1016/j.jhazmat.2016.01.023 26875144 10.1016/j.jhazmat.2016.01.023

[CR167] Yan Y, Wei Z, Duan X, Long M, Spinney R, Dionysiou D, Xiao R, Alvarez P (2023) Merits and limitations of radical vs. nonradical pathways in persulfate-based advanced oxidation processes. Environ Sci Technol 57(33):12153–12179. 10.1021/acs.est.3c05153 37535865 10.1021/acs.est.3c05153

[CR168] Yang Y, Gu Y, Lin H, Jie B, Zheng Z, Zhang X (2022) Bicarbonate-enhanced iron-based Prussian blue analogs catalyze the Fenton-like degradation of p-nitrophenol. J Colloid Interface Sci 608:2884–2895. 10.1016/j.jcis.2021.11.015 34802757 10.1016/j.jcis.2021.11.015

[CR169] Yao Y, Cai Y, Lu F, Qin J, Wei F, Xu C, Wang S (2014) Magnetic ZnFe_2_O_4_–C_3_N_4_ hybrid for photocatalytic degradation of aqueous organic pollutants by visible light. Ind Eng Chem Res 53(44):17294–17302. 10.1021/ie503437z

[CR170] Yi Q, Li Y, Dai R, Li X, Li Z, Wang Z (2022) Efficient removal of neonicotinoid by singlet oxygen dominated MoSx/ceramic membrane-integrated Fenton-like process. J Hazard Mater 439:129672. 10.1016/j.jhazmat.2022.129672 36104901 10.1016/j.jhazmat.2022.129672

[CR171] Yoom H, Shin J, Ra J, Son H, Ryu D, Kim C, Lee Y (2018) Transformation of methylparaben during water chlorination: effects of bromide and dissolved organic matter on reaction kinetics and transformation pathways. Sci Total Environ 634:677–686. 10.1016/j.scitotenv.2018.03.330 29642049 10.1016/j.scitotenv.2018.03.330

[CR172] Yu X, Somoza-Tornos A, Graells M, Pérez-Moya M (2020) An experimental approach to the optimization of the dosage of hydrogen peroxide for Fenton and photo-Fenton processes. Sci Total Environ 743:140402. 10.1016/j.scitotenv.2020.140402 32758807 10.1016/j.scitotenv.2020.140402

[CR173] Yuan T, Ding S, Xue F, Du Z, Yang X, Han Q, Ma M, Chen X (2024) Reactivity and reaction pathways of peroxymonosulfate and peroxydisulfate with neonicotinoid insecticides. Water Res 248:120852. 10.1016/j.watres.2023.120852 37976950 10.1016/j.watres.2023.120852

[CR174] Zahn D, Arp H, Fenner K, Georgi A, Hafner J, Hale S, Hollender J, Letzel T, Schymanski E, Sigmund G, Reemtsma T (2024) Should transformation products change the way we manage chemicals? Environ Sci Technol 58(18):7710–7718. 10.1021/acs.est.4c00125 38656189 10.1021/acs.est.4c00125 PMC11080041

[CR175] Zhang F, Hou J, Miao L, Chen J, Xu Y, You G, Liu S, Ma J (2018) Chlorpyrifos and 3,5,6-trichloro-2-pyridinol degradation in zero valent iron coupled anaerobic system: performances and mechanisms. Chem Eng J 353:254–263. 10.1016/j.cej.2018.07.136

[CR176] Zhang G, Sun Y, Gao DZ, Xu Y (2010) Quasi-cube ZnFe_2_O_4_ nanocrystals: hydrothermal synthesis and photocatalytic activity with TiO_2_ (Degussa P_25_) as nanocomposite. Mater Res Bull 45:755–760. 10.1016/j.materresbull.2010.03.025

[CR177] Zhang J, Sha S, Ni J, Wang Y, Wu Y, Hart J, Ferry M, Liu B, Li W (2025) Design strategies for high entropy materials in water electrolysis: enhancing activity, stability, and reaction kinetics. J Adv Ceram 14(10):9221152. 10.26599/jac.2025.9221152

[CR178] Zhang X, He M, Liu J, Liao R, Zhao L, Xie J, Wang R, Yang S, Wang H, Liu Y (2014) Fe_3_O_4_@C nanoparticles as high-performance Fenton-like catalyst for dye decoloration. Chin Sci Bull 59(27):3406–3412. 10.1007/s11434-014-0439-7

[CR179] Zhang Z, Ouyang Z, Yang J, Liu Y, Yang C, Dang Z (2019) High mineral adsorption of glyphosate versus diethyl phthalate and tetracycline, during visible light photodegradation with goethite and oxalate. Environ Chem Lett 17(3):1421–1428. 10.1007/s10311-019-00877-x

[CR180] Zhao R, Chen D, Liu H, Tian H, Li R, Huang Y (2024) FePO_4_/WB as an efficient heterogeneous Fenton-like catalyst for rapid removal of neonicotinoid insecticides: ROS quantification, mechanistic insights and degradation pathways. J Hazard Mater 476:135068. 10.1016/j.jhazmat.2024.135068 39002487 10.1016/j.jhazmat.2024.135068

[CR181] Zhi Z, Wu D, Meng F, Yin Y, Song B, Zhao Y, Song M (2022) Facile synthesis of CoFe2O4@BC activated peroxymonosulfate for p-nitrochlorobenzene degradation: matrix effect and toxicity evaluation. Sci Total Environ 828:154275. 10.1016/j.scitotenv.2022.154275 35248636 10.1016/j.scitotenv.2022.154275

[CR182] Zhu H, Guo A, Xian L, Wang Y, Long Y, Fan G (2022) Facile fabrication of surface vulcanized Co-Fe spinel oxide nanoparticles toward efficient 4-nitrophenol destruction. J Hazard Mater 430:128433. 10.1016/j.jhazmat.2022.128433 35158244 10.1016/j.jhazmat.2022.128433

[CR183] Zhu H, Ma H, Zhao Z, Xu L, Li M, Liu W, Lai B, Vithanage M, Pu S (2025) Electron transfer tuning for persulfate activation via the radical and non-radical pathways with biochar mediator. J Hazard Mater 486:136825. 10.1016/j.jhazmat.2024.136825 39721476 10.1016/j.jhazmat.2024.136825

